# Design, Synthesis, and Antimicrobial Evaluation of New Thiopyrimidine–Benzenesulfonamide Compounds

**DOI:** 10.3390/molecules29194778

**Published:** 2024-10-09

**Authors:** Abdalrahman Khalifa, Manal M. Anwar, Walaa A. Alshareef, Eman A. El-Gebaly, Samia A. Elseginy, Sameh H. Abdelwahed

**Affiliations:** 1 Department of Chemistry, Prairie View A&M University, Prairie View, TX 77446, USA; akhalifa@tamu.edu; 2 Department of Chemistry, Texas A&M University, College Station, TX 77843, USA; 3 Department of Therapeutic Chemistry, Pharmaceutical and Drug Industries Research Institute, National Research Centre, Dokki, Cairo P.O. Box 12622, Egypt; manal.hasan52@live.com; 4 Microbiology and Immunology Department, Faculty of Pharmacy, O6U, Giza P.O. Box 12585, Egypt; lolowation@gmail.com (W.A.A.); eman.elgabali.pha@o6u.edu.eg (E.A.E.-G.); 5 Green Chemistry Department, Chemical Industries Research Institute, National Research Centre, Cairo P.O. Box 12622, Egypt; samiaaliph@gmail.com

**Keywords:** thiopyrimidine, benzenesulfonamides, antimicrobial activity, bactericidal activity, anti-virulence activity, in silico ADMET

## Abstract

Bacterial infection poses a serious threat to human life due to the rapidly growing resistance of bacteria to antibacterial drugs, which is a significant public health issue. This study was focused on the design and synthesis of a new series of 25 analogues bearing a 5-cyano-6-oxo-4-substituted phenyl-1,6-dihydropyrimidine scaffold hybridized with different substituted benzenesulfonamides through the thioacetamide linker **M1–25**. The antimicrobial activity of the new molecules was studied against various Gram-positive, Gram-negative, and fungal strains. All the tested compounds showed promising broad-spectrum antimicrobial efficacy, especially against *K. pneumoniae* and *P. aeruginosa*. Furthermore, the most promising compounds, **6M**, **19M**, **20M**, and **25M,** were subjected to minimum inhibitory concentration (MIC) and minimum bactericidal concentration (MBC) assays. In addition, the antivirulence activity of the compounds was also examined using multiple biofilm assays. The new compounds promisingly revealed the suppression of microbial biofilm formation in the examined *K. pneumoniae* and *P. aeruginosa* microbial isolates. Additionally, in silico ADMET studies were conducted to determine their oral bioavailability, drug-likeness characteristics, and human toxicity risks. It is suggested that new pyrimidine–benzenesulfonamide derivatives may serve as model compounds for the further optimization and development of new antimicrobial and antisepsis candidates.

## 1. Introduction

The persistent spread of microbial diseases resistant to conventional therapies poses a serious threat to global public health. According to estimates, antibiotic-resistant pathogenic bacterial infections claim the lives of 700,000 people worldwide each year. Without innovative methods for prevention or treatment, projections indicate that these infectious diseases will claim the lives of 10 million people annually by 2050 [1,2].

Multi-drug-resistant (MDR) bacteria have emerged as a result of illness exposure in hospitals, overconsumption, and inappropriate antibiotic use [3,4]. One of the main risks to global health, according to the World Health Organization (WHO), is antibiotic resistance. To treat resistant infections, it is imperative to find and develop new antibiotics. The use of natural products, the repurposing of current medications, and the creation of novel synthetic compounds are some of the tactics being investigated in the search for new antimicrobial agents [5,6].

Bacteria living in biofilms, not free bacteria, are known to cause antibiotic-resistant bacterial infections. Researchers believe that biofilm-forming bacteria develop resistance to traditional antimicrobials due to three factors: (1) the antimicrobials’ inability to penetrate biofilms; (2) the emergence of complex drug resistance characteristics; and (3) the biofilms’ deactivation or modification of antimicrobial enzymes [7]. Given the increased prevalence of life-threatening diseases, a common goal is to create pharmaceutical regimens with improved antibacterial properties that offer more consistency and efficiency against infections by resistant bacterial pathogens [8].

**Figure 1:**
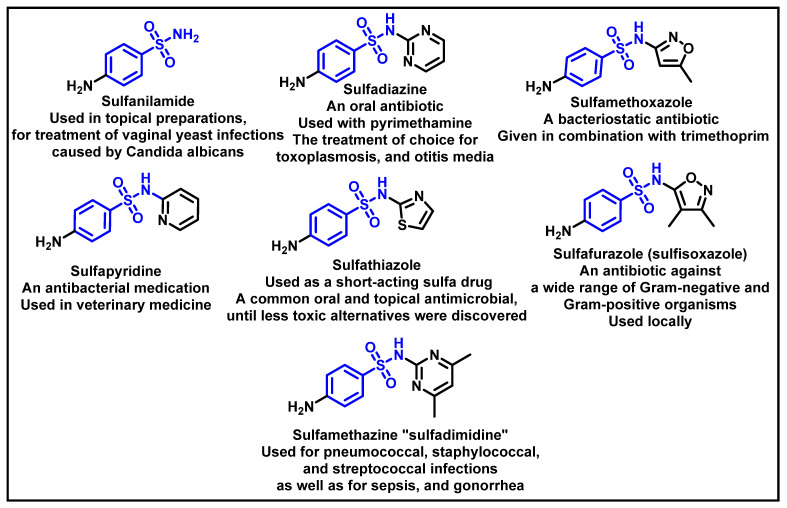
Examples of various FDA-approved sulfonamide antibiotics.

Sulfonamides were the earliest developed class of synthetic antibacterial medications (Figure 1). Since the 1930s, they have been used in pharmaceutical therapeutics and have proven to be effective against a variety of pathogens and clinical infections [9].

Drugs classified as classical sulfonamides inhibit dihydropteroate synthase (DHPS). They compete with its natural substrate, PABA, thereby blocking folate biosynthesis and subsequently leading to defective thymidine biosynthesis. It has also been documented that sulfonamides interfere with the development of peptidoglycan in a variety of pathogens by blocking specific enzymes (MurB, MurD, and MurE) involved in its biosynthesis. Moreover, sulfonamides have the ability to suppress serine/threonine kinase (Stk1/PknB), resulting in the increased sensitivity of MRSA to sublethal concentrations of β-lactams, thus reversing acquired resistance [9,10]. Another mode of action of sulfonamides is the inhibition of carbonic anhydrases (CAs) that participate in pH regulation and CO_2_ and bicarbonate-dependent biosynthetic pathways by catalyzing the interconversion between these two small molecules [11]. Figure 1 represents various FDA-approved sulfonamide antibiotics.

It is well recognized that several heterocyclic scaffolds serve as essential structural components in the majority of the world’s most widely prescribed medicinal pharmaceuticals. Among the heterocyclic substances with biological significance are derivatives of pyrimidines and pyrimidine–carbonitriles [12,13,14,15,16]. The pyrimidine moiety is a crucial component of many physiologically active substances that occur naturally and is involved in both chemical and biological activities [17]. A pyrimidine-based nucleotide that functions as a prosthetic scaffold for several enzymes is involved in a variety of redox reactions in living beings [18].

Researchers have focused on a broad range of pyrimidine–carbonitrile ligands for many years, and these compounds have demonstrated significant roles as physiologically active drugs with antibacterial, antiviral, anticancer, and other properties [19,20,21,22,23,24].

**Figure 2:**
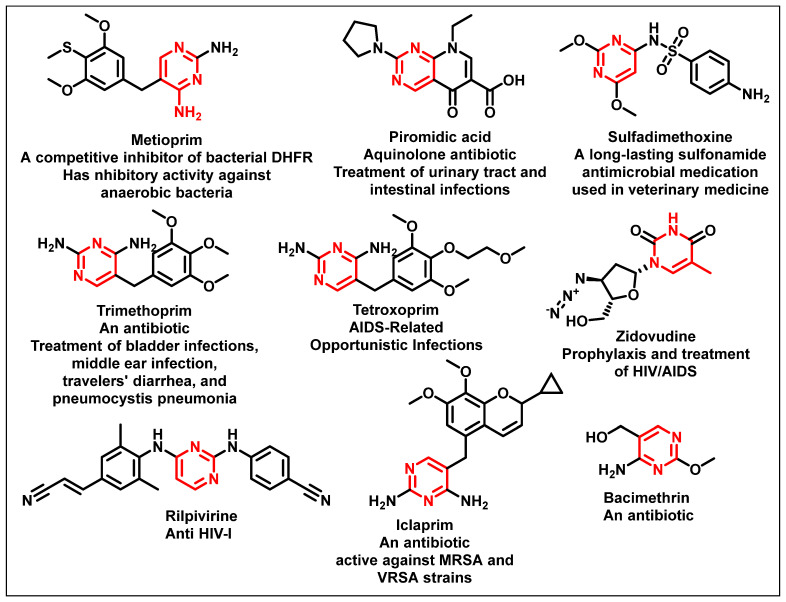
Different examples of FDA-approved pyrimidine-based antimicrobial drugs.

Carbonitrile has significant and diverse biological activities such as anti-allergic, antibacterial, antifungal, anti-HIV, anticonvulsant, and anti-inflammatory activities in addition to β-lactamase inhibition [25,26,27,28]. It is characterized by various properties such as its rigidity, stability in in vivo environments, hydrogen bonding ability, and modest dipole character [28]. Figure 2 represents different examples of FDA-approved pyrimidine-based antimicrobial drugs.

Many medicinal chemists have been working on the hybridization of different substituted benzenesulfonamide rings with other heterocyclic molecules to make new formulations that are more effective and have fewer side effects in order to make sulfonamide or sulfonyl drugs that are more powerful against multiple microbes [29].

In addition to its ability to interact with SH- and NH_2_-containing enzymes and proteins to reveal antimicrobial activity, reports indicate that the thioacetamide molecule also has antifungal activity [30,31].

**Figure 3:**
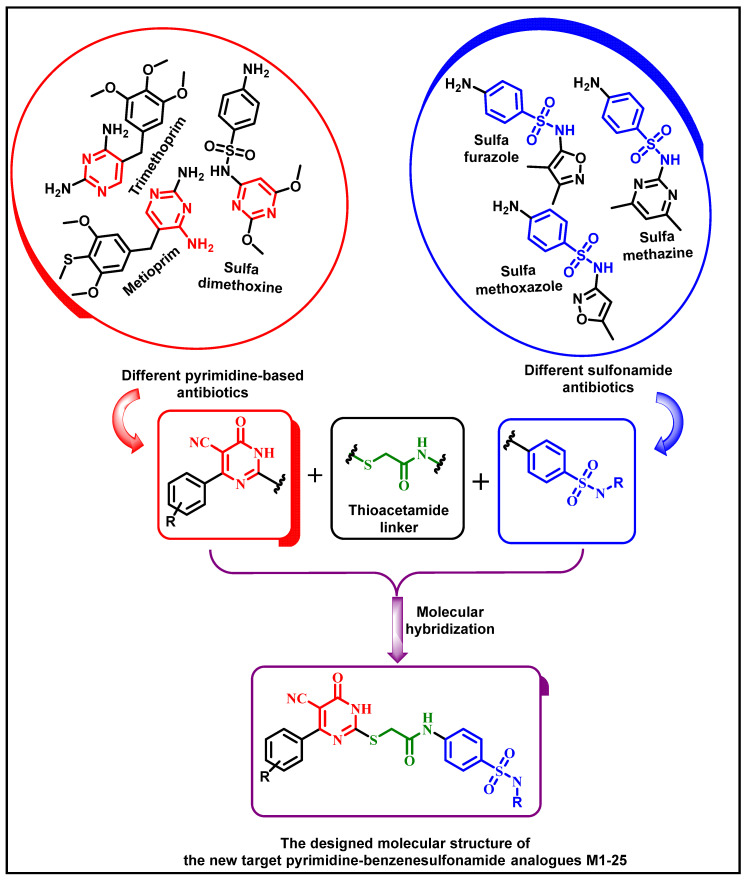
The proposed molecular structures of the thiopyrimidine–benzenesulfonamide compounds **M1–25**.

Considering these factors, we developed a new class of substituted benzenesulfonamide derivatives that are conjugated with various substituted 5-cyano pyrimidine nuclei through a thioacetamide linkage and that potentially exhibit antimicrobial (antibacterial and antifungal) properties. A group of 25 different 2-((5-cyano-6-oxo-4-substitutedphenyl-1,6-dihydropyrimidin-2-yl)thio)-*N*-(4-(*N*-substituted sulfamoyl)phenyl) acetamide derivatives were synthesized in this study (Figure 3). We tested the antimicrobial efficacy of the new target compounds against several human pathogenic microbes (bacteria and fungi). Furthermore, we determined the minimum inhibitory concentration (MIC) and the minimal bactericidal concentration (MBC) values for the most potent analogues, which aided in examining the synergistic effects of the tested compounds. Additionally, we assessed the anti-virulence activity of the latter molecules by evaluating their ability to prevent biofilm formation. Moreover, in silico methodologies to determine their physicochemical parameters were carried out according to Lipinski’s rule of five. Further pharmacokinetic parameters were calculated using the Swiss ADME program.

## 2. Results and Discussion

### 2.1. Chemistry

**Scheme 1:**
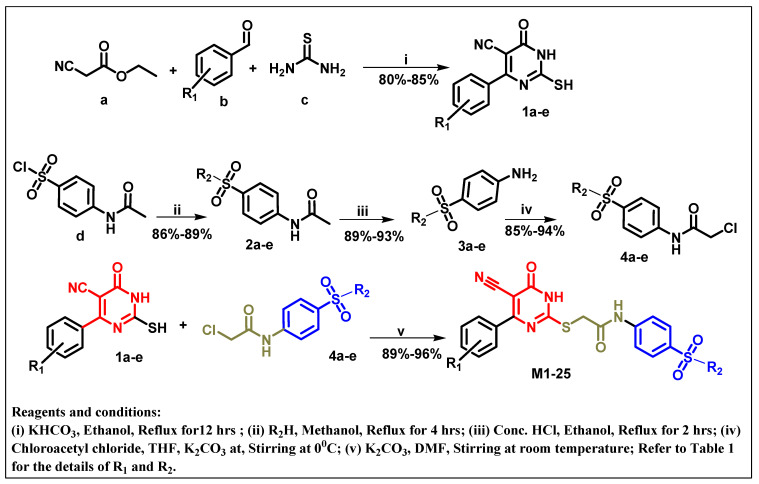
Synthesis of the target thiopyrimidine–benzenesulfonamide analogs **M1–25**.

The synthetic pathway of the target compounds M1–25 is illustrated in Scheme 1. The 6-substituted-4-oxo-2-thioxo-1,2,3,4-tetrahydropyrimidine-5-carbonitriles (**1a–e**) were obtained through a one-pot reaction of thiourea, ethyl cyanoacetate, and the appropriate aldehyde in the presence of K_2_CO_3_, providing quantitative yields as previously reported [32]. The 2-chloro-*N*-(4-(*N*-substitutedsulfamoyl)phenyl)acetamide compounds **4a–e** were synthesized via the acetylation of various substituted 4-amino-*N*-substituted benzenesulfonamide derivatives **3a–e** with chloroacetyl chloride in anhydrous THF at −10 °C under basic conditions [33]. Subsequently, the coupling of the key starting 2-thioxo-1,2,3,4-tetrahydropyrimidine compounds 1 with chloroacetamide compounds 4-1-5 in DMF under basic conditions led to the formation of the target 5-cyano-6-oxo-4-phenyl-1,6-dihydropyrimidine-based analogues M1–25 in excellent yields (Table 1). The structures of the novel compounds were confirmed by their spectral data (^1^H NMR, ^13^CNMR, and mass spectra).

In the ^1^H NMR spectra of the target pyrimidine–benzenesulfonamide analogs M1–25, the amidic NH protons of both the acetamide linker and dihydropyrimidine ring appeared as singlets and very broad singlets downfield in the δ 10.0–13.71 ppm range. The aromatic protons are resolved into four distinct peaks within the range of δ 6.80–8.0 ppm. In addition, the 2H of the thioacetamide group appeared as a singlet signal in the region of δ 3.88–4.30 ppm, while the sulfamoyl aliphatic or hetero-alicyclic protons appeared in the upfield range of δ 1.03–3.15 ppm (for more details, see the Supplementary Materials). The mass spectra showed all of the new target benzenesulfonamide derivative compounds **M1–25** had molecular ion peaks that were in agreement with their expected molecular formulae.

### 2.2. Biological Evaluation

#### 2.2.1. Antimicrobial Activity Determination

The newly synthesized sulfonamide compounds **M1–25** were assessed for their antimicrobial characteristics against the bacterial isolates *E. coli* ATCC-25922*, K. pneumoniae,* and *P. aeruginosa* ATCC 27,853 as Gram-negative bacteria; *S. aureus* ATCC 6538, *S. epidermidis* ATCC 35984, and *B. subtilis* ATCC 6633 as Gram-positive bacteria; and the fungal strain *C. albicans* ATCC-10231.

**Figure 4:**
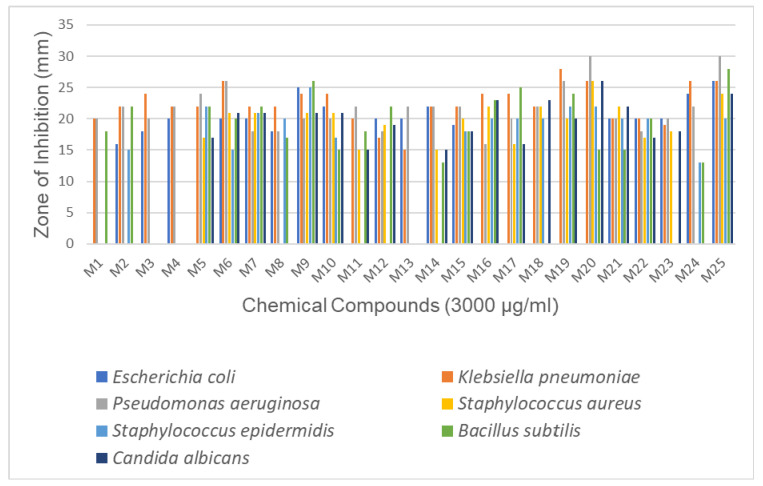
Zone of inhibition (mm) of the new sulfonamide compounds (3000 µg/mL) against various microbial strains.

We selected these specific strains due to their ability to form biofilms and their significant impact on both plant and human health. Utilizing the agar well diffusion process [34], the average diameter of the inhibition zones in millimeters was measured for each tested analogue (3000 µg/mL) against every kind of microbial growth surrounding the discs [34] (Table 2, Figure 4).

The results showed that all of the examined analogues are promising antimicrobial candidates against most of the tested microbial isolates, producing zones of inhibition ranging from 15 to 30 mm. Interestingly, the bacterial strains *K. pneumoniae* and *P. aeruginosa* exhibited outstanding levels of sensitivity towards all of the target compounds (**M1–25**). Both *K. pneumoniae* and *P. aeruginosa* are multidrug-resistant pathogens and are associated with serious hospital-acquired infections such as pneumonia and various sepsis syndromes [35,36,37]. So, these new thiopyrimidine–benzenesulfonamides can be thought of as basic building blocks for the development of new drugs with strong antimicrobial properties that can beat the antibiotic resistance of these two types of organisms.

**Figure 5:**
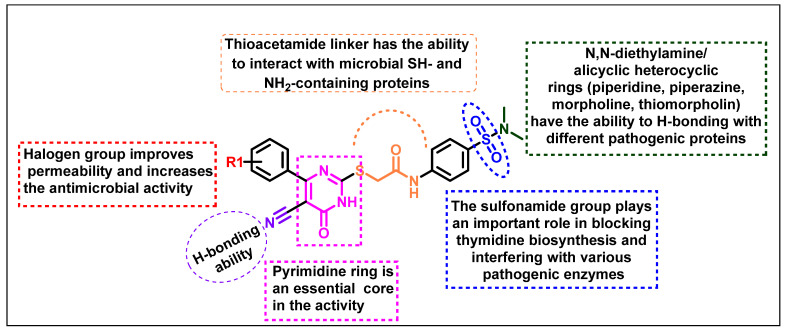
Structure–activity relationship of the new compounds in terms of antimicrobial activity.

Moreover, the halophenyl-5-cyano-6-oxo-1,6-dihydropyrimidin-2-yl-thio-*N*-substituted sulfonyl phenyl acetamide derivatives **6M**, **19M**, **20M**, and **25M** exhibited the most potent wide-spectrum antimicrobial activity against all the examined isolates, producing zones of inhibition (ZOI) ranging from 15 to 30 mm. Halogenation may improve their permeability and enhance the sulfonamide pharmacophore’s antimicrobial activity. The halogenated derivatives may also work against other pathogens that are multidrug-resistant (MDR), such as *S. aureus* and *S. pneumoniae* [38,39] (Figure 5).

#### 2.2.2. MIC and MBC of Selected Compounds against More Susceptible Bacteria

Moreover, minimum inhibitory concentration (MIC (μg/m)) as well as minimum bactericidal concentration (MBC (μg/mL)) assays were performed for the promising compounds **M6**, **M19**, **M20**, and **M25** against *K. pneumonia* and *P. aeruginosa* bacterial strains using the double sequence dilution method [40,41]. The MIC assay represents the lowest concentration of antimicrobial agent that greatly inhibits microbial growth, while the MBC represents the lowest level of antimicrobial agent leading to microbial death. According to the Clinical and Laboratory Standards Institute (CLSI), antibacterial agents are usually evaluated as bactericidal if the MBC is no more than four times the MIC values [42].

The obtained results are summarized in Table 3. It was noticed that the MIC values for the tested compounds were 375 µg/mL against both bacterial strains. On the other hand, the MBC values showed variability among the compounds and bacterial strains. For *Klebsiella pneumoniae*, the MBC values for compounds **M6** and **M19** were 1500 µg/mL, while for compounds **M20** and **M25**, it was 7500 µg/mL. For *Pseudomonas aeruginosa*, the MBC for all four compounds was consistent at 1500 µg/mL. The obtained results confirmed the bactericidal activity of compounds **6M** and **19M** against both *K.* pneumonia and *P. aeruginosa*, with the ratio of MBC/MIC is equal to four. With regard to compounds **M20** and **M25**, their MBC/MIC ratios revealed their bactericidal activity against the *P. aeruginosa* strain and their bacteriostatic impact on *K. pneumoniae.* These data show the newly synthesized sulfonamide derivatives have potential to be developed and optimized as bactericidal agents against some resistant strains.

#### 2.2.3. Determination of the Antibiofilm Effect of the Most Promising Compounds Using TCP Method

A serious public health issue has arisen from infections caused by bacteria with microbial biofilms, as these biofilms have been shown to be thousands of times more resistant to antibacterial drugs compared to their planktonic forms [43]. The persistence of Gram-negative airway pathogens such as *K. pneumoniae* and *P. aeruginosa* and their survival within the lung are mainly attributed to biofilms through the colonization of endotracheal tubes and airways. In addition, these pathogens adapt to the biofilm mode. [43,44,45,46].

**Figure 6:**
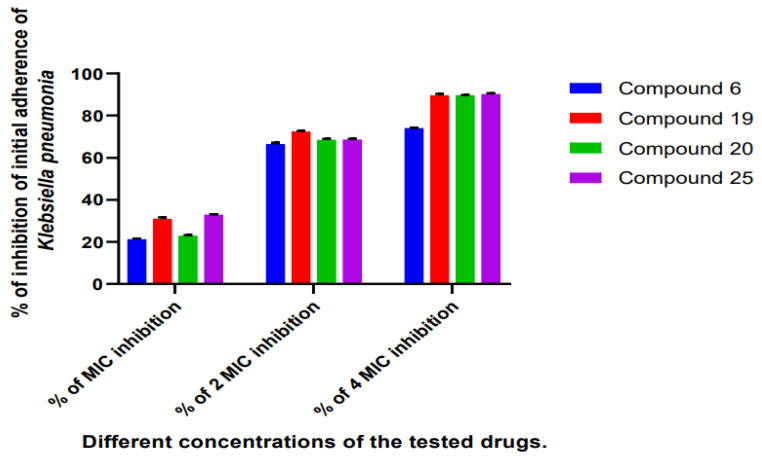
The inhibitory effect of the tested drugs **M6**, **M19**, **M20**, and **M25** on biofilm formation by *K. pneumonia.*

**Figure 7:**
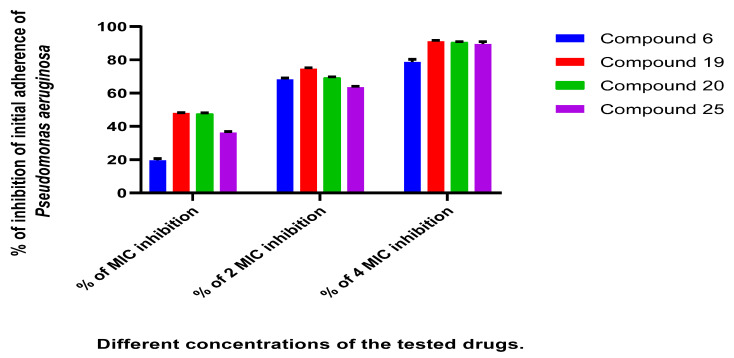
The inhibitory effect of the tested compounds **M6**, **M19**, **M20**, and **M25** on biofilm formation by *P. aeruginosa.*

In our study, the incubation of *K. pneumonia* and *P. aeruginosa* with the most promising four tested compounds, **M6**, **M19**, **M20**, and **M25**, at MIC, 2MIC, and 4MIC concentrations for 24 h showed moderate-to-good levels of biofilm formation prevention, with a percentage of inhibition up to 90% against *K. pneumonia* and 91.8% against *P. aeruginosa* (Figure 6 and Figure 7). These compounds’ inhibition of biofilm formation makes them a promising source of drug leads to control microbial biofilm growth.

### 2.3. Calculated Physicochemical Properties and ADMET (Absorption, Distribution, Metabolism, Excretion, and Toxicity)

The physicochemical properties of the promising candidates **M6**, **M19**, **M20**, and **M25** were assessed and are illustrated in Table 4. All the compounds adhere to Lipinski’s rule. The results showed that the number of H-bond acceptors is <10, the number of H-bond donors is <5, and logP is <5. The compounds displayed a molecular weight approximately greater than 500 D, which is deemed a violation of Lipinski’s rule. Even though the compounds violate one parameter, the drugs still follow Lipinski’s rule. In addition, recently published data have suggested that there is a tendency for orally effective small-molecule inhibitors to slightly exceed 500 D [47]. Table 3 reveals that the majority of the molecules exhibit a slightly elevated total polar surface area (TPSA). In general, these results indicate these promising inhibitors have excellent oral bioavailability and good absorbance. 

We calculated the ADMET properties to gain a deeper understanding of the pharmacokinetic profile of the hit compounds. ADMET prediction is considered an essential study to predict the pharmacokinetic and bioavailability properties of drug-like compounds [48,49,50]. The ADMET results (Table 5) illustrated that the compounds have moderate levels of water solubility and good intestinal absorbance. In addition, the hit compounds showed logKp values < −2.5, which indicates the compounds have reasonable skin permeability. The distribution results of the promising hit compounds showed logBB < −1 and logPS < −3, which indicate the inhibitors are poorly distributed to the brain or CNS.

The metabolism calculations indicate that the inhibitors are metabolized by the CYP3A4 enzyme, while they are not substrates or inhibitors for the CYP2D6 or CYP1A2 enzymes, respectively. The low values of the total clearance of the four molecules reveal that they have good half-lives, and the toxicity study showed no hERG inhibition properties or AMES mutagenicity, which suggests the compounds are not mutagenic or tumorigenic.

**Figure 8:**
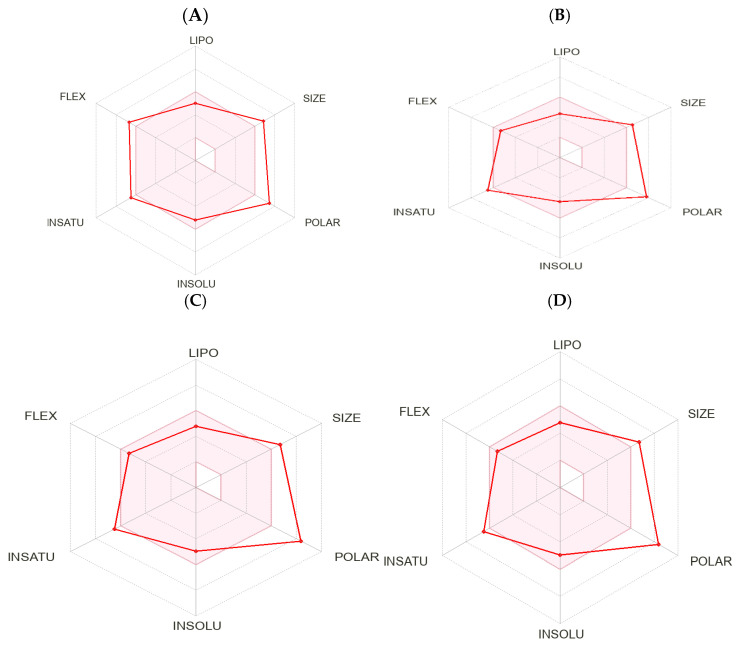
The bioavailability radars of (**A**) compound **M6**, (**B**) compound **M19**, (**C**) compound **M20**, and (**D**) compound **M25**. The pink area represents the optimal range for each property for oral bioavailability. Lipophilicity (LIPO): XLOGP3 between −0.7 and +5.0. Molecular weight (SIZE): MW between 150 and 500 g/mol. Polarity (POLAR): TPSA between 20 and 130 Å2. Solubility (INSOLU): log S not higher than 6. Saturation (INSATU): fraction of carbons in the sp3 hybridization not less than 0.25. Flexibility (FLEX): no more than nine rotatable bonds.

The bioavailability radars of the compounds (Figure 8) showed that the compounds have good pharmacokinetic properties. The pink area of the radars illustrates the properties of lipophilicity, molecular size, solubility, saturation, and flexibility. The compounds are almost within the range of conformity, while the polarity properties are slightly increased over this range. In general, the pharmacokinetic properties of the compounds are promising and can be optimized.

In conclusion, compounds **M6**, **M19**, **M20** and **M25** showed satisfactory ADMET properties. The results indicated good absorption, poor penetration of the blood–brain barrier and the CNS, excellent clearance properties, and no toxicity.

#### Challenges and Future Directions

The failure of a large number of drug candidates in the drug development process due to problems with their pharmacokinetic properties makes in silico ADMET calculations an essential step in drug discovery. Although computational models used for predicting pharmacokinetics have improved significantly, most of them lack sufficient interpretability and sometimes offer poor predictions for novel structures. In this study, we used two different prediction tools to achieve a high level of accuracy. One of the biggest challenges in this study was predicting the absorption and distribution of novel structures. Sometimes, the prediction results show differences from in vivo excremental results. This is due to the complexity of the mathematical calculations used in prediction tools to simulate intestinal absorption or blood–brain distribution. To achieve this, further in vivo studies are required to measure the human intestinal absorption and blood–brain permeability of promising compounds.

## 3. Experimental Section

### 3.1. Chemistry

All reagents and solvents were sourced from commercial suppliers, including Sigma Aldrich (St. Louis, MI, USA) and Fisher Scientific (Hampton, VI, USA).and used without purification unless stated otherwise. The instruments used to determine melting points, spectral data (^1^H NMR, ^19^F NMR ^13^C NMR, and mass), and chemical analyses are included along with detailed descriptions in a file in the Supplementary Materials.

#### 3.1.1. General Procedure for Preparation of 6-Substituted-4-Oxo-2-Thioxo-1,2,3,4-Tetrahydropyrimidine-5-Carbonitriles [32]

A mixture of thiourea (1.839 g, 24 mmol), a suitable aldehyde (24 mmol), ethylcyanoacetate (2.734 g, 24 mmol), and K_2_CO_3_ (4.837 g, 24 mmol) was added to a round-bottomed flask, followed by ethanol (50 mL). The reaction mixture was then heated under reflux for 12 h and monitored by TLC. The resulting creamy precipitate was filtered, washed with ethanol, and vacuum-dried. The product was then dissolved in the minimum amount of hot water (100 mL) and acidified with glacial acetic acid to pH of 4. The white precipitate was suction-filtered and then recrystallized from aqueous DMF.

##### 2-Mercapto-6-Oxo-4-Phenyl-1,6-Dihydropyrimidine-5-Carbonitrile (**1a**)


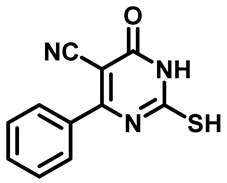


White powder, 81%. mp 255 °C. ^1^H NMR (400 MHz, DMSO-*d*_6_) δ 7.54–7.60 (m, 2H), 7.62–7.71 (m, 3H), 13.20 (s, 1H), 13.34 (bs, 1H). ^13^CNMR (101 MHz, DMSO-*d*_6_)) δ 91.3, 115.2, 128.9, 129.2, 129.8, 132.6, 159.0, 161.4, 176.7.

##### 4-(4-Bromophenyl)-2-Mercapto-6-Oxo-1,6-Dihydropyrimidine-5-Carbonitrile (**1b**)


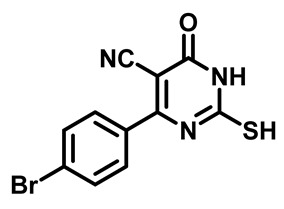


White powder, 85%. mp 284 °C. ^1^H NMR (400 MHz, DMSO-*d*_6_) δ 7.63 (d, J = 8.6 Hz, 2H), 7.80 (d, J = 8.5 Hz, 2H), 13.18 (s, 1H), 13.35 (bs, 1H). ^13^C NMR (101 MHz, DMSO-*d*_6_) δ 91.5, 115.0, 126.3, 129.0, 131.3, 132.0, 158.8, 160.4, 176.6.

##### 4-(4-Fluorophenyl)-2-Mercapto-6-Oxo-1,6-Dihydropyrimidine-5-Carbonitrile (**1c**)


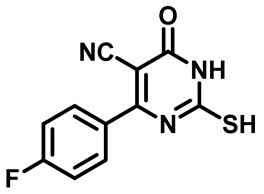


White powder, 84%. mp 270–272 °C, ^1^H NMR (400 MHz, DMSO-*d*_6_) δ 7.44 (t, *J* = 8.9 Hz, 2H), 7.76 (dd, *J* = 8.7, 5.4 Hz, 2H), 13.22 (s, 1H), 13.36 (bs, 1H).^13^CNMR (101 MHz, DMSO-*d*_6_) δ 91.4, 115.2, 116.0, 116.3, 126.2, 126.2, 132.1, 132.2, 158.9, 160.5, 163.4, 165.8, 176.6.

##### 4-(4-Chlorophenyl)-2-Mercapto-6-Oxo-1,6-Dihydropyrimidine-5-Carbonitrile (**1d**)


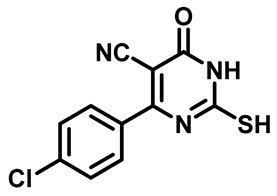


White powder, 86%. mp 252 °C, ^1^H NMR (400 MHz, DMSO-*d*_6_) δ 7.64–7.73 (m, 4H), 13.22 (s, 1H), 13.38 (bs, 1H). ^13^CNMR (101 MHz, DMSO-*d*_6_) δ 91.5, 115.1, 128.6, 129.1, 131.2, 137.4, 158.9, 160.4, 176.6.

##### 4-(3-Chlorophenyl)-2-Mercapto-6-Oxo-1,6-Dihydropyrimidine-5-Carbonitrile (**1e**)


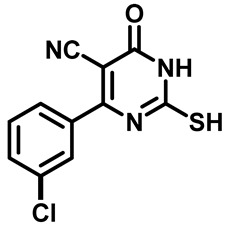


Yellow powder, 80%. mp 229 °C, ^1^HNMR (400 MHz, DMSO-*d*_6_) δ 7.62 (d, *J* = 7.7 Hz, 1H), 7.64–7.68 (m, 1H), 7.72 (dt, *J* = 7.7, 1.8 Hz, 1H), 7.78 (t, *J* = 1.9 Hz, 1H), 13.24 (s, 1H), 13.40 (bs, 1H). ^13^CNMR (101 MHz, DMSO-*d*_6_) δ 91.7, 114.9, 128.0, 129.1, 130.9, 131.7, 132.3, 133.4, 158.8, 160.0, 176.6.

#### 3.1.2. General Procedure for Preparation of N-Substituted Sulfonyl Phenyl Acetamides **2**

To a solution of 4-acetylaminobenzenesulfonyl chloride (2.34 g, 10 mmol) in methanol, 20 mL of appropriate amine (20 mmol) was added dropwise at room temperature. The reaction mixture was refluxed for 4 h. After cooling, the solvent was removed under pressure, and water (50 mL) was added. The mixture was stirred at room temperature for 30 min. The solid obtained was filtered, washed with cold water, and dried. The compound formed was then recrystallized with 50% ethanol.

##### *N*-(4-(N,N-Diethylsulfamoyl)Phenyl)Acetamide (**2a**)


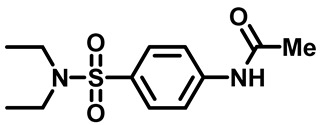


White powder, 2.30 g, 85%, ^1^H NMR (400 MHz, DMSO-*d*_6_) δ 1.03 (t, J = 7.1 Hz, 6H), 2.09 (s, 3H), 3.13 (q, J = 7.1 Hz, 4H), 7.71 (d, J = 8.9 Hz, 2H), 7.78 (d, J = 8.7 Hz, 2H), 10.30 (s, 1H). ^13^CNMR (101 MHz, DMSO-*d*_6_) δ 14.5, 24.6, 42.2, 119.2, 128.3, 133.9, 143.4, 169.5.

##### *N*-(4-(Piperidin-1-Ylsulfonyl)Phenyl)Acetamide (**2b**)


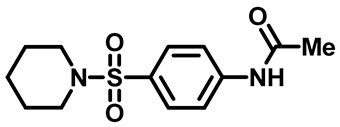


White powder, 2.42 g, 86%, ^1^H NMR (400 MHz, DMSO-*d*_6_) δ 1.36 (m, 2H), 1.54 (p, J = 5.8 Hz, 4H), 2.10 (s, 3H), 2.86 (t, J = 5.5 Hz, 4H), 7.66 (d, J = 8.8 Hz, 2H), 7.81 (d, J = 8.8 Hz, 2H), 10.33 (s, 1H). ^13^CNMR (101 MHz, DMSO-*d*_6,_): δ 23.4, 24.6, 25.1, 47.0, 119.1, 129.1, 129.5, 143.8, 169.5.

##### *N*-(4-((4-Methylpiperazin-1-Yl)Sulfonyl)Phenyl)Acetamide (**2c**)


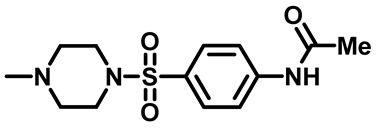


White powder, 2.51 g, 84%. ^1^H NMR (400 MHz, DMSO-*d*_6_) δ 2.10 (s, 3H), 2.14 (s, 3H), 2.36 (t, J = 4.9 Hz, 4H), 2.87 (t, J = 5.0 Hz, 4H), 7.66 (d, J = 8.8 Hz, 2H), 7.83 (d, J = 8.8 Hz, 2H), 10.36 (s, 1H)^13^C NMR (101 MHz, DMSO-*d*_6_) δ 24.6, 45.7, 46.2, 54.0, 119.1, 128.9, 129.2, 144.0, 169.5.

##### *N*-(4-(Morpholinosulfonyl)Phenyl)Acetamide (**2d**)


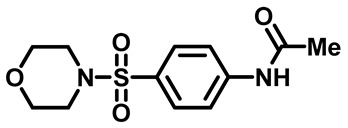


White powder, 2.39 g, 84%. ^1^H NMR (400 MHz, DMSO-*d*_6_) δ 2.11 (s, 3H), 2.84 (t, 4H), 3.62 (t, J = 4.7 Hz, 4H), 7.67 (d, J = 8.7 Hz, 2H), 7.84 (d, J = 8.8 Hz, 2H), 10.38 (s, 1H). ^13^CNMR (101 MHz, DMSO-*d*_6_) δ 24.6, 46.4, 65.7, 119.2, 128.4, 129.4, 144.1, 169.6.

##### *N*-(4-(Thiomorpholinosulfonyl)Phenyl)Acetamide (**2e**)


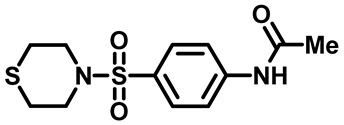


White powder, 2.67 g, 89%. ^1^HNMR (400 MHz, DMSO-*d*_6_) δ 2.10 (s, 3H), 2.66 (t, 4H), 3.18 (t, 4H), 7.68 (d, J = 8.8 Hz, 2H), 7.83 (d, J = 8.9 Hz, 2H), 10.37 (s, 1H). ^13^CNMR (101 MHz, DMSO-*d*_6_) δ 24.6, 26.9, 48.2, 119.2, 128.9, 130.1, 144.0, 169.6.

#### 3.1.3. General Procedure for Preparation of 4-Amino-Benzenesulfonamides **3**

A substituted sulfonyl phenyl acetamide 2 (10 mmol) was added to concentrated HCl (10 mL) and methanol (10 mL) and heated to 80–90 °C for two hours. The solution became clear upon heating. After two hours, the solution was concentrated using a rotary evaporator. The resulting solution was then neutralized to pH of 7 by the addition of a saturated aqueous solution of Na_2_CO_3_. The product was washed with cold water and recrystallized with ethanol.

##### 4-Amino-N,N-Diethylbenzenesulfonamide (**3a**)


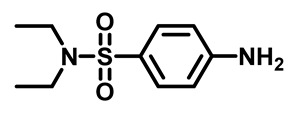


White powder, 2.10 g, 92%. ^1^H NMR (400 MHz, DMSO-*d*_6_) δ 1.02 (t, J = 7.1 Hz, 6H), 3.07 (q, J = 7.1 Hz, 4H), 5.93 (s, 2H), 6.62 (d, J = 8.7 Hz, 2H), 7.39 (d, J = 8.7 Hz, 2H). ^13^CNMR (101 MHz, DMSO-*d*_6_) δ 14.5, 42.0, 113.3, 125.1, 129.1, 153.1.

##### 4-(Piperidin-1-Ylsulfonyl)Aniline (**3b**)


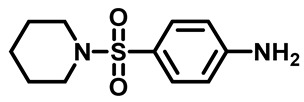


White powder, 2.16 g, 90%. ^1^H NMR (400 MHz, DMSO-*d*_6_) δ 1.18–1.45 (m, 2H), 1.53 (p, J = 5.7 Hz, 4H), 2.79 (t, J = 5.4 Hz, 4H), 6.00 (s, 2H), 6.65 (d, J = 8.7 Hz, 2H), 7.34 (d, J = 8.7 Hz, 2H). ^13^CNMR (101 MHz, DMSO-*d*_6_) δ 23.5, 25.2, 47.0, 113.2, 120.7, 129.9, 153.5.

##### 4-((4-Methylpiperazin-1-Yl)Sulfonyl)Aniline (**3c**)


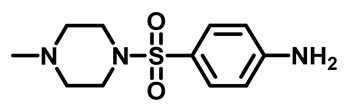


White powder, 2.28 mg, 89%. ^1^HNMR (400 MHz, DMSO-*d*_6_) δ 2.13 (s, 3H), 2.34 (t, J = 4.9 Hz, 4H), 2.80 (t, J = 4.9 Hz, 4H), 6.05 (s, 2H), 6.66 (d, J = 8.7 Hz, 2H), 7.35 (d, J = 8.7 Hz, 2H). ^13^CNMR (101 MHz, DMSO-*d*_6_) δ 45.8, 46.1, 54.0, 113.2, 119.9, 130.0, 153.7.

##### 4-(Morpholinosulfonyl)Aniline (**3d**)


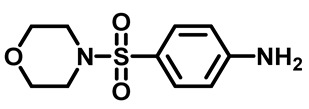


White powder, 2.23 g, 92%. ^1^H NMR (400 MHz, DMSO-*d*_6_) δ 2.78 (t, 4H), 3.61 (t, 4H), 6.09 (s, 2H), 6.67 (d, J = 8.7 Hz, 2H), 7.36 (d, J = 8.7 Hz, 2H). ^13^CNMR (101 MHz, DMSO-*d*_6_) δ 46.4, 65.8, 113.2, 119.3, 130.2, 153.8.

##### 4-(Thiomorpholinosulfonyl)Aniline (**3e**)


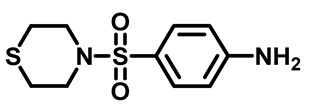


White powder, 2.41 g, 93%. ^1^H NMR (400 MHz, DMSO-*d*_6_) δ 2.65 (t, 4H), 3.11 (t, 4H), 6.06 (s, 2H), 6.66 (d, J = 8.7 Hz, 2H), 7.36 (d, J = 8.7 Hz, 2H). ^13^CNMR (101 MHz, DMSO) δ 26.9, 48.2, 113.3, 121.0, 129.7, 153.7.

#### 3.1.4. General Procedure for Preparation of *N*-Substituted Sulfonyl Phenyl Chloro-Acetamides [33]

In a flame-dried flask, chloroacetyl chloride (0.631 mg, 5.5 mmol) was added dropwise at 0 °C to a solution of sulfonylaniline 3 (1.00 g, 4.3 mmol) in anhydrous THF (30 mL) containing K_2_CO_3_ (1.18 g, 8.6 mmol). The reaction mixture was stirred for 4 h and monitored by TLC. After reaction was complete, water (60 mL) was added, and the aqueous layer was extracted with ethyl acetate. The organic layer was dried over anhydrous Na_2_SO_4_, and the solvent was evaporated to yield compound 4. The crude product was used for the next step without further purification.

##### 2-Chloro-*N*-(4-(*N*,*N*-Diethylsulfamoyl)Phenyl)Acetamide (**4a**)


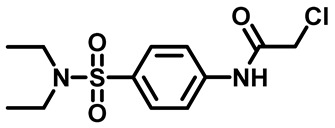


White powder, 88%, ^1^H NMR (400 MHz, CDCl_3_) δ 1.05 (t, J = 7.2 Hz, 6H), 3.16 (q, J = 7.1 Hz, 4H), 4.15 (s, 2H), 7.59–7.74 (m, 4H), 8.91 (s, 1H). ^13^CNMR (101 MHz, CDCl_3_) δ 14.1, 42.1, 43.1, 120.0, 128.0, 135.6, 141.0, 165.1.

##### 2-Chloro-*N*-(4-(Piperidin-1-Ylsulfonyl)Phenyl)Acetamide (**4b**)


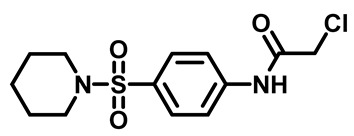


White powder, 91%. ^1^H NMR (400 MHz, DMSO-*d*_6_)δ 1.30–1.38 (m, 2H), 1.53 (t, J = 5.6 Hz, 4H), 2.86 (t, J = 5.4 Hz, 4H), 4.31 (s, 2H), 7.70 (d, J = 8.8 Hz, 2H), 7.83 (d, J = 8.8 Hz, 2H), 10.70 (s, 1H). ^13^C NMR (101 MHz, DMSO-*d*_6_) δ 23.3, 25.1, 44.0, 47.0, 119.6, 129.2, 130.5, 142.9, 165.8.

##### 2-Chloro-*N*-(4-((4-Methylpiperazin-1-yl)Sulfonyl)Phenyl)Acetamide (**4c**)


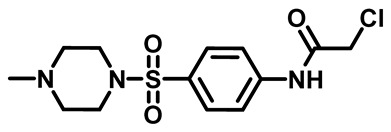


White powder, 94%. ^1^HNMR (400 MHz, DMSO-*d*_6_) δ 2.13 (s, 3H), 2.35 (t, J = 5.0 Hz, 4H), 2.87 (t, J = 4.9 Hz, 4H), 4.32 (s, 2H), 7.71 (d, J = 8.8 Hz, 2H), 7.85 (d, J = 8.8 Hz, 2H), 10.79 (s, 1H).^13^CNMR (101 MHz, DMSO-*d*_6_) δ 44.0, 45.7, 46.2, 53.9, 119.7, 129.3, 129.8, 143.1, 165.9.

##### 2-Chloro-*N*-(4-(Morpholinosulfonyl)Phenyl)Acetamide (**4d**)


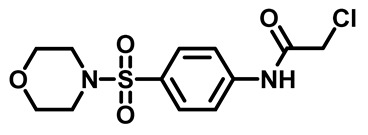


White powder, 89%, ^1^H NMR (400 MHz, CDCl_3_) δ 3.02 (t, 4H), 3.75 (t, 4H), 4.24 (s, 2H), 7.73–7.81 (m, 4H), 8.48 (s, 1H). ^13^CNMR (101 MHz, CDCl_3_) δ 42.8, 46.0, 66.1, 119.8, 129.2, 131.1, 141.1, 164.3.

##### 2-Chloro-*N*-(4-(Thiomorpholinosulfonyl)Phenyl)Acetamide (**4e**)


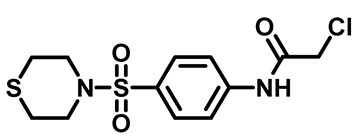


White powder, 85%. ^1^H NMR (400 MHz, DMSO-*d*_6_) δ 2.66 (q, J = 4.8, 4.2 Hz, 4H), 3.20 (q, J = 4.8, 4.2 Hz, 4H), 4.32 (d, J = 3.4 Hz, 2H), 7.72 (d, J = 8.7 Hz, 2H), 7.85 (d, J = 8.7 Hz, 2H), 10.73 (s, 1H). ^13^C NMR (101 MHz, DMSO) δ 26.9, 44.0, 48.2, 119.8, 129.0, 131.0, 143.1, 165.9.

#### 3.1.5. General Procedure for Preparation of Final Target Compounds **M1**–**25**

In an oven-dried flask, a mixture of compound 1 (1.30 mmole) and K_2_CO_3_ (1.43 mmole) in 10 mL DMF was stirred for 20 min under nitrogen. Then, an appropriate amount of sulfonyl acetamide compound 4 (1.30 mmole) was added to the flask. The reaction mixture was stirred for 20–34 h under nitrogen at room temperature and monitored by TLC. Upon completion of the reaction, water (60 mL) was added, and the mixture was acidified with glacial acetic acid. Precipitation formed, which was then filtered, washed with cold water, and purified by flash column chromatography.

##### 2-((5-Cyano-6-Oxo-4-Phenyl-1,6-Dihydropyrimidin-2-yl)thio)-N-(4-(N,N-Diethylsulfamoyl)Phenyl)Acetamide (**M1**)


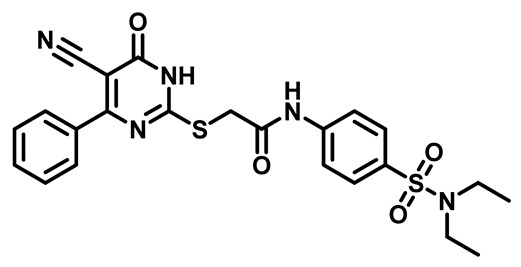


White powder, mp 207–210, 601 mg, 93%. ^1^H NMR (400 MHz, DMSO-*d*_6_) δ 1.03 (t, J = 7.1 Hz, 6H), 3.15 (q, J = 7.1 Hz, 4H), 4.20 (s, 2H), 7.29 (t, J = 7.7 Hz, 2H), 7.50 (t, J = 7.5 Hz, 1H), 7.70–7.80 (m, 4H), 7.82 (d, J = 7.8 Hz, 2H), 10.78 (s, 1H). ^13^CNMR (101 MHz, DMSO-*d*_6_) δ 14.4, 36.1, 42.1, 93.5, 116.3, 119.3, 128.4, 128.8, 129.2, 132.0, 134.3, 135.5, 143.1, 161.8, 166.3, 166.5, 167.5. Calculated MS: 497. 1191; experimental MS (ESI) *m*/*z* (%): 498.1450 [M + H].

##### 2-((5-Cyano-6-Oxo-4-Phenyl-1,6-Dihydropyrimidin-2-yl)Thio)-N-(4-(Piperidin-1-Ylsulfonyl)Phenyl)Acetamide (**M2**)


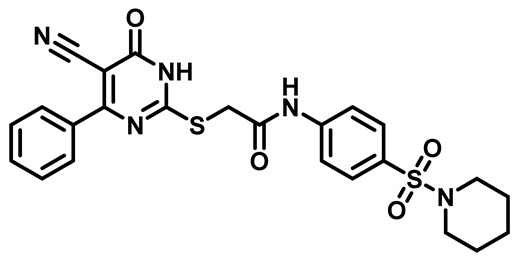


White powder, mp 184–186 629 mg, 95%. ^1^H NMR (400 MHz, DMSO-*d*_6_) δ 1.35 (p, 2H), 1.54 (t, J = 5.8 Hz, 4H), 2.86 (t, J = 5.5 Hz, 4H), 4.22 (s, 2H), 7.29 (t, J = 7.7 Hz, 2H), 7.49 (t, J = 7.5 Hz, 1H), 7.69 (d, J = 8.7 Hz, 2H), 7.76–7.86 (m, 4H), 10.76 (s, 1H), 13.71 (bs, 1H). ^13^CNMR (101 MHz, DMSO-*d*_6_) δ 23.4, 25.1, 36.2, 47.1, 93.7, 116.2, 119.2, 128.8, 129.2, 129.2, 130.0, 132.0, 135.5, 143.4, 161.6, 166.2, 166.5, 167.5. Calculated MS: 509.1191; experimental MS (ESI) *m*/*z* (%): 509.1640 [M + H].

##### 2-((5-Cyano-6-Oxo-4-Phenyl-1,6-Dihydropyrimidin-2-yl)Thio)-N-(4-((4-Methylpiperazin-1-yl)Sulfonyl)Phenyl)Acetamide (**M3**)


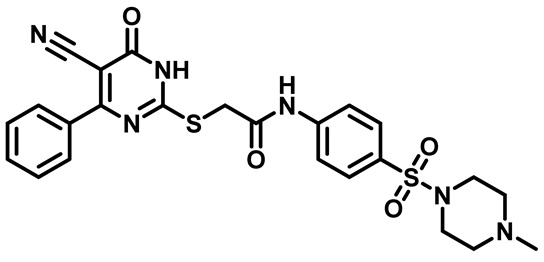


White powder, mp 213–215 °C, 641 mg, 94%, ^1^H NMR (400 MHz, DMSO-*d*_6_) δ 2.67 (s, 3H), 3.16 (s, 8H), 4.10 (s, 2H), 7.37 (t, J = 7.8 Hz, 2H), 7.51 (t, J = 7.4 Hz, 1H), 7.73 (d, J = 8.8 Hz, 2H), 7.81 (d, J = 7.7 Hz, 2H), 7.85 (d, J = 8.4 Hz, 2H), 11.15 (s, 1H). ^13^CNMR (101 MHz, DMSO) δ 36.0, 42.9, 43.9, 52.4, 92.1, 117.8, 119.4, 128.5, 128.7, 129.0, 129.5, 131.5, 136.4, 144.1, 167.6, 167.7. Calculated MS: 524.1300; experimental MS (ESI) *m*/*z* (%): 525.1303 [M + H].

##### 2-((5-Cyano-6-Oxo-4-Phenyl-1,6-Dihydropyrimidin-2-yl)Thio)-N-(4-(Morpholinosulfonyl)Phenyl)Acetamide (**M4**)


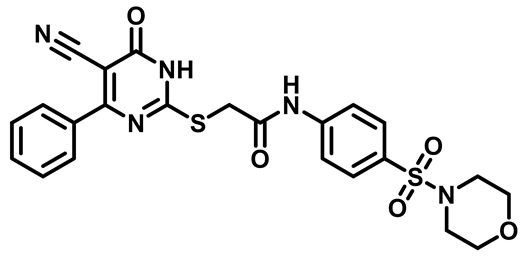


White powder, mp 205–208 °C, 598 mg, 90%, ^1^HNMR (400 MHz, DMSO-*d*_6_) δ 2.85 (t, J = 4.7, 4.1 Hz, 4H), 3.62 (t, J = 4.7 Hz, 4H), 3.88 (s, 2H), 7.39 (t, J = 7.3 Hz, 2H), 7.46 (t, J = 7.3 Hz, 1H), 7.67 (d, J = 8.8 Hz, 2H), 7.76 (d, J = 7.0 Hz, 2H), 7.82 (d, J = 8.8 Hz, 2H), 11.42 (s, 1H).^13^CNMR (101 MHz, DMSO-*d*_6_) δ 35.8, 46.4, 65.8, 90.2, 119.1, 120.0, 128.5, 128.7, 129.4, 130.3, 137.8, 143.9, 167.7, 168.9. Calculated MS: 511.0984; experimental MS (ESI) *m*/*z* (%): 512.5891 [M + H].

##### 2-((5-Cyano-6-Oxo-4-Phenyl-1,6-Dihydropyrimidin-2-yl)thio)-N-(4-(Thiomorpholinosulfonyl)Phenyl)Acetamide (**M5**)


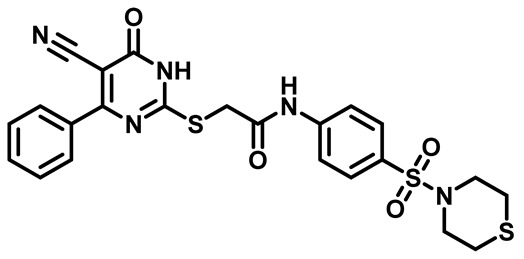


White powder, mp 216–219 °C, 610, 89%. ^1^H NMR (400 MHz, DMSO-*d*_6_) δ 2.66 (t, J = 4.7 Hz, 4H), 3.19 (t, J = 4.7 Hz, 4H), 3.88 (s, 2H), 7.39 (t, J = 7.5 Hz, 2H), 7.46 (t, J = 7.3 Hz, 1H), 7.68 (d, J = 8.7 Hz, 2H), 7.75 (d, J = 7.5 Hz, 2H), 7.81 (d, J = 8.7 Hz, 2H), 11.38 (s, 1H). ^13^CNMR (101 MHz, DMSO-*d*_6_) δ 26.9, 35.8, 48.2, 90.2, 119.2, 120.0, 128.5, 128.7, 129.0, 130.2, 130.3, 137.8, 143.8, 161.5 167.7, 168.9, 171.7. Calculated MS: 527.0756; experimental MS (ESI) *m*/*z* (%): 528.6730 [M + H].

##### 2-((4-(4-Bromophenyl)-5-Cyano-6-Oxo-1,6-Dihydropyrimidin-2-yl)Thio)-N-(4-(N,N-Diethylsulfamoyl)Phenyl)Acetamide (**M6**)


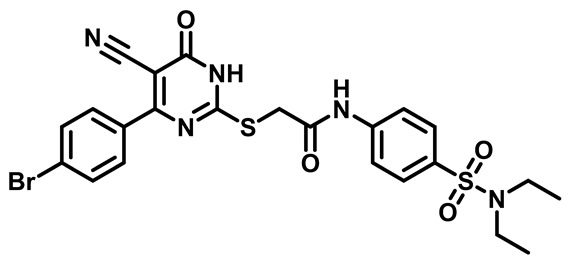


White powder, mp 242–245 °C, 696 mg, 93%. ^1^H NMR (400 MHz, DMSO-*d*_6_) δ 1.03 (t, J = 7.1 Hz, 6H), 3.15 (q, J = 7.1 Hz, 4H), 4.19 (s, 2H), 7.46 (d, J = 8.4 Hz, 2H), 7.73–7.78 (m, 6H), 10.70 (s, 1H). ^13^CNMR (101 MHz, DMSO-*d*_6_) δ 14.4, 36.1, 42.2, 93.8, 116.0, 119.3, 125.9, 128.4, 131.1, 131.8, 134.3, 134.6, 142.9, 161.3, 166.4, 166.4, 166. 5. Calculated MS: 575.0297; experimental MS (ESI) *m*/*z* (%):574.0240 [M − H].

##### 2-((4-(4-Bromophenyl)-5-Cyano-6-Oxo-1,6-Dihydropyrimidin-2-yl)thio)-N-(4-(Piperidin-1-Ylsulfonyl)Phenyl)Acetamide (**M7**)


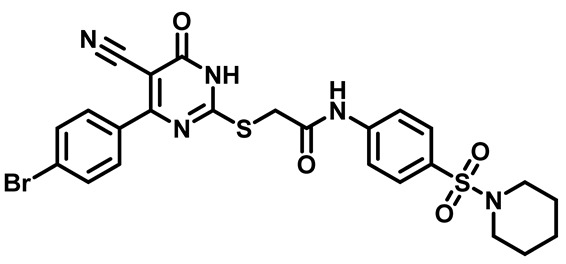


White powder, mp 257–260 °C, 734 mg, 96%. ^1^H NMR (400 MHz, DMSO-*d*_6_) δ 1.35 (p, J = 5.4, 4.7 Hz, 2H), 1.54 (t, J = 5.7 Hz, 4H), 2.86 (t, J = 5.4 Hz, 4H), 4.19 (s, 2H), 7.45 (d, J = 8.4 Hz, 2H), 7.68 (d, J = 8.6 Hz, 2H), 7.75 (d, J = 8.5 Hz, 2H), 7.79 (d, J = 8.7 Hz, 2H), 10.74 (s, 1H). ^13^CNMR (101 MHz, DMSO-*d*_6_) δ 23.4, 25.1, 36.1, 47.1, 93.8, 116.0, 119.2, 125.9, 129.1, 130.0, 131.2, 131.8, 134.6, 143.2, 161.3, 166.4, 166.5, 166.5. Calculated MS: 587.0297; experimental MS (ESI) *m*/*z* (%): 588.0206 [M + H].

##### 2-((4-(4-Bromophenyl)-5-Cyano-6-Oxo-1,6-Dihydropyrimidin-2-yl)thio)-N-(4-((4-Methylpiperazin-1-yl)Sulfonyl)Phenyl)Acetamide (**M8**)


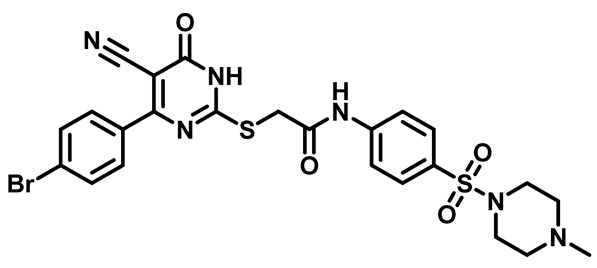


White powder, mp 230–233 °C, 745 mg, 95%, ^1^H NMR (400 MHz, DMSO-*d*_6_) δ 2.64 (s, 3H), 3.09 (s, 8H), 3.99 (s, 2H), 7.60 (d, J = 8.6 Hz, 2H), 7.72 (d, J = 4.0 Hz, 2H), 7.74 (d, J = 3.8 Hz, 2H), 7.86 (d, J = 8.8 Hz, 2H), 11.21 (s, 1H). ^13^CNMR (101 MHz, DMSO-*d*_6_) δ 35.9, 43.3, 44.2, 52.6, 91.1, 118.7, 119.4, 124.5, 128.3, 129.5, 130.8, 131.7, 136.3, 144.1, 166.5, 168.2. Calculated MS: 602.0406; experimental MS (ESI) *m*/*z* (%): 603.0432 [M + H].

##### 2-((4-(4-Bromophenyl)-5-Cyano-6-oxo-1,6-Dihydropyrimidin-2-yl)thio)-N-(4-(Morpholinosulfonyl)Phenyl)Acetamide (**M9**)


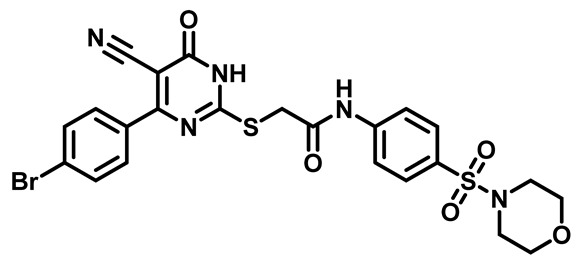


White powder, mp 218–222 °C, 698 mg, 91%, ^1^H NMR (400 MHz, DMSO-*d*_6_) δ 2.86 (t, J = 4.7 Hz, 4H), 3.64 (t, J = 4.8 Hz, 4H), 4.21 (s, 2H), 7.48 (d, J = 8.5 Hz, 2H), 7.70 (d, J = 8.6 Hz, 2H), 7.77 (d, J = 8.4 Hz, 2H), 7.82 (d, J = 8.6 Hz, 2H), 10.77 (s, 1H). ^13^CNMR (101 MHz, DMSO-*d*_6_) δ 36.2, 46.4, 65.8, 93.8, 116.1, 119.3, 125.9, 128.9, 129.5, 131.1, 131.8, 134.7, 143.6, 166.5, 166.6. Calculated MS: 589.0089; experimental MS (ESI) *m*/*z* (%): 590.0132 [M + H].

##### 2-((4-(4-Bromophenyl)-5-Cyano-6-Oxo-1,6-Dihydropyrimidin-2-yl)Thio)-N-(4-(Thiomorpholinosulfonyl)Phenyl)Acetamide (**M10**)


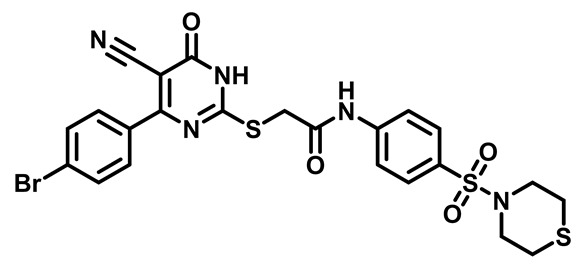


White powder, mp 224–226 °C, 693 mg, 88%, ^1^H NMR (400 MHz, DMSO-*d*_6_) δ 2.68 (t, J = 4.9 Hz, 4H), 3.20 (t, J = 5.0 Hz, 4H), 4.20 (s, 2H), 7.49 (d, J = 8.5 Hz, 2H), 7.71 (d, J = 8.6 Hz, 2H), 7.76 (d, J = 8.4 Hz, 2H), 7.81 (d, J = 8.6 Hz, 2H), 10.77 (s, 1H). ^13^C NMR (101 MHz, DMSO) δ 26.9, 48.3, 93.8, 116.0, 119.3, 125.9, 129.0, 130.5, 131.1, 131.8, 134.6, 143.5, 161.3, 166.4, 166.5, 166.6. Calculated MS: 604.9861; experimental MS (ESI) *m*/*z* (%): 605.9912 [M + H].

##### 2-((4-(4-Chlorophenyl)-5-Cyano-6-Oxo-1,6-Dihydropyrimidin-2-yl)thio)-N-(4-(N,N-Diethylsulfamoyl)Phenyl)Acetamide (**M11**)


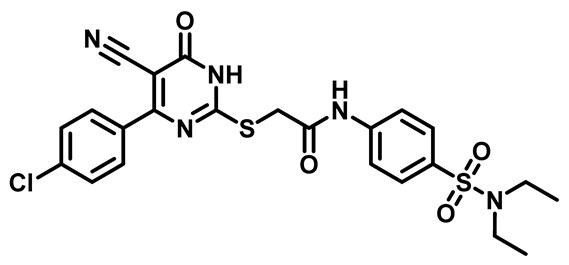


White powder, mp 230–233. ^º^C, 650 mg, 94%, ^1^H NMR (400 MHz, DMSO-*d*_6_) δ 1.02 (t, J = 7.1 Hz, 7H), 3.14 (q, J = 7.1 Hz, 4H), 4.18 (s, 2H), 7.32 (d, J = 8.5 Hz, 2H), 7.72–7.77 (m, 4H), 7.83 (d, J = 8.5 Hz, 2H), 10.72 (s, 1H). ^13^CNMR (101 MHz, DMSO-*d*_6_) δ 14.4, 36.2, 42.2, 93.8, 116.0, 119.3, 128.5, 128.8, 131.0, 134.3, 134.3, 137.0, 142.9, 161.4, 166.3, 166.4, 166.4. Calculated MS: 531.0802; experimental MS (ESI) *m*/*z* (%): 532.0280 [M + H].

##### 2-((4-(4-Chlorophenyl)-5-Cyano-6-Oxo-1,6-Dihydropyrimidin-2-yl)Thio)-N-(4-(Piperidin-1-ylsulfonyl)Phenyl)Acetamide (**M12**)


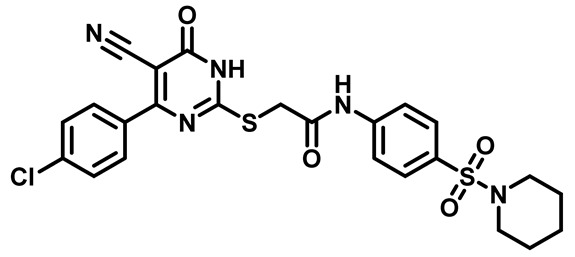


White powder, mp 238–240 °C, 671 mg,95%. ^1^H NMR (400 MHz, DMSO-*d*_6_) δ 1.35 (p, J = 6.0 Hz, 2H), 1.53 (p, J = 5.7 Hz, 4H), 2.85 (t, J = 5.4 Hz, 4H), 4.19 (s, 2H), 7.31 (d, J = 8.4 Hz, 2H), 7.68 (d, J = 8.6 Hz, 2H), 7.79 (d, J = 8.7 Hz, 2H), 7.83 (d, J = 8.5 Hz, 2H), 10.75 (s, 1H). ^13^CNMR (101 MHz, DMSO-*d*_6_) δ 23.4, 25.1, 36.1, 47.1, 93.8, 116.0, 119.2, 128.8, 129.2, 130.0, 131.0, 134.3, 137.0, 143.2, 161.4, 166.3, 166.4, 166.5. Calculated MS: 543.0802; experimental MS (ESI) *m*/*z* (%): 544.0680 [M + H].

##### 2-((4-(4-Chlorophenyl)-5-Cyano-6-Oxo-1,6-Dihydropyrimidin-2-yl)thio)-N-(4-((4-Methylpiperazin-1-yl)Sulfonyl)Phenyl)Acetamide (**M13**)


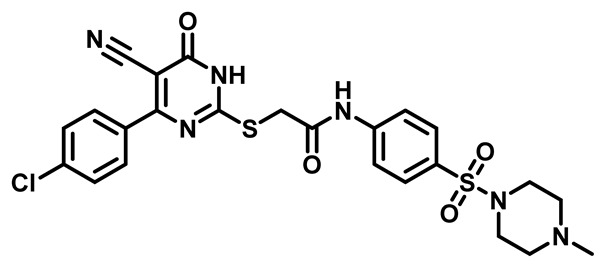


White powder, mp 225–227 °C, 657 mg, 93%, ^1^H NMR (400 MHz, DMSO-*d*_6_) δ 2.55 (s, 3H), 2.99 (s, 8H), 3.91 (s, 2H), 7.39 (d, J = 8.6 Hz, 2H), 7.65 (d, J = 8.7 Hz, 2H), 7.74 (d, J = 8.6 Hz, 2H), 7.78 (d, J = 8.7 Hz, 2H), 11.15 (s, 1H). ^13^CNMR (101 MHz, DMSO-*d*_6_) δ 35.9, 43.4, 44.3, 52.7, 91.0, 118.8, 119.3, 128.3, 128.8, 129.5, 129.8, 130.6, 135.7, 135.9, 144.1, 166.4, 168.3. Calculated MS: 558.0911; experimental MS (ESI) *m*/*z* (%): 559.0910 [M + H].

##### 2-((4-(4-Chlorophenyl)-5-Cyano-6-Oxo-1,6-Dihydropyrimidin-2-yl)thio)-N-(4-(Morpholinosulfonyl)Phenyl)Acetamide (**M14**)


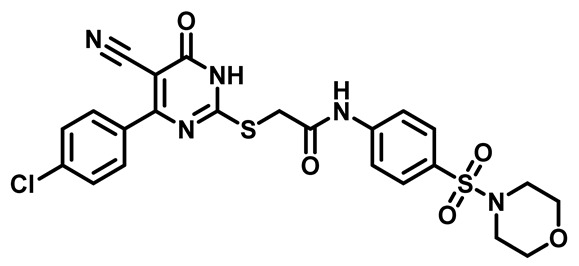


White powder, mp 198–200 °C, 646 mg, 91%, ^1^H NMR (400 MHz, DMSO-*d*_6_) δ 2.86 (t, J = 3.8 Hz, 4H), 3.64 (t, J = 4.7 Hz, 4H), 4.21 (s, 2H), 7.36 (d, J = 8.6 Hz, 2H), 7.70 (d, J = 8.8 Hz, 2H), 7.83 (d, J = 8.8 Hz, 2H), 7.85 (d, J = 8.7 Hz, 2H), 10.80 (s, 1H). ^13^CNMR (101 MHz, DMSO-*d*_6_) δ 36.2, 46.4, 65.8, 93.7, 116.2, 119.3, 128.8, 128.9, 129.5, 131.0, 134.4, 136.9, 143.6, 166.3, 166.6, 166.7. Calculated MS: 545.0594; experimental MS (ESI) *m*/*z* (%): 546.0620 [M + H].

##### 2-((4-(4-Chlorophenyl)-5-Cyano-6-Oxo-1,6-Dihydropyrimidin-2-yl)Thio)-N-(4-(Thiomorpholinosulfonyl)Phenyl)Acetamide (**M15**)


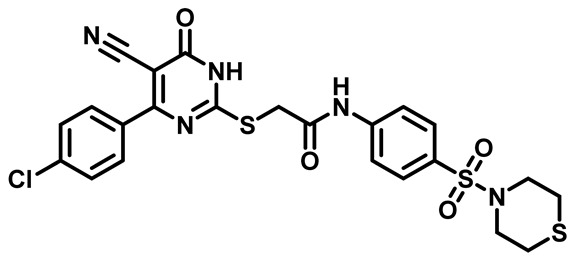


White powder, mp 210–214 °C, 686 mg, 94%, ^1^H NMR (400 MHz, DMSO-*d*_6_) δ 2.66 (t, J = 4.7, 4.3 Hz, 4H), 3.19 (t, J = 4.5 Hz, 4H), 4.21 (s, 2H), 7.34 (d, J = 8.6 Hz, 2H), 7.70 (d, J = 8.8 Hz, 2H), 7.81 (d, J = 8.7 Hz, 2H), 7.85 (d, J = 8.6 Hz, 2H), 10.76 (s, 1H). ^13^CNMR (101 MHz, DMSO-*d*_6_) δ 26.9, 36.2, 48.3, 93.8, 116.0, 119.3, 128.9, 129.0, 130.5, 131.0, 134.3, 137.0, 143.5, 161.4, 166.3, 166.6. Calculated MS: 561.0366; experimental MS (ESI) *m*/*z* (%): 562.0420 [M + H].

##### 2-((4-(4-Fluorophenyl)-5-Cyano-6-Oxo-1,6-Dihydropyrimidin-2-yl)Thio)-N-(4-(N,N-Diethylsulfamoyl)Phenyl)Acetamide (**M16**)


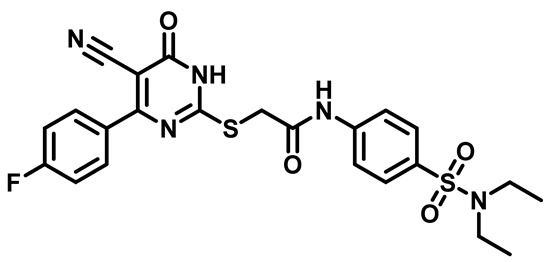


White powder, mp 248–251 °C, 630 mg, 94%, ^1^H NMR (400 MHz, DMSO-*d*_6_) δ 1.03 (t, J = 7.1 Hz, 6H), 3.15 (q, J = 7.1 Hz, 4H), 4.21 (s, 2H), 7.13 (t, J = 8.8 Hz, 2H), 7.72–7.79 (m, 4H), 7.93 (dd, J = 8.8, 5.5 Hz, 2H), 10.72 (s, 1H), 13.96 (bs, 1H). ^19^FNMR (376 MHz, DMSO) δ −107.90. ^13^CNMR (101 MHz, DMSO-*d*_6_) δ 14.4, 36.1, 42.1, 93.5, 115.7, 115.9, 116.2, 119.3, 128.5, 131.9, 131.9, 134.3, 142.9, 163.1, 165.6, 166.3, 166.5. Calculated MS: 515.1097; experimental MS (ESI) *m*/*z* (%): 538.0989 [M + Na].

##### 2-((4-(4-Fluorophenyl)-5-Cyano-6-Oxo-1,6-Dihydropyrimidin-2-yl)Thio)-N-(4-(Piperidin-1-Ylsulfonyl)Phenyl)Acetamide (**M17**)


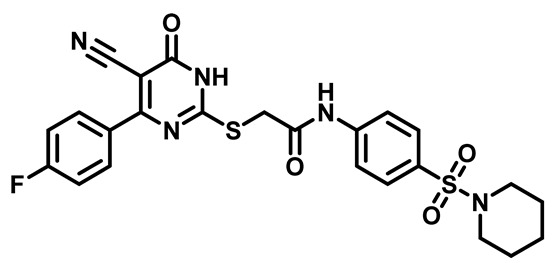


White powder, mp 177–180 °C, 630 mg, 92%, ^1^H NMR (400 MHz, DMSO-*d*_6_) δ 1.36 (p, 2H), 1.54 (t, J = 5.7 Hz, 4H), 2.86 (t, J = 5.4 Hz, 4H), 4.22 (s, 2H), 7.11 (t, J = 8.8 Hz, 2H), 7.69 (d, J = 8.7 Hz, 2H), 7.80 (d, J = 8.7 Hz, 2H), 7.93 (dd, J = 8.7, 5.6 Hz, 2H), 10.75 (s, 1H), 13.95 (bs, 1H). ^19^FNMR (376 MHz, DMSO) δ −107.98. ^13^CNMR (101 MHz, DMSO-*d*_6_) δ 23.4, 25.1, 36.2, 47.1, 93.5, 115.7, 115.9, 116.2, 119.2, 129.2, 130.0, 131.9, 132.0, 143.3, 163.1, 165.6, 166.2, 166.3, 166.5. Calculated MS: 527.1097; experimental MS (ESI) *m*/*z* (%): 528.1159 [M + H].

##### 2-((4-(4-Fluorophenyl)-5-Cyano-6-Oxo-1,6-Dihydropyrimidin-2-yl)Thio)-N-(4-((4-Methylpiperazin-1-yl)Sulfonyl)Phenyl)Acetamide (**M18**)


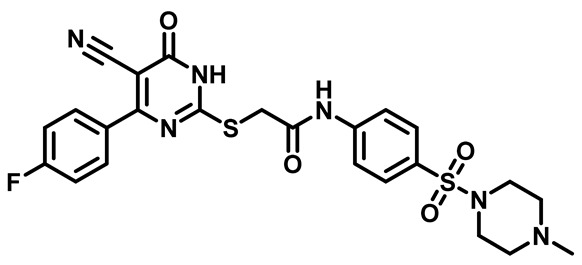


White powder, mp 213–215 °C, 641 mg, 91%, ^1^H NMR (400 MHz, DMSO-*d*_6_) δ 2.60 (s, 3H), 3.04 (s, 8H), 3.98 (s, 2H), 7.23 (t, *J* = 8.9 Hz, 2H), 7.73 (d, *J* = 8.8 Hz, 2H), 7.83–7.90 (m, 4H), 11.24 (s, 1H). ^19^FNMR (376 MHz, DMSO) δ −110.06. ^13^C NMR (101 MHz, DMSO-*d*_6_) δ 35.9, 43.5, 44.4, 52.8, 90.9, 115.5, 115.8, 118.9, 119.4, 128.3, 129.5, 131.2, 131.3, 133.5, 133.6, 144.0, 162.5, 165.0, 166.5, 167.8, 168.3, 170.2. Calculated MS: 542.1206; experimental MS (ESI) *m*/*z* (%): 543.1252 [M + H].

##### 2-((4-(4-Fluorophenyl)-5-Cyano-6-Oxo-1,6-Dihydropyrimidin-2-yl)Thio)-N-(4-(Morpholinosulfonyl)Phenyl)Acetamide (**M19**)


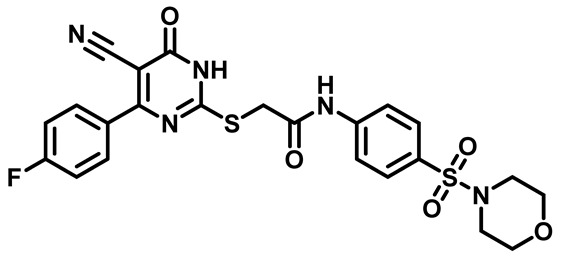


White powder, mp 270–274 °C, 640 mg, 93%, ^1^H NMR (400 MHz, DMSO-*d*_6_) δ 2.85 (t, *J* = 4.2 Hz, 4H), 3.63 (t, *J* = 4.7 Hz, 4H), 4.24 (s, 2H), 7.14 (t, *J* = 8.8 Hz, 2H), 7.70 (d, *J* = 8.9 Hz, 2H), 7.82 (d, *J* = 8.9 Hz, 2H), 7.94 (dd, *J* = 8.7, 5.6 Hz, 2H), 10.77 (s, 1H), 13.89 (bs, 1H). ^19^FNMR (376 MHz, DMSO) δ −107.78. ^13^CNMR (101 MHz, DMSO-*d*_6_) δ 36.2, 46.4, 65.8, 93.6, 115.8, 116.0, 116.1, 119.3, 128.9, 129.5, 131.9, 132.0, 143.6, 161.4, 163.1, 165.6, 166.1, 166.4, 166.6. Calculated MS: 529.0890; experimental MS (ESI) *m*/*z* (%): 530.7637 [M + H].

##### 2-((4-(4-Fluorophenyl)-5-Cyano-6-Oxo-1,6-Dihydropyrimidin-2-yl)Thio)-N-(4-(Thiomorpholinosulfonyl)Phenyl)Acetamide (**M20**)


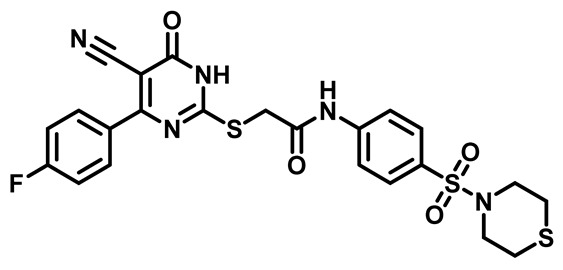


White powder, mp 195–198 °C, 652 mg, 92%, ^1^H NMR (400 MHz, DMSO-*d*_6_) δ 2.65 (t, *J* = 5.2 Hz, 4H), 3.18 (t, *J* = 5.0 Hz, 4H), 4.21 (s, 2H), 7.12 (t, *J* = 8.7 Hz, 2H), 7.70 (d, *J* = 8.7 Hz, 2H), 7.79 (d, *J* = 8.6 Hz, 2H), 7.92 (dd, *J* = 8.6, 5.4 Hz, 2H), 10.78 (s, 1H). ^19^FNMR (376 MHz, DMSO-*d*_6_) δ −107.72. ^13^CNMR (101 MHz, DMSO-*d*_6_) δ 26.9, 36.1, 48.2, 93.5, 115.8, 116.0, 116.2, 119.4, 129.0, 130.6, 131.8, 131.9, 143.4, 161.5, 163.1, 166.2, 166.4, 166.6. Calculated MS: 545.0661; experimental MS (ESI) *m*/*z* (%): 546.6345 [M + H].

##### 2-((4-(3-Chlorophenyl)-5-Cyano-6-Oxo-1,6-Dihydropyrimidin-2-yl)thio)-N-(4-(N,N-Diethylsulfamoyl)Phenyl)Acetamide (**M21**)


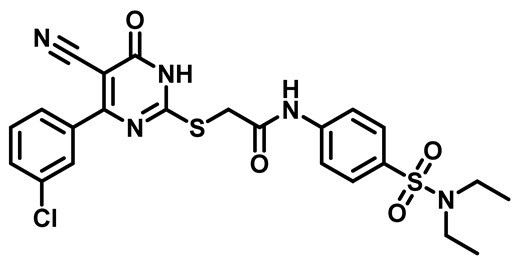


White powder, mp 255–258 °C, 608 mg, 88%, ^1^H NMR (400 MHz, DMSO-*d*_6_) δ 1.02 (t, *J* = 7.1 Hz, 6H), 3.13 (q, *J* = 7.1 Hz, 4H), 4.20 (s, 2H), 7.36 (t, *J* = 7.9 Hz, 1H), 7.56 (d, *J* = 7.4 Hz, 1H), 7.68–7.73 (m, 4H), 7.74–7.81 (m, 2H), 10.71 (s, 1H). ^13^C NMR (101 MHz, DMSO-*d*_6_) δ 14.4, 36.1, 42.2, 94.3, 115.9, 119.5, 127.8, 128.4, 128.6, 130.7, 131.8, 133.8, 134.4, 137.5, 142.8, 161.4, 166.2, 166.3, 166.6. Calculated MS: 531.0802; experimental MS (ESI) *m*/*z* (%): 530.0735 [M + H].

##### 2-((4-(3-Chlorophenyl)-5-Cyano-6-Oxo-1,6-Dihydropyrimidin-2-yl)thio)-N-(4-(Piperidin-1-ylsulfonyl)Phenyl)Acetamide (**M22**)


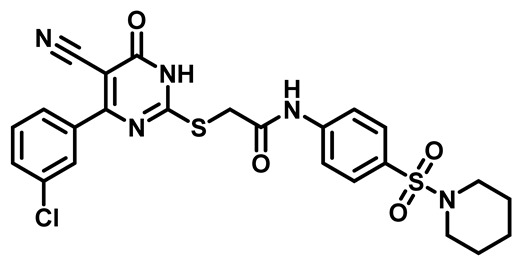


White powder, mp 262–265 °C, 629 mg, 89%, ^1^H NMR (400 MHz, DMSO-*d*_6_) δ 1.35 (p, *J* = 5.5 Hz, 2H), 1.54 (p, 4H), 2.84 (t, *J* = 5.0 Hz, 4H), 4.21 (s, 2H), 7.38 (t, *J* = 8.0 Hz, 1H), 7.55 (d, *J* = 7.9 Hz, 1H), 7.65 (d, *J* = 8.6 Hz, 2H), 7.75–7.82 (m, 4H), 10.75 (s, 1H). ^13^CNMR (101 MHz, DMSO-*d*_6_) δ 23.4, 25.1, 36.1, 47.1, 94.3, 116.0, 119.3, 127.8, 128.6, 129.1, 130.0, 130.7, 131.8, 133.7, 137.6, 143.3, 166.2, 166.4, 166.7. Calculated MS: 543.0802; experimental MS (ESI) *m*/*z* (%): 542.0736 [M + H].

##### 2-((4-(3-Chlorophenyl)-5-Cyano-6-Oxo-1,6-Dihydropyrimidin-2-yl)thio)-N-(4-((4-Methylpiperazin-1-yl)Sulfonyl)Phenyl)Acetamide (**M23**)


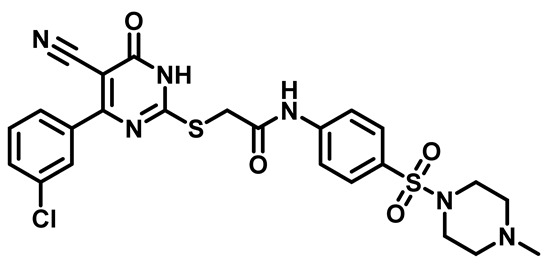


White powder, mp 242–245 °C, 617 mg, 85%, ^1^H NMR (400 MHz, DMSO-*d*_6_) δ 2.67 (s, 3H), 3.11 (s, 8H), 4.01 (s, 2H), 7.44 (t, J = 7.9 Hz, 1H), 7.56 (d, J = 7.6 Hz, 1H), 7.75 (dd, 4H), 7.87 (d, J = 8.8 Hz, 2H), 11.19 (s, 1H).^13^C NMR (101 MHz, DMSO-*d*_6_) δ 35.9, 43.1, 44.1, 52.5, 91.5, 118.5, 119.4, 127.5, 128.3, 128.4, 129.5, 130.6, 130.7, 133.5, 139.1, 144.1, 166.1, 168.1, 170.3. Calculated MS: 558.0911; experimental MS (ESI) *m*/*z* (%): 557.0842 [M + H].

##### 2-((4-(3-Chlorophenyl)-5-Cyano-6-Oxo-1,6-Dihydropyrimidin-2-yl)thio)-N-(4-(Morpholinosulfonyl)Phenyl)Acetamide (**M24**)


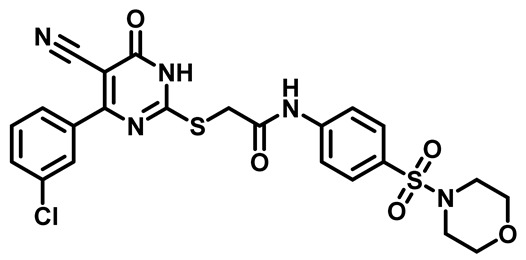


White powder, mp 261–264 °C, 582 mg, 82%, ^1^H NMR (400 MHz, DMSO-*d*_6_) δ 2.76 (t, J = 4.4 Hz, 4H), 3.57 (t, J = 3.5 Hz, 4H), 4.16 (s, 2H), 7.29 (t, J = 7.9 Hz, 1H), 7.46–7.51 (m, 1H), 7.60 (d, J = 8.8 Hz, 2H), 7.66–7.80 (m, 4H), 10.70 (s, 1H), 13.96 (bs, 1H).^13^CNMR (101 MHz, DMSO-*d*_6_) δ 36.2, 46.4, 65.8, 94.4, 115.9, 119.4, 127.8, 128.6, 128.8, 129.4, 130.7, 131.8, 133.8, 137.5, 143.6, 161.3, 166.2, 166.4, 166.6. Calculated MS: 545.0594; experimental MS (ESI) *m*/*z* (%): 544.0529 [M + H].

##### 2-((4-(3-Chlorophenyl)-5-Cyano-6-Oxo-1,6-Dihydropyrimidin-2-yl)thio)-N-(4-(Thiomorpholinosulfonyl)Phenyl)Acetamide (**M25**)


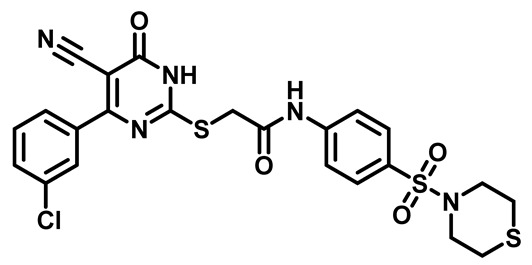


White powder, mp 245–247 °C, 613 mg, 84%, ^1^H NMR (400 MHz, DMSO-*d*_6_) δ 2.67 (t, J = 6.2 Hz, 4H), 3.18 (t, J = 4.1 Hz, 4H), 4.20 (s, 2H), 7.36 (t, J = 7.9 Hz, 1H), 7.54–7.60 (m, 1H), 7.68 (d, J = 8.8 Hz, 2H), 7.76–7.82 (m, 4H), 10.83 (s, 1H). ^13^CNMR (101 MHz, DMSO-*d*_6_) δ 26.9, 48.3, 116.3, 119.4, 127.8, 128.6, 129.0, 130.4, 130.6, 131.7, 133.7, 137.8, 143.5, 166.2, 166.6. Calculated MS: 561.0366; experimental MS (ESI) *m*/*z* (%): 562.0429 [M + H].

### 3.2. Biological Evaluation

#### 3.2.1. Materials

This study utilized 25 chemical compounds labeled **M1** to **M25**. All compounds were tested at a concentration of 3000 µg/mL.

#### 3.2.2. Bacterial Strains

The antibacterial activity of the compounds was assessed against a panel of bacterial strains. More details are presented in the file in Supplementary Materials.

#### 3.2.3. Agar Diffusion-Based Screening of Antimicrobial Activity

The antibacterial screening was performed using the agar diffusion method [51]. More details are presented in the file in Supplementary Materials.

#### 3.2.4. Measurement of Inhibition Zones

The antibacterial activity was evaluated by measuring the diameter of the inhibition zones in millimeters (mm) around each well. More details are presented in the file in Supplementary Materials.

#### 3.2.5. Determination of Minimum Inhibitory Concentration (MIC)

The minimum inhibitory concentration (MIC) was determined by macro-dilution method [41,42]. More details are presented in the file in Supplementary Materials.

#### 3.2.6. Determination of Minimum Bactericidal Concentration (MBC)

The minimum bactericidal concentration (MBC) was determined as described [42], following the MIC assay, by subculturing 100 µL of the compounds from wells showing no visible growth onto fresh agar plates. The plates were incubated at 37 °C for 24 h. The MBC was defined as the lowest concentration of the compound that resulted in a 99.9% reduction in the initial bacterial inoculum. More details are presented in the file in Supplementary Materials.

#### 3.2.7. Antibiofilm Assay of the Selected Compounds by Tissue Culture Plate Method (TCP)

Inhibition of the initial adherence of *Klebsiella pneumonia* and *Pseudomonas aeruginosa* by the selected compounds was assessed according to the reported references [43,44]. More details are presented in the file in Supplementary Materials.

### 3.3. Calculation Physicochemical Properties and ADMET (Absorption, Distribution, Metabolism, Excretion, and Toxicity

The compounds were prepared in sdf format, and Data Warrior software (v06.02.03.) was used to calculate their physicochemical properties. Meanwhile, the pkCSM platform was used to calculate their ADMET properties using smiles of the compounds as input [47,48,49,50].

## 4. Conclusions

In this study, we have synthesized 25 novel analogues bearing a 5-cyano-6-oxo-4-substituted phenyl-1,6-dihydropyrimidine scaffold hybridized with various substituted benzenesulfonamide derivatives through a thioacetamide linker (**M1–25**). These compounds exhibited considerable promise as antimicrobial agents, demonstrating broad-spectrum efficacy against Gram-positive, Gram-negative, and fungal pathogens, with notable effectiveness against the resistant strains *K. pneumoniae* and *P. aeruginosa* with zones of inhibition values ranging from 15 to 30 mm. The minimum inhibitory concentration (MIC) values of the most promising derivatives, **M6**, **M19**, **M20**, and **M25**, were 375 µg/mL against both of these bacterial strains. On the other hand, the minimum bactericidal concentration (MBC) values showed variability among the compounds and bacterial strains. For *K. pneumoniae*, the MBC values for compounds **M6** and **M19** were 1500 µg/mL, while for compounds **M20** and **M25**, the MBC value was 7500 µg/mL. For *P. aeruginosa*, the MBC for all four compounds was consistent at 1500 µg/mL. The obtained results confirmed the bactericidal activity of compounds **M6** and **M19** against both *K. pneumonia* and *P. aeruginosa*, with a ratio of MBC/MIC equal to four, while the compounds **M20** and **M25** had MBC/MIC ratios that revealed their bactericidal activity against the *P. aeruginosa* strain and their bacteriostatic impact on *K. pneumoniae.* These data implied the newly synthesized sulfonamide derivatives have potential to be developed and optimized as bactericidal agents against some resistant strains.

The positive outcome of the minimum inhibitory concentration (MIC), minimum bactericidal concentration (MBC), and biofilm suppression assays underscores their capability to stop microbial growth and virulence. Preliminary ADMET (absorption, distribution, metabolism, excretion, and toxicity) profiling suggests that these derivatives possess favorable drug-like properties and manageable toxicity levels. Collectively, these findings underscore the potential of these pyrimidine–benzenesulfonamide derivatives as promising candidates for further development and optimization in the treatment of microbial infections.

## Figures and Tables

**Table 1:** Molecular structures of the target compounds **M1–25.**

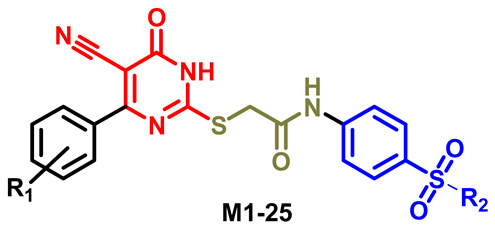					
Compounds	R_1_	R_2_	Compounds	R_1_	R_2_
**M1**	H	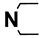	**M14**	4-Cl	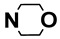
**M2**	H	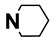	**M15**	4-Cl	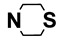
**M3**	H	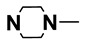	**M16**	4-F	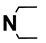
**M4**	H	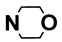	**M17**	4-F	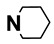
**M5**	H	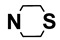	**M18**	4-F	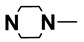
**M6**	4-Br	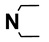	**M19**	4-F	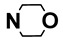
**M7**	4-Br	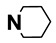	**M20**	4-F	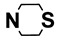
**M8**	4-Br	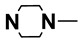	**M21**	3-Cl	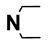
**M9**	4-Br	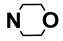	**M22**	3-Cl	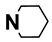
**M10**	4-Br	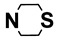	**M23**	3-Cl	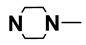
**M11**	4-Cl	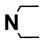	**M24**	3-Cl	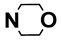
**M12**	4-Cl	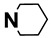	**M25**	3-Cl	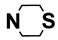
**M13**	4-Cl	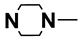			

**Table 2:** The antimicrobial potency of the new molecules **M1–25**, expressed as zone of inhibition (ZOI) in mm.

Microbial Organisms	M 1	M2	M3	M 4	M 5	M 6	M 7	M 8	M 9	M 10	M 11	M 12	M 13	M 14	M 15	M 16	M 17	M 18	M 19	M 20	M 21	M 22	M 23	M 24	M 25
***E. coli*** **ATCC-25922**	0	16	18	20	0	20	20	18	25	22	0	20	20	22	19	0	0	0	0	20	0	20	20	24	26
***K. pneumoniae***	20	22	24	22	22	26	22	22	24	24	20	17	15	22	22	24	24	22	28	26	20	20	19	26	26
***P. aeruginosa*** **ATCC 27853**	20	22	20	22	24	26	18	18	20	20	22	18	22	22	22	16	20	22	26	30	20	18	20	22	30
***S. aureus*** **ATCC 6538**	0	0	0	0	17	21	21	0	21	21	15	19	0	15	20	22	16	22	20	26	22	17	18	0	24
***S. epidermidis*** **ATCC 35984**	0	15	0	0	22	15	21	20	25	17	0	0	0	0	18	20	20	20	22	22	20	20	0	13	20
***B. subtilis*** **ATCC 6633**	18	22	0	0	22	20	22	17	26	15	18	22	0	13	18	23	25	0	24	15	15	20	0	13	28
***C. albicans*** **ATCC-10231**	0	0	0	0	17	21	21	0	21	21	15	19	0	15	18	23	16	23	20	26	22	17	18	0	24

**Table 3:** MIC and MBC results for the most potent compounds against the respiratory bacterial strains.

Chemical Compounds	Bacterial Strains	MIC μg/mL	MBC μg/mL
**M6**	*Klebsiella pneumoniae*	375 ± 0.00	1500 ± 0.45
	*Pseudomonas aeruginosa*	375 ± 0.00	1500 ± 0.00
**M19**	*Klebsiella pneumoniae*	375 ± 0.00	1500 ± 0.29
	*Pseudomonas aeruginosa*	375 ± 0.00	1500 ± 0.00
**M20**	*Klebsiella pneumoniae*	375 ± 0.00	7500 ± 0.00
	*Pseudomonas aeruginosa*	375 ± 0.00	1500 ± 0.00
**M25**	*Klebsiella pneumoniae*	375 ± 0.00	7500 ± 0.00
	*Pseudomonas aeruginosa*	375 ± 0.00	1500 ± 0.00

**Table 4:** Physicochemical properties of the most promising compounds and their adherence to Lipinski’s rule.

Compound No.	MW	HBA	HBD	logP (o/w)	TPSA Å^2^	Num. Rotatable Bonds	Lipinski
**M6**	576.49	6	2	3.04	169.7	8	Yes; 1 violation: MW > 500
**M19**	546.02	7	2	1.72	178.9	6	Yes; 1 violation: MW > 500
**M20**	562.09	6	2	2.35	195	6	Yes; 1 violation: MW > 500
**M25**	562.09	6	2	2.39	195	6	Yes; 1 violation: MW > 500

MW = molecular weight, HBA = hydrogen bond acceptor, HBD = hydrogen bond donor, log Po/w = octanol-water partition coefficient, TPSA = total polar surface area.

**Table 5:** In silico ADMET prediction of compounds **M6**, **M19**, **M20**, and **M25**.

	Compound M6	Compound M19	Compound M20	Compound M25
Absorption Water solubility (log mol/L) Intestinal absorption Skin permeability (log Kp)	−4.0 82.4 −2.7	−3.8 78.7 −2.7	−3.8 85.1 −2.7	−3.8 85.2 −2.7
Distribution Blood–brain permeability(log BB) CNS permeability (log PS)	−1.46 −2.78	−1.43 −3.45	−1.42 −2.73	−1.42 −2.73
Metabolism CYP2D6 substrate CYP3A4 substrate CYP1A2 inhibitor	No Yes No	No Yes No	No Yes No	No Yes No
Excretion Total clearance (log mL/min/Kg) Renal OCT2 substrate	0.1 No	0.2 No	0.1 No	0.1 No
Toxicity AMES toxicity hERG inhibitor Tumorigenic Irritant	No No No No	No No No No	No No No No	No No No No

## Data Availability

Data will be made available upon request. A.K., M.M.A., W.A.A., E.A.E.-G, S.A.E., S.H.A.

## Supplementary Materials

The following supporting information can be downloaded at: https://www.mdpi.com/article/10.3390/molecules29194778/s1, The NMR spectra (^1^H, ^13^C, ^19^F) for all synthesized compounds are presented in Supplementary Figures S1–S94, while the mass spectrometry data for the M1–25 compounds are included in Supplementary Figures S95–S114.
