# Supplementary material for: Design, Synthesis, and Antimicrobial Evaluation of New Thiopyrimidine–Benzenesulfonamide Compounds
Source: Molecules. 2024 Oct 9;29(19):4778. doi: 10.3390/molecules29194778 (PMC11477697; doi:10.3390/molecules29194778)
Supplement: Supplementary file 1 [file molecules-29-04778-s001.zip › molecules-3199340-supplementary.pdf]

Supporting Information

Design, synthesis, and antimicrobial evaluation of new thiopyrimidine-benzenesulfonamide compounds

Abdalrahman Khalifa [a,b], Manal M. Anwar [c] , Walaa A. Alshareef [d], Eman A. El-Gebaly [d], Samia A. Elseginy [e], Sameh H. Abdelwahed [a,*].

No.	Contents	Page
	4. Experimental	2
1	4.1 Chemistry	2
2	4.2 Biological Evaluation	2-3
3	^{1}H-NMR and ^{13}C-NMR [NMR Spectra of All Synthesized Compounds]	4-98
4-	Mass Spectrometry Analysis [Synthesized Compounds]	99-116
4	References	117

4. Experimental

4.1. Chemistry

All reagents and solvents were purchased from commercial suppliers and used without purification unless stated otherwise. NMR spectra were recorded using Brucker 400 MHz instrument available at Prairie View A&M University and Texas A&M University. All the samples were run in DMSO-d_6 except sample 4a and 4d were run in $CDCl_3$. Chemical shifts (δ) for ^{1}H NMR spectra run in DMSO-*d6* are reported in ppm relative to DMSO residual solvent protons (δ 2.5). Chemical shifts for ^{13}C NMR spectra run in DMSO-d_6 are reported in ppm relative to the solvent residual carbon (δ 39.5). Chemical shifts (δ) for ^{1}H NMR spectra run in DMSO-d_6 are reported in ppm relative to DMSO residual solvent protons (δ 2.05). Chemical shifts for ^{13}C NMR spectra run in DMSO-d_6 are reported in ppm relative to the solvent residual carbons (δ 30.8). Peaks in NMR spectra are described as follows: singlet (s), broad singlet (bs), doublet (d) triplet (t), doublet of doublets (dd). Melting points are measured using Stuart SMP 30 and are uncorrected. Follow up of the reactions and checking the purity of the compounds were made by TLC on silica gel-precoated aluminium sheets (Type 60, F 254, Merck, Darmstadt, Germany) using chloroform/methanol (20:1, v/v) and the spots were detected by exposure to UV lamp at λ254 nanometer for few seconds and by iodine vapor. The chemical names given for the prepared compounds are according to the IUPAC system.

4.2. Biological evaluation

4.2.1. Materials

This study utilized 25 chemical compounds labeled 1 to 25. All compounds were tested at a concentration of 3000 μg/mL.

4.2.2. Bacterial Strains

The antibacterial activity of the compounds was assessed against a panel of bacterial strains, including *Escherichia coli*, *Klebsiella pneumoniae*, *Pseudomonas aeruginosa*, *Staphylococcus aureus*, *Staphylococcus epidermidis*, *Bacillus subtilis*, and the yeast strain *Candida albicans*. More details are presented in the file of Supplementary information.

4.2.3. Agar diffusion-based screening of antimicrobial activity

The antibacterial screening was performed using the agar diffusion method [1, 2]. Each bacterial strain was cultured in nutrient broth until reaching a turbidity equivalent to 0.05 McFarland standard, ensuring uniform bacterial inoculum. Sterile agar plates were prepared and allowed to solidify. Each plate was inoculated with bacterial suspension using a sterile swab to ensure even distribution. Wells were then punched into the agar, and 100 μL of each chemical compound (3000 μg/ml) was added to the respective wells. The plates were incubated at 37°C for 24 hours. More details are presented in the file of Supplementary information.

4.2.4. Measurement of Inhibition Zones

The antibacterial activity was evaluated by measuring the diameter of the inhibition zones in millimeters (mm) around each well. The zone of inhibition was recorded for each compound against the different bacterial strains, indicating the potency of the compounds. More details are presented in the file of Supplementary information.

4.2.5. Determination of Minimum Inhibitory Concentration (MIC)

The Minimum Inhibitory Concentration (MIC) was determined by macro dilution method as [3, 4] for the most potent compounds against Gram-negative bacteria, specifically *Pseudomonas*

aeruginosa and *Klebsiella pneumoniae*, as these organisms are significant pathogens responsible for pneumonia and respiratory diseases and are among the most resistant strains.

The MIC was assessed for Compounds M6, M19, M20, and M25. Serial dilutions of these compounds were prepared in nutrient broth (2-fold dilution), and the MIC was defined as the lowest concentration at which visible growth was inhibited after 24 hours of incubation at 37°C. More details are presented in the file of Supplementary information.

4.2.6. Determination of Minimum Bactericidal Concentration (MBC)

The Minimum Bactericidal Concentration (MBC) was determined as [5] following the MIC assay by subculturing 100 µL from each well that showed no visible growth onto fresh agar plates. The plates were incubated at 37°C for 24 hours. The MBC was defined as the lowest concentration of the compound that resulted in a 99.9% reduction in the initial bacterial inoculum. More details are presented in the file of Supplementary information.

4.2.7. Antibiofilm assay of the selected compounds by tissue culture plate method (TCP):

Inhibition of the initial adherence by *Klebsiella pneumonia* and *Pseudomonas aeruginosa* was by the selected compounds was assessed according to reported references [1, 6-8], where three appropriate concentrations (MIC, 2 MIC, and 4 MIC mg/mL) of the compounds were prepared in Tryptic Soy Broth (TSB) medium. Of each concentration, 100 L was transferred into wells, followed by the addition of TSB cultures (100 L). After incubation, the plates were dried, and the biofilms were stained with crystal violet (0.1%, 15 min.). After washing with phosphate-buffered saline (PBS), the wells were filled with ethanol (96%) and incubated for 20 min, and the absorbance was measured at 595 nm using a microplate reader. The inhibition (%) of biofilm formation = [OD negative control OD sample)/OD negative control] 100 [5-7]. Three control wells—wells containing tested bacteria + TSB (negative control), wells containing TSB + compound concentration (compound control), and wells containing TSB (media control)—were maintained for each test batch. More details are presented in the file of Supplementary information.

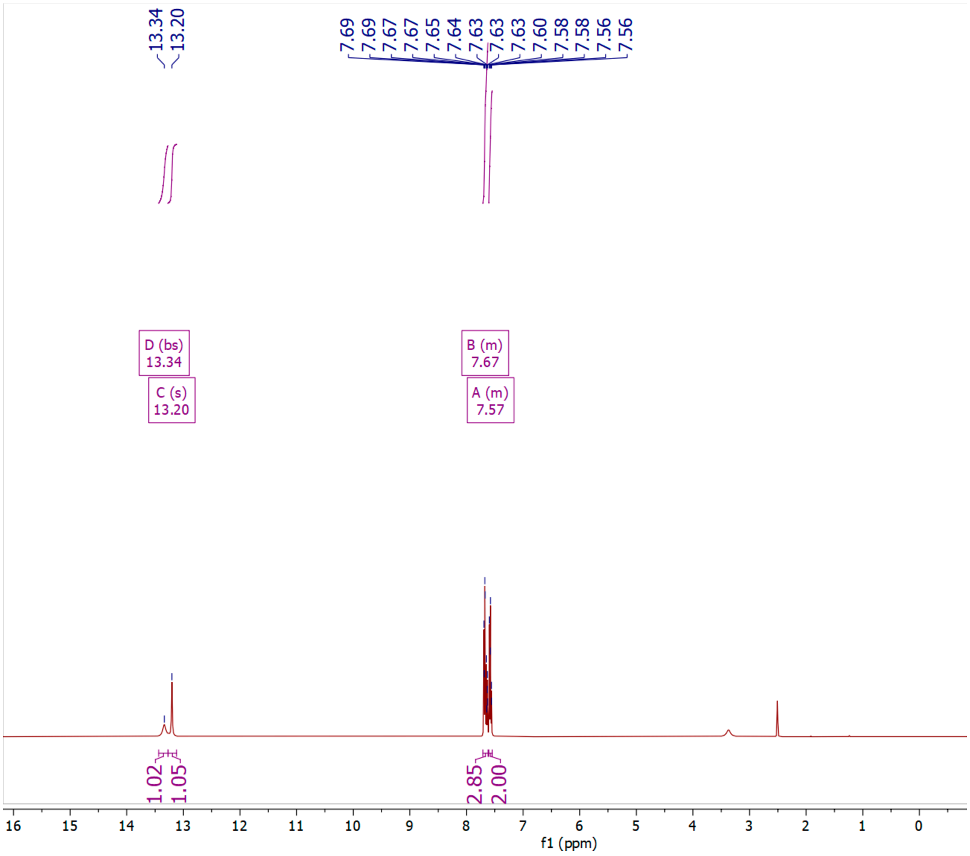

Figure S1. ¹H NMR spectrum of compound 1a

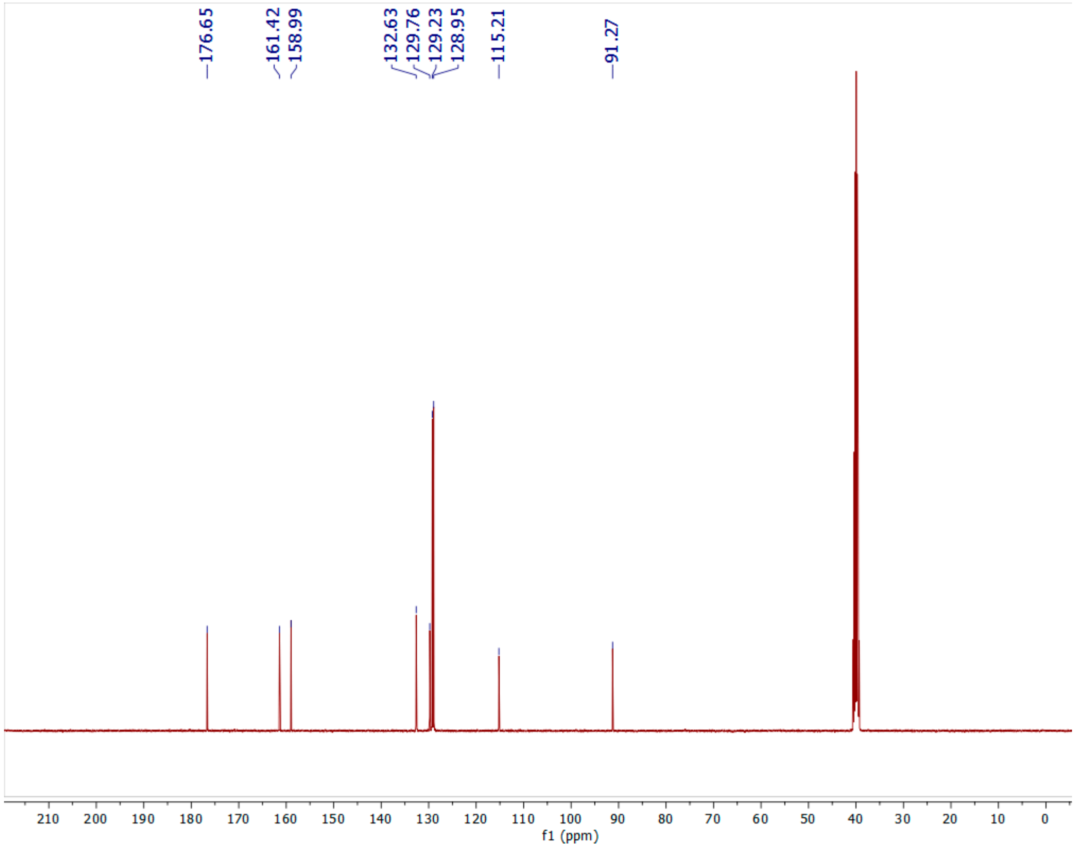

Figure S2. ^{13}CNMR spectrum of compound 1a

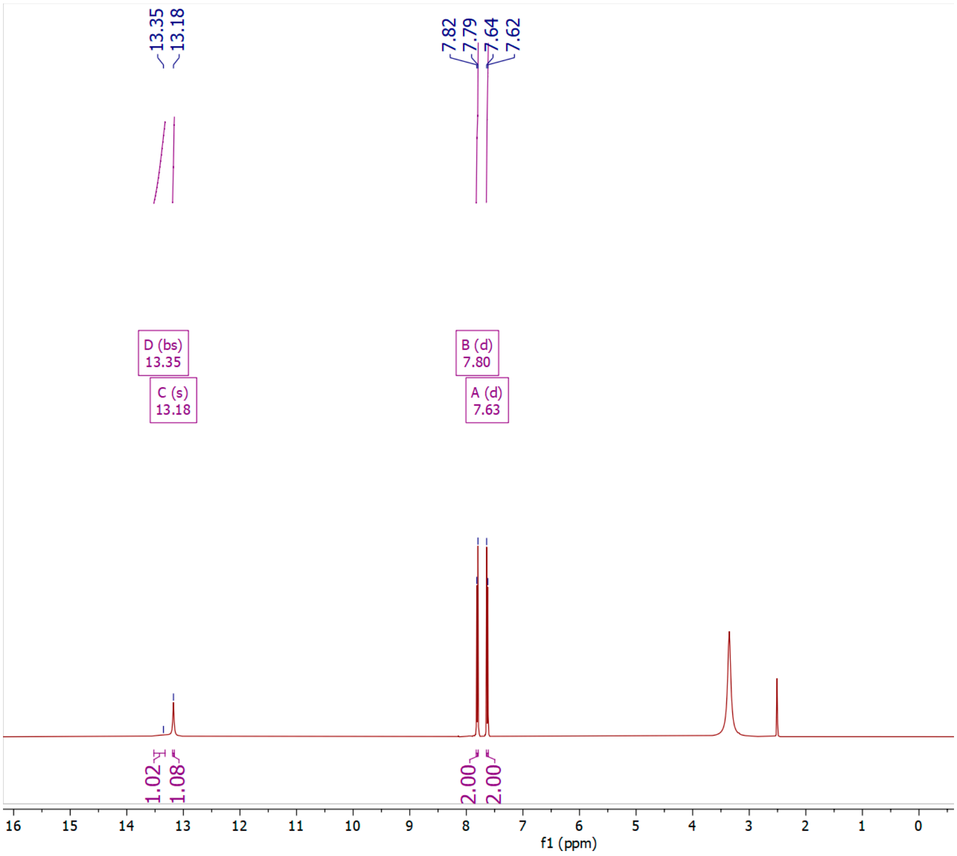

Figure S3. ^{1}H NMR spectrum of compound 1b

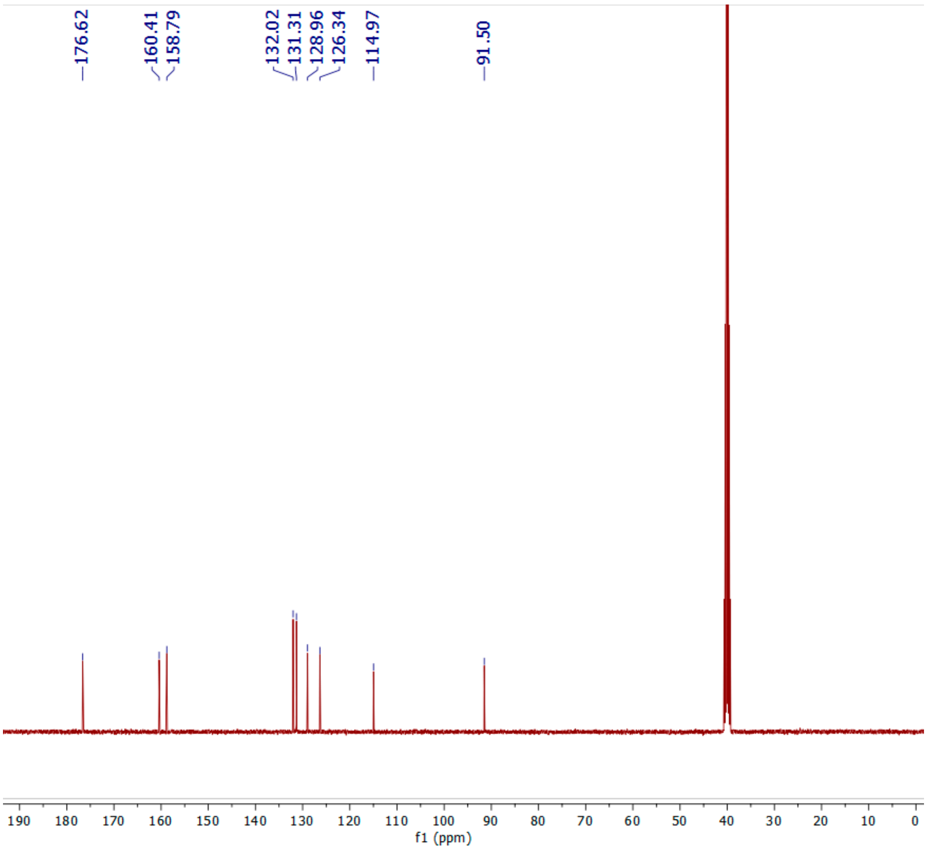

Figure S4. ^{13}CNMR spectrum of compound 1b

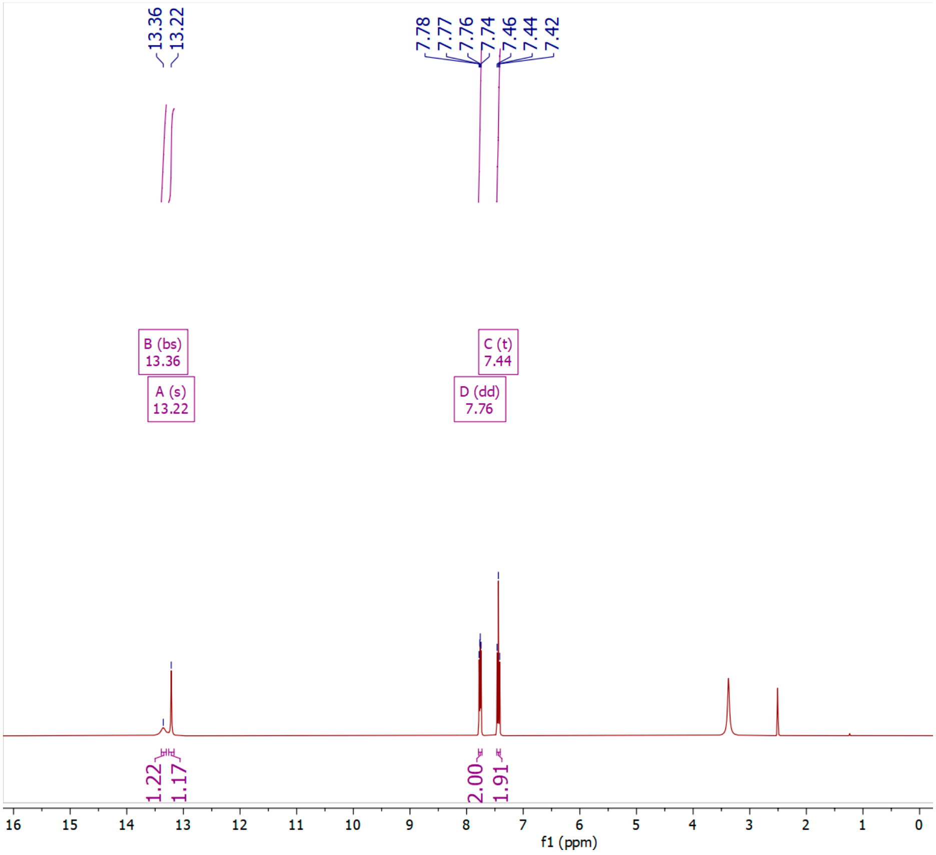

Figure S5. ^{1}HNMR spectrum of compound 1c

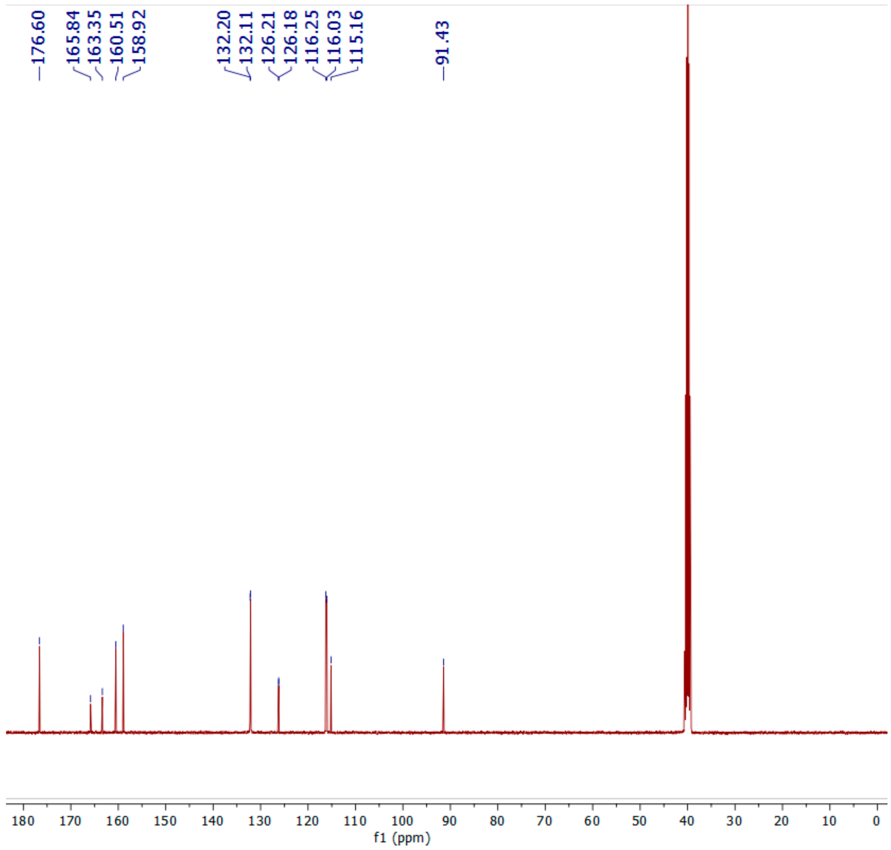

Figure S6. ^{13}CNMR spectrum of compound 1c

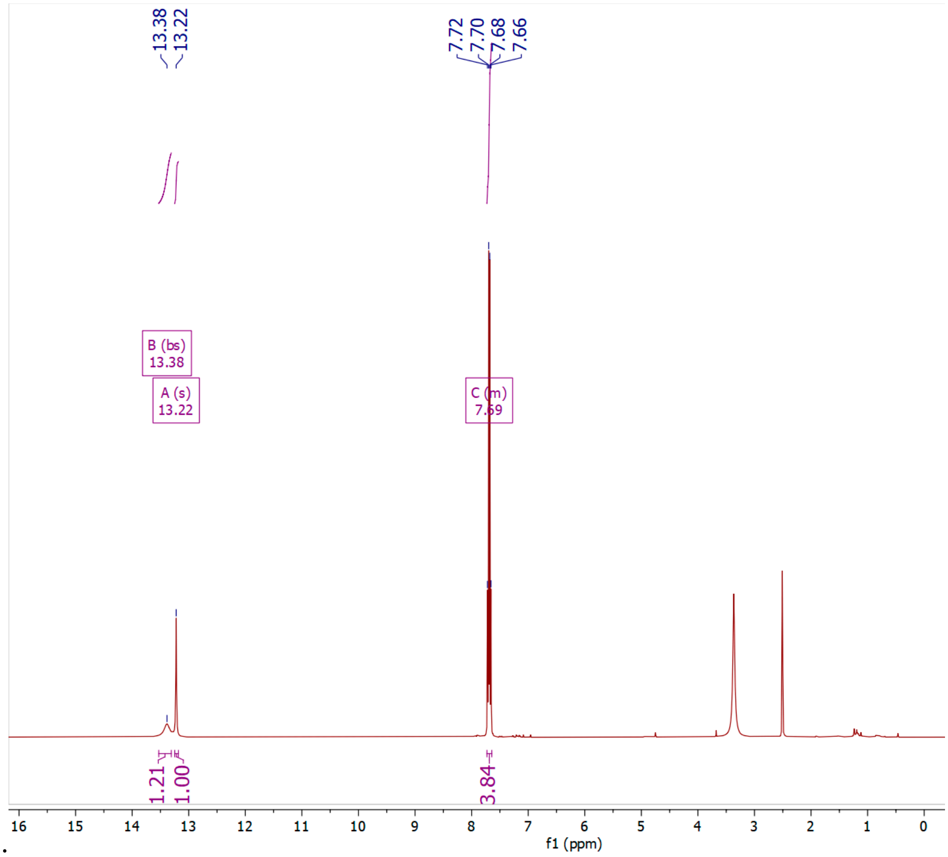

Figure S7. ^{1}HNMR spectrum of compound 1d

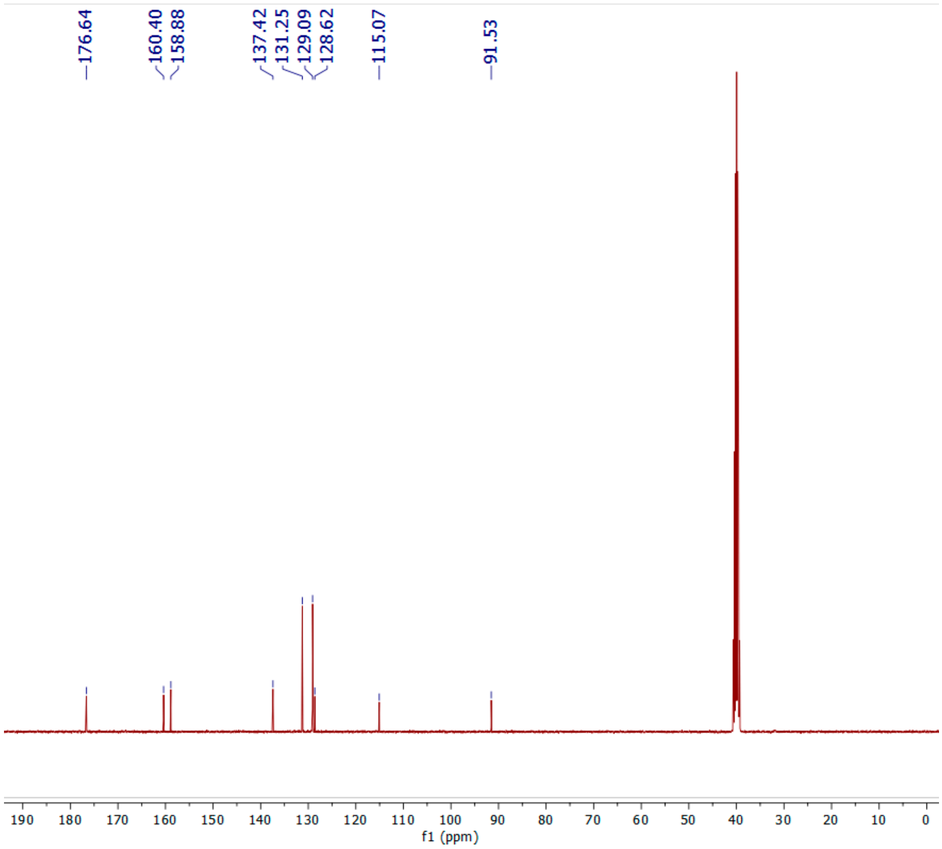

Figure S8. ^{13}CNMR spectrum of compound 1d

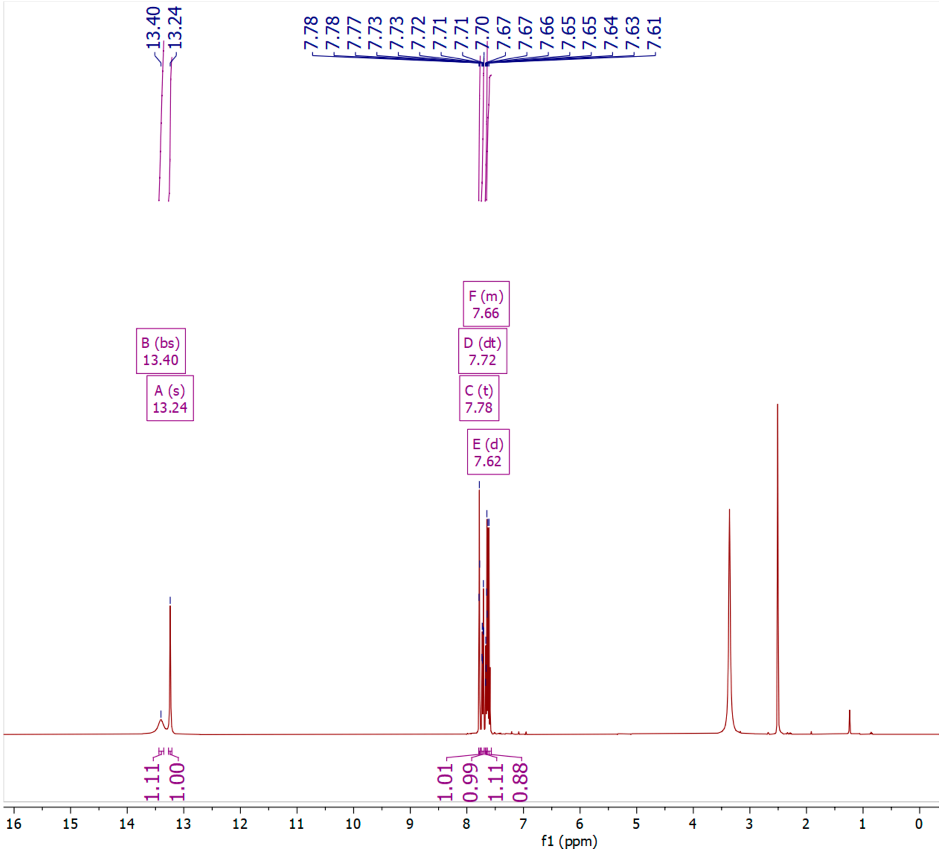

Figure S9. ^{1}HNMR spectrum of compound 1e

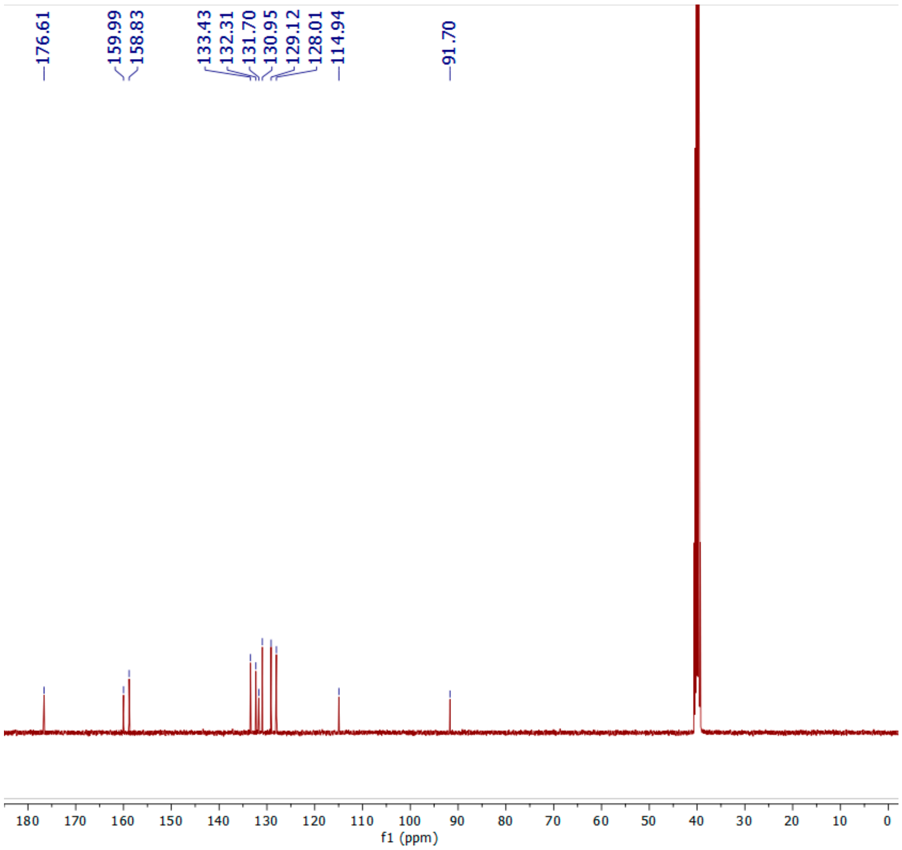

Figure S10. ^{13}CNMR spectrum of compound 1e

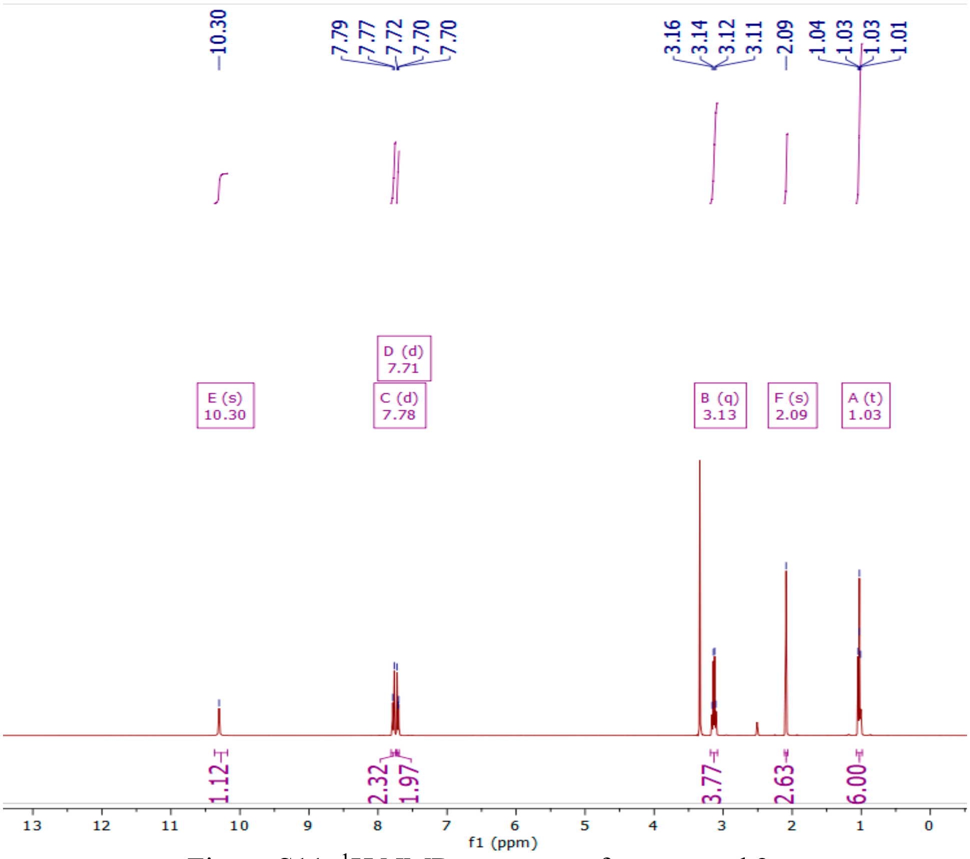

Figure S11. ^{1}H NMR spectrum of compound 2a

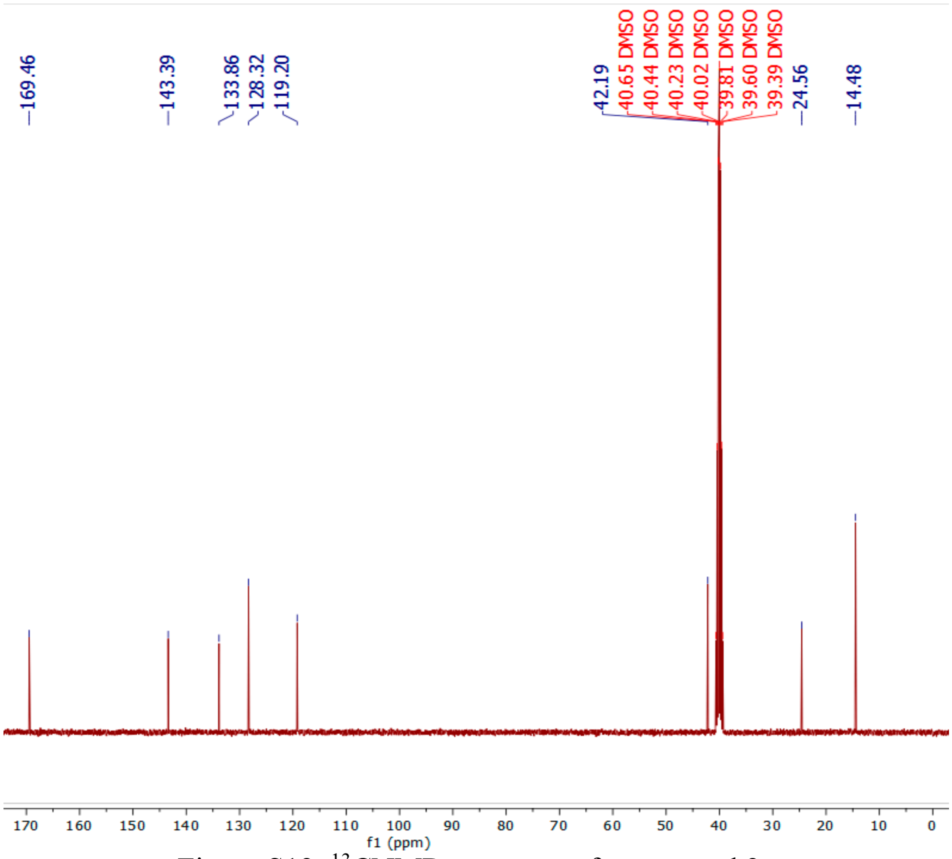

Figure S12. ^{13}CNMR spectrum of compound 2a

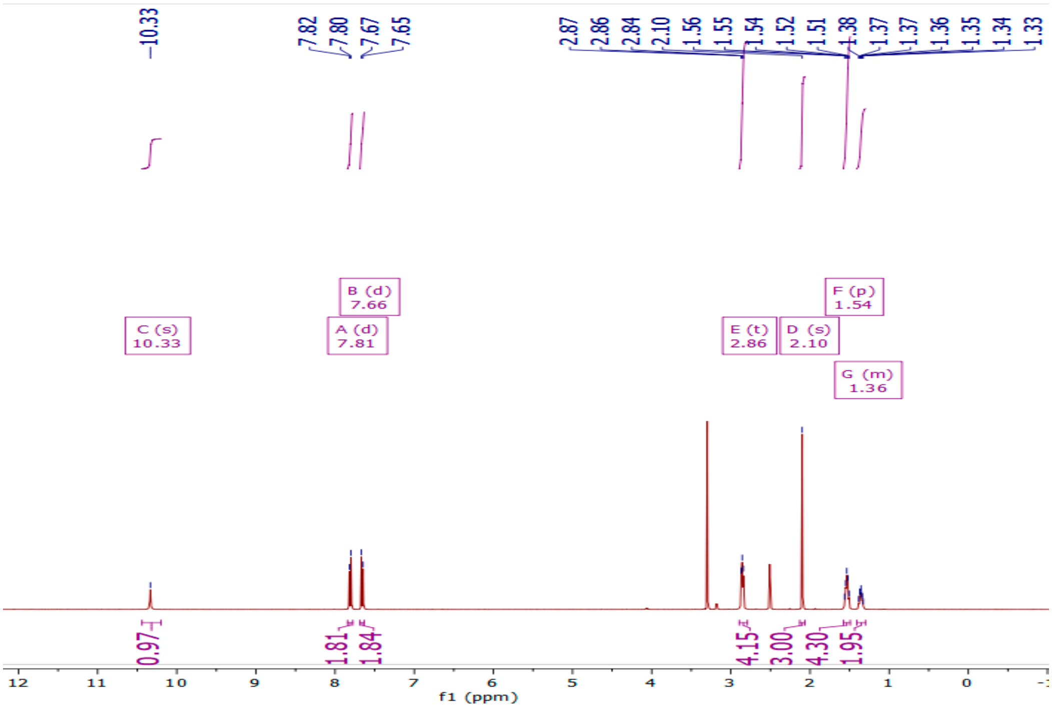

Figure S13. ^{1}H NMR spectrum of compound 2b

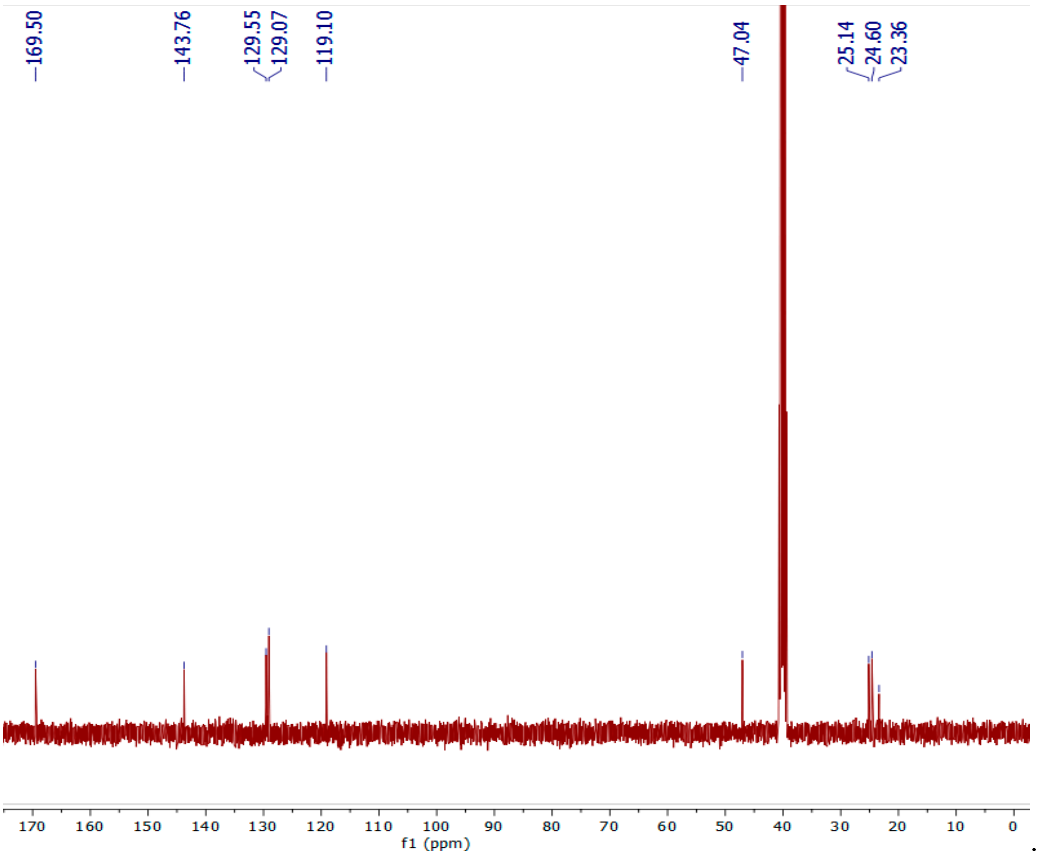

Figure S14. ^{13}CNMR spectrum of compound 2b

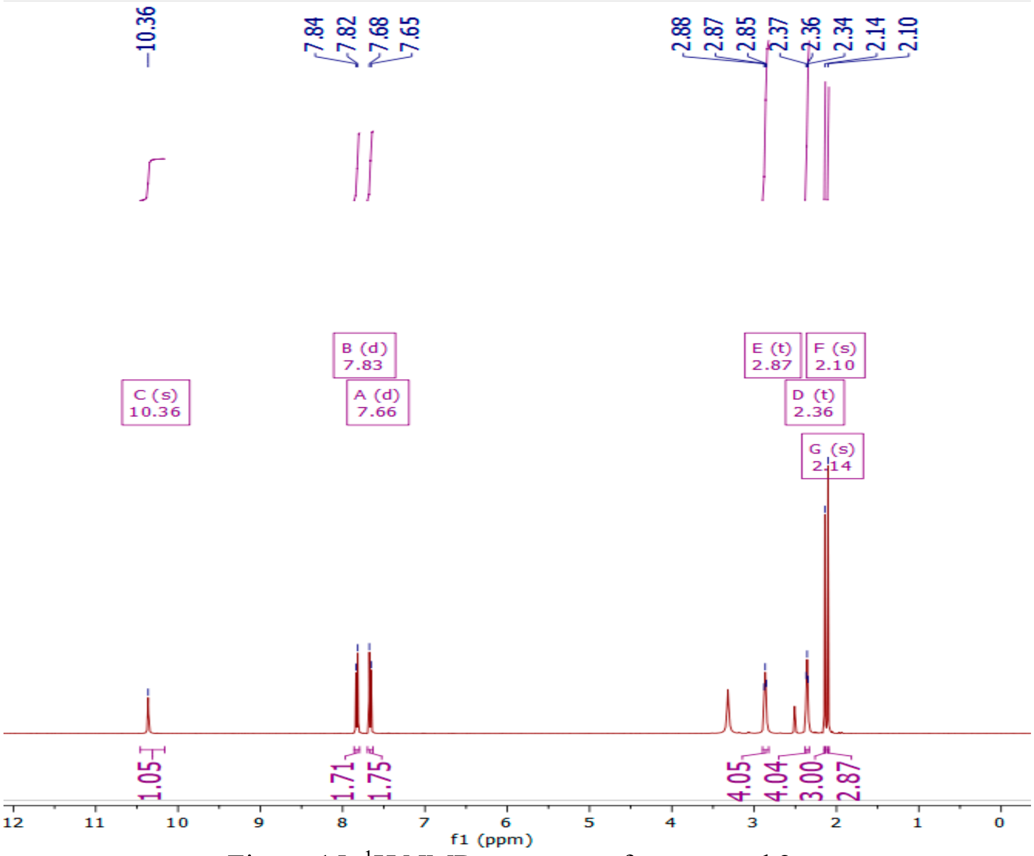

Figure 15. ^{1}H NMR spectrum of compound 2c

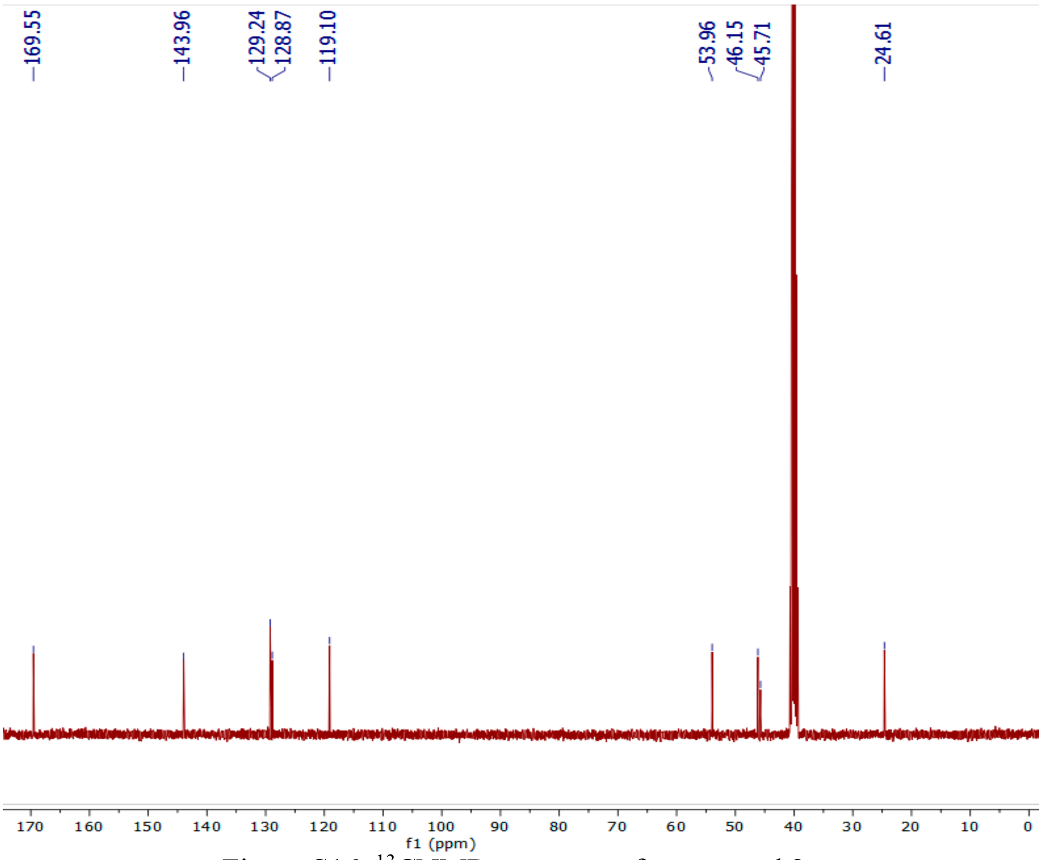

Figure S16. ^{13}CNMR spectrum of compound 2c

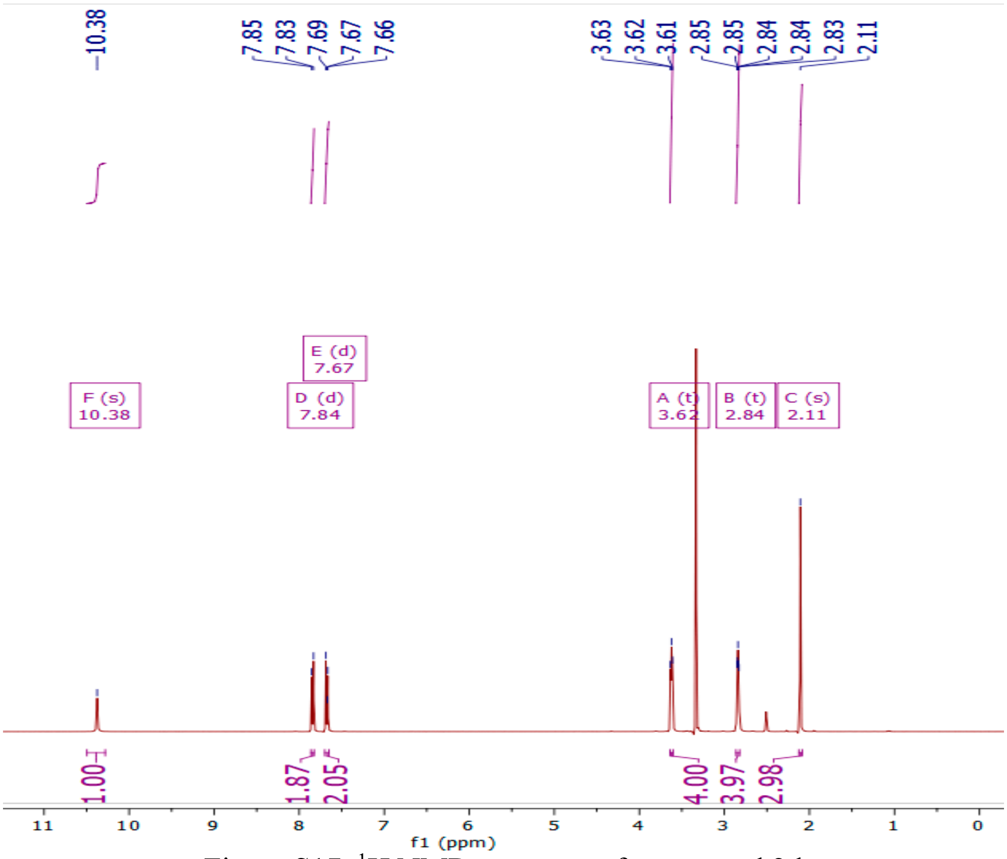

Figure S17. ^{1}H NMR spectrum of compound 2d

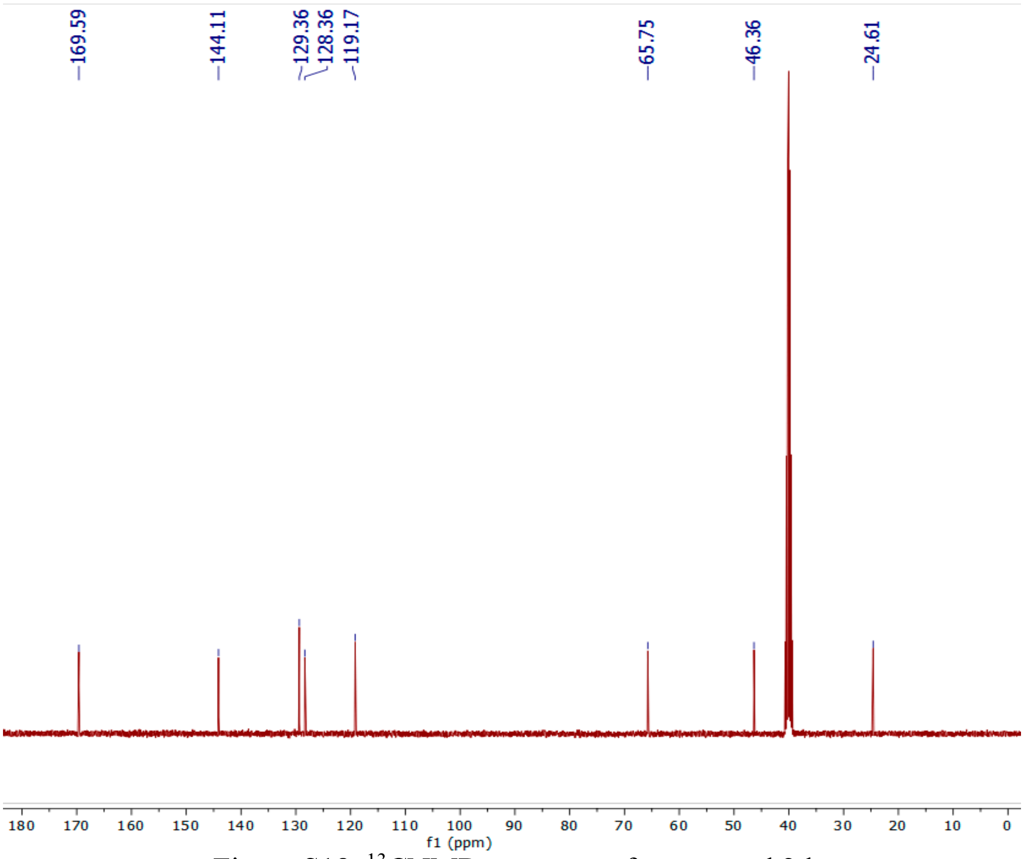

Figure S18. ^{13}CNMR spectrum of compound 2d

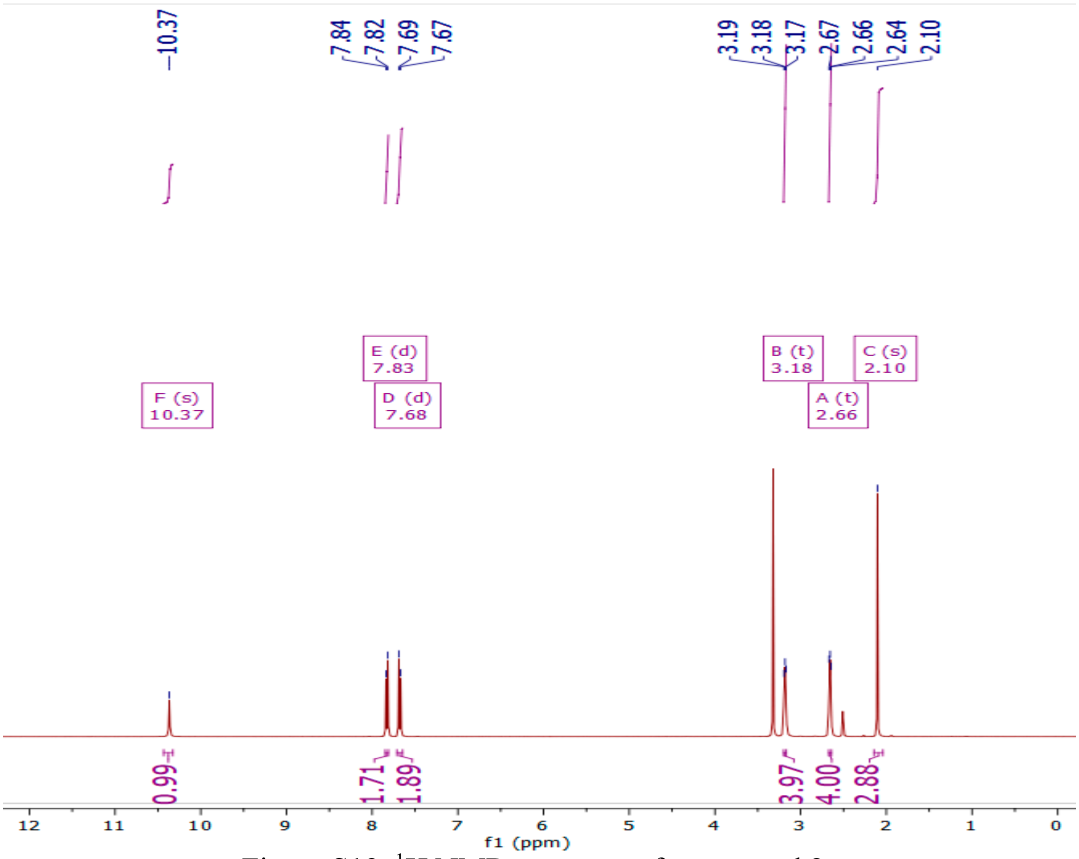

Figure S19. ^{1}H NMR spectrum of compound 2e

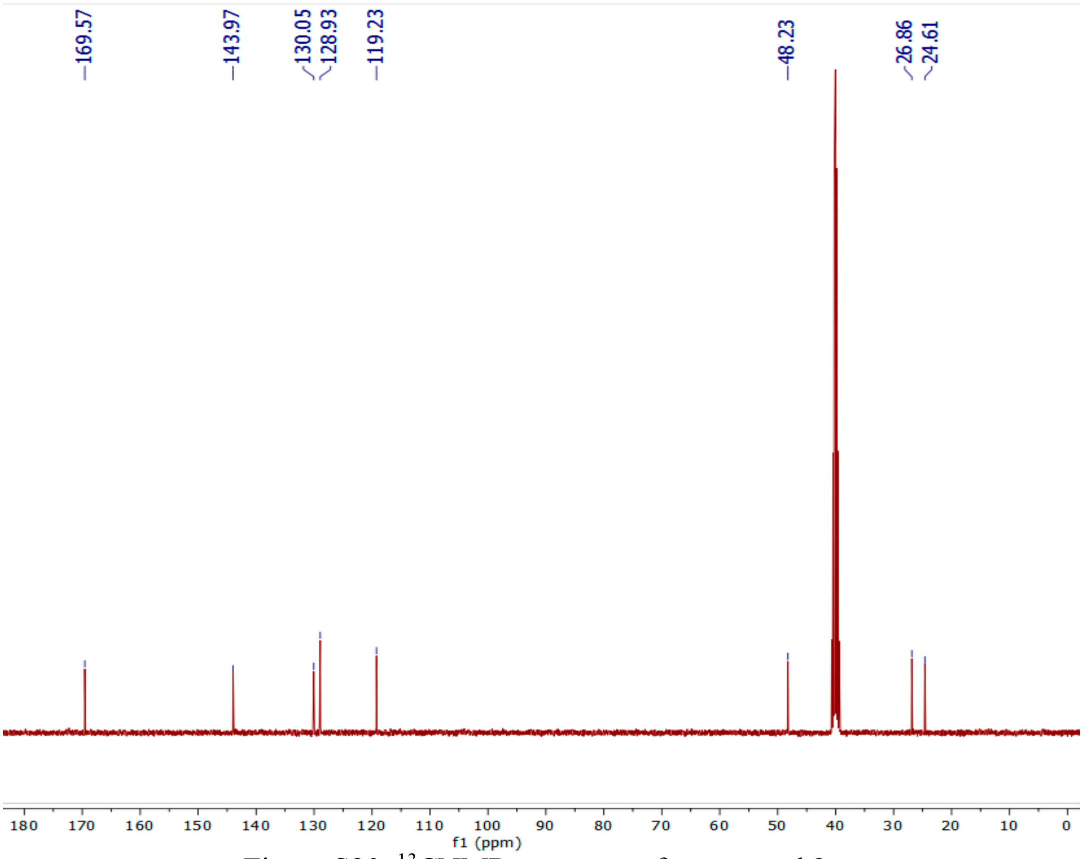

Figure S20. ^{13}CNMR spectrum of compound 2e

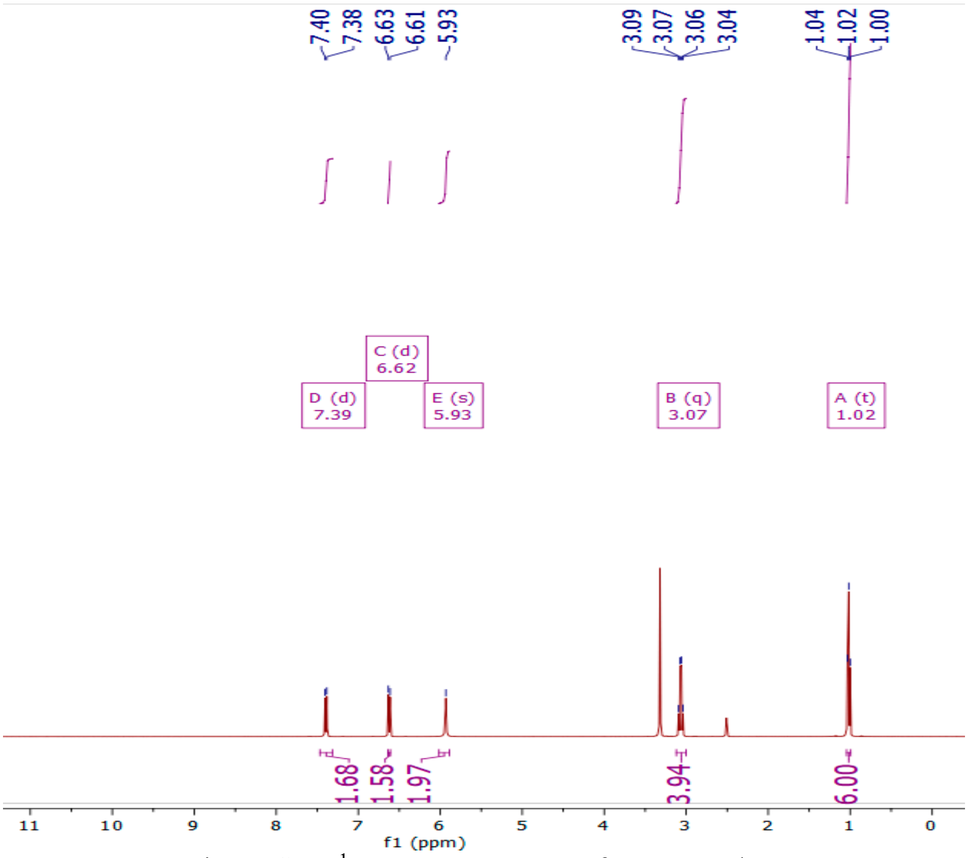

Figure S21. ^{1}H NMR spectrum of compound 3a

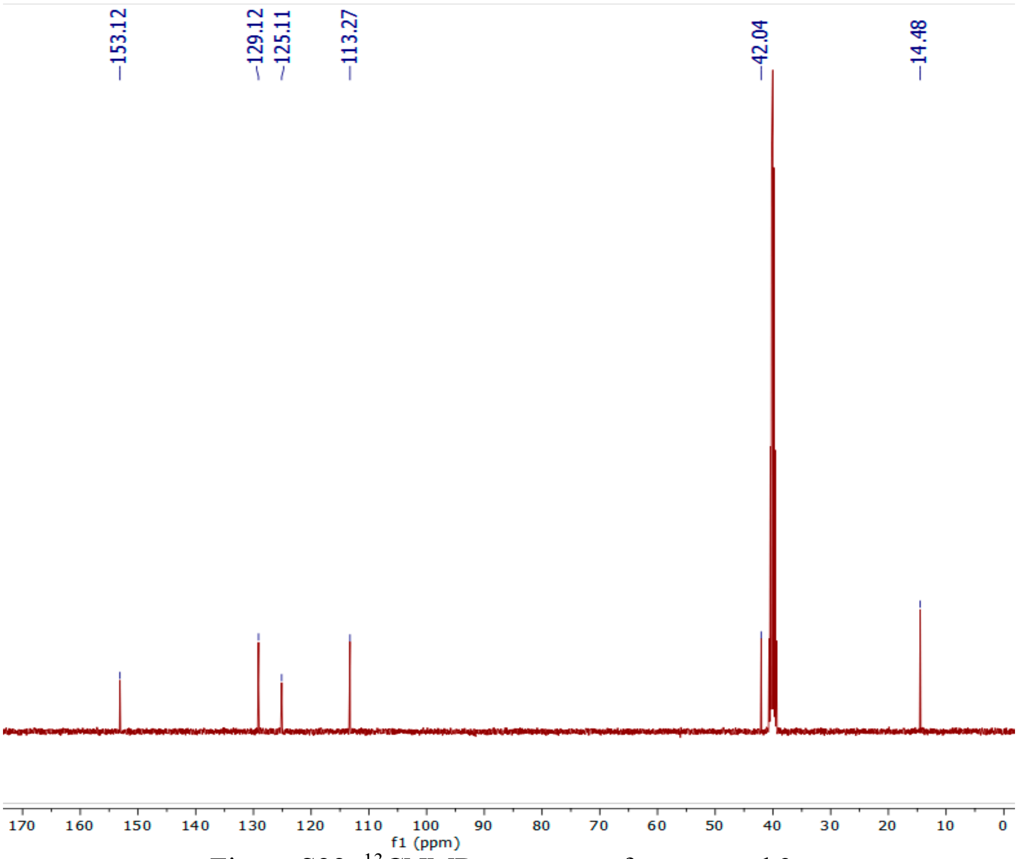

Figure S22. ^{13}CNMR spectrum of compound 3a

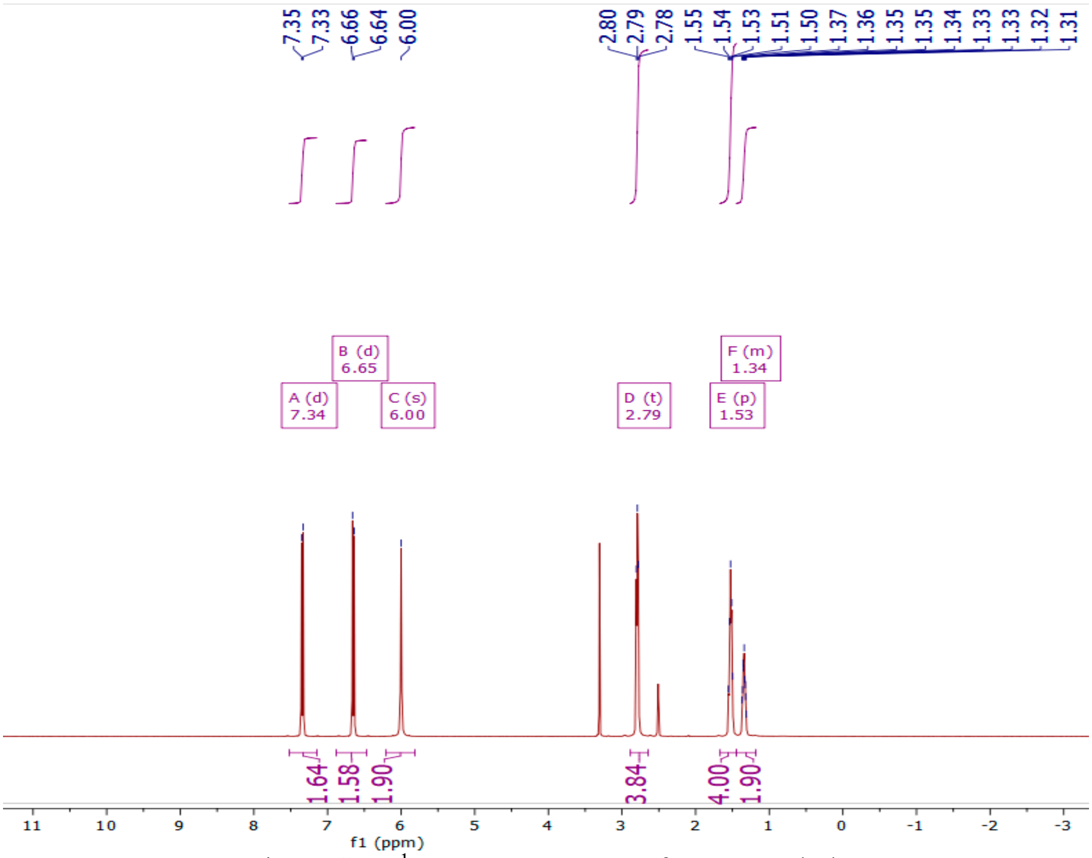

Figure S23. ^{1}H NMR spectrum of compound 3b

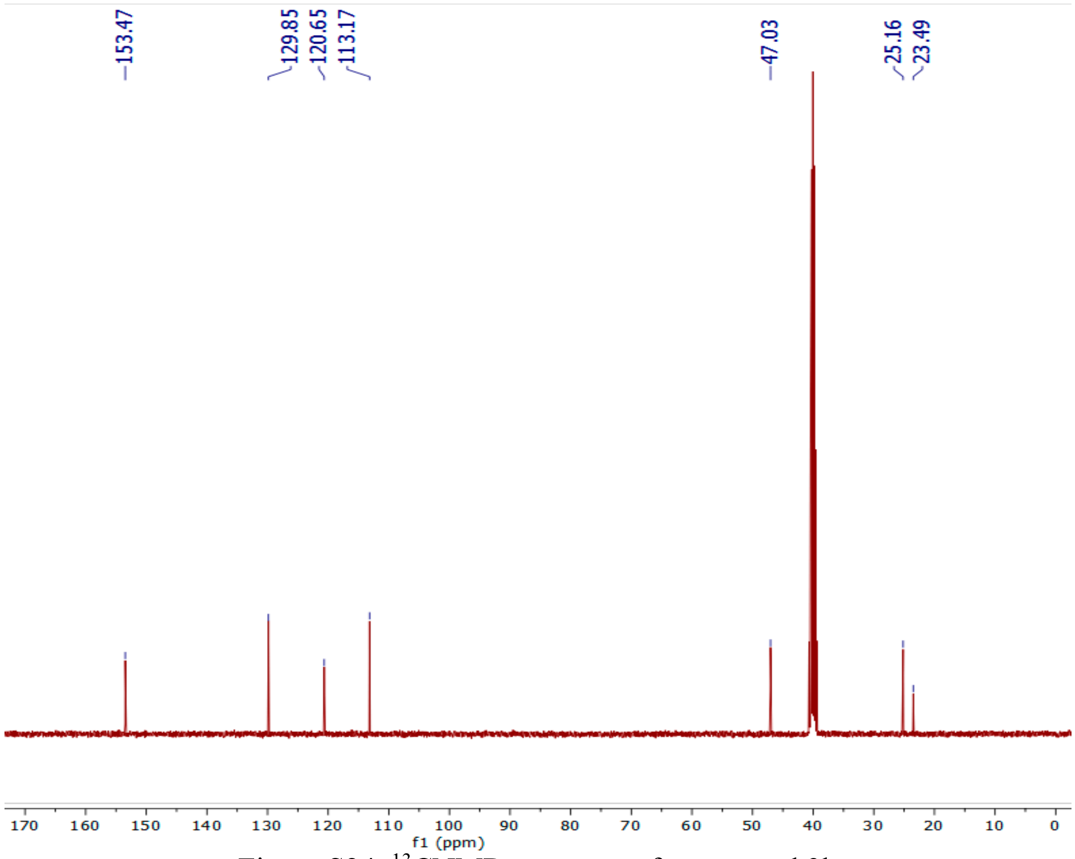

Figure S24. ^{13}CNMR spectrum of compound 3b

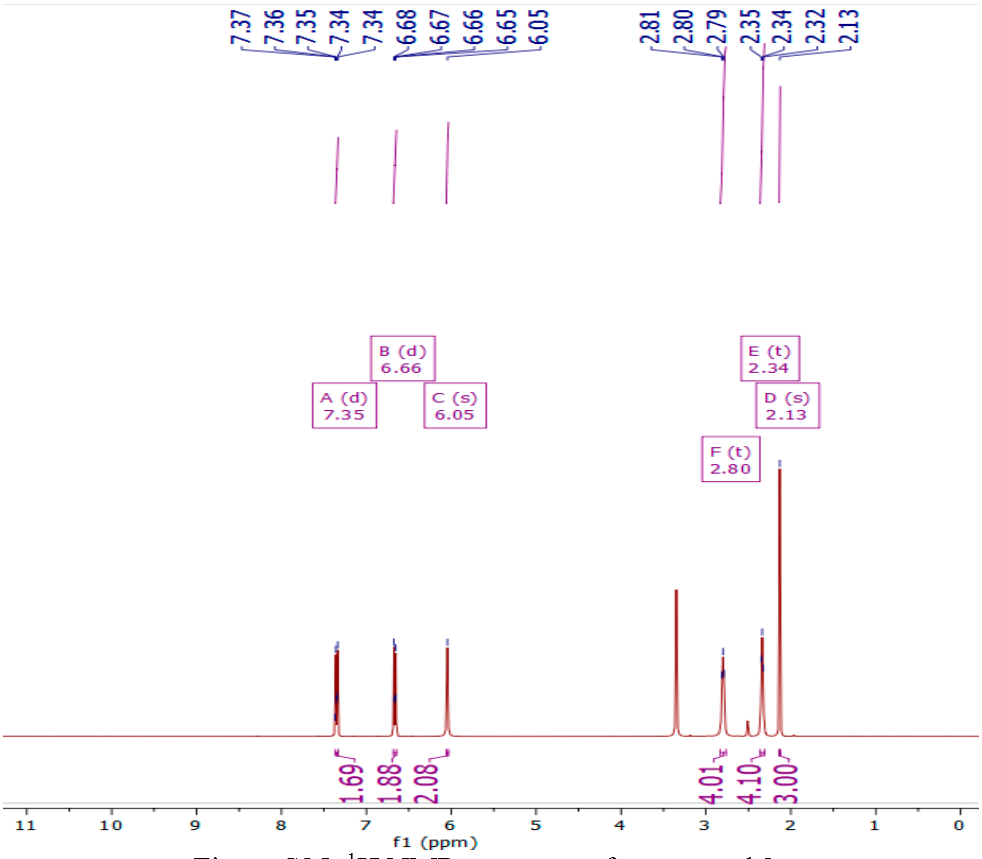

Figure S25. ^{1}H NMR spectrum of compound 3c

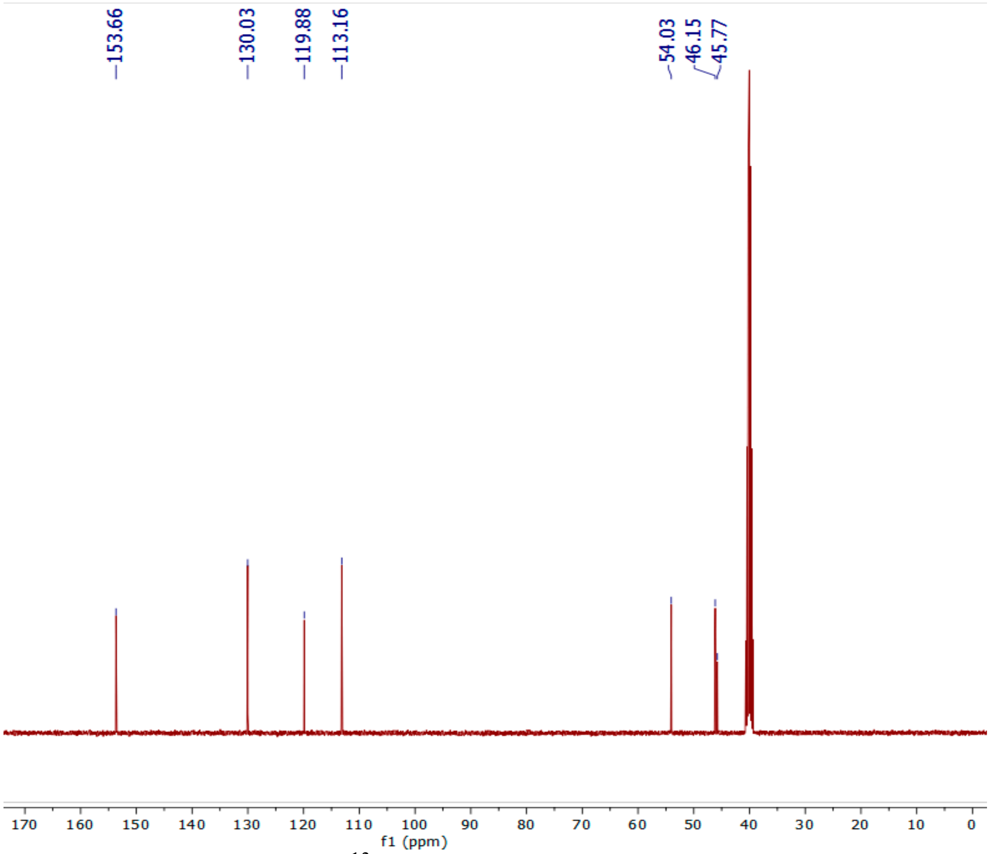

Figure S26. ^{13}CNMR spectrum of compound 3c

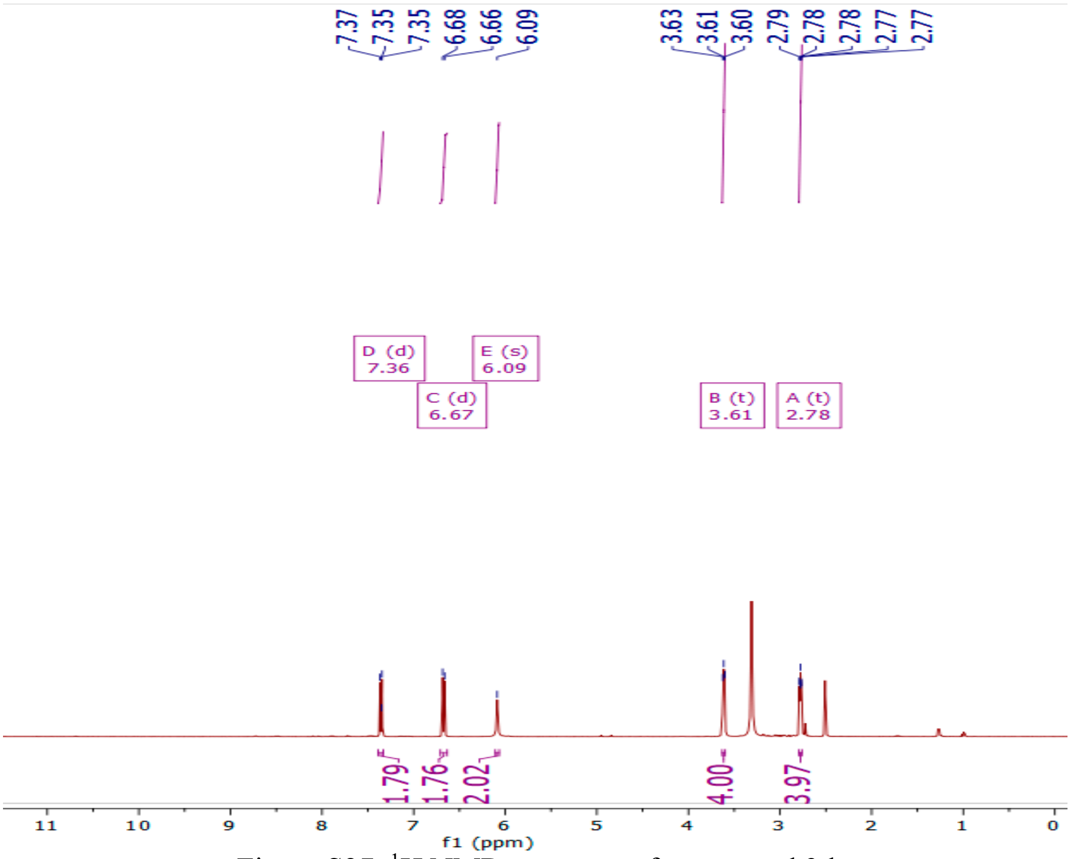

Figure S27. ^{1}H NMR spectrum of compound 3d

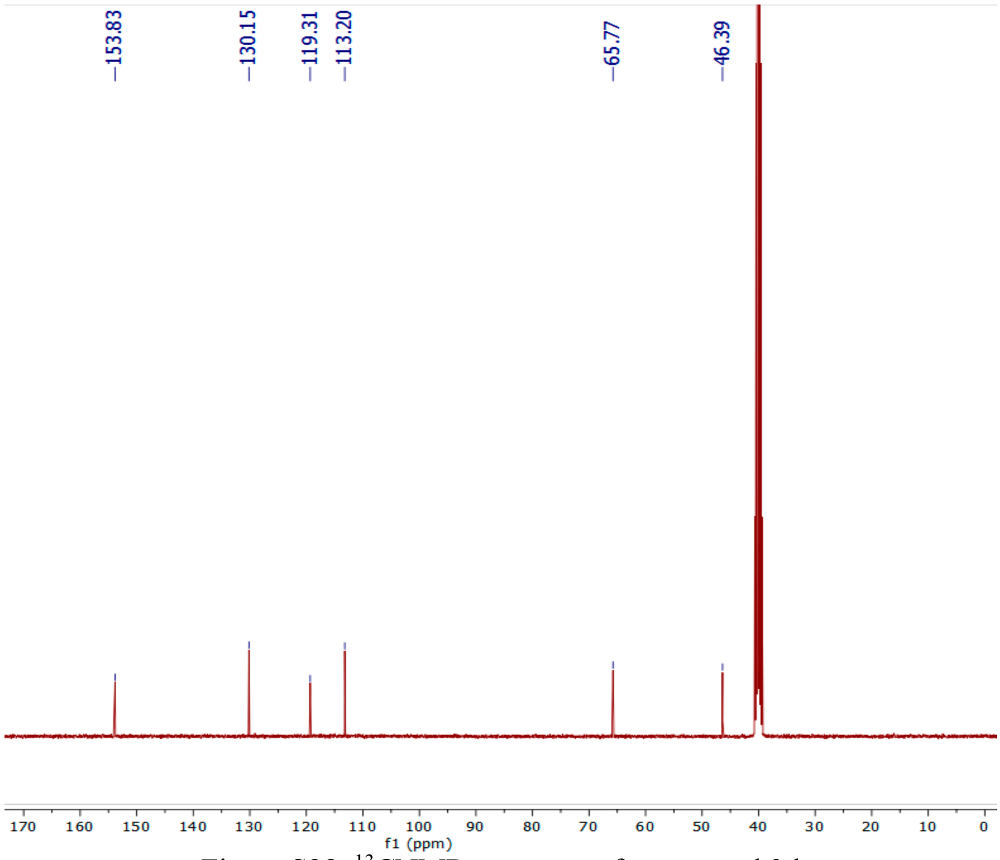

Figure S28. ^{13}CNMR spectrum of compound 3d

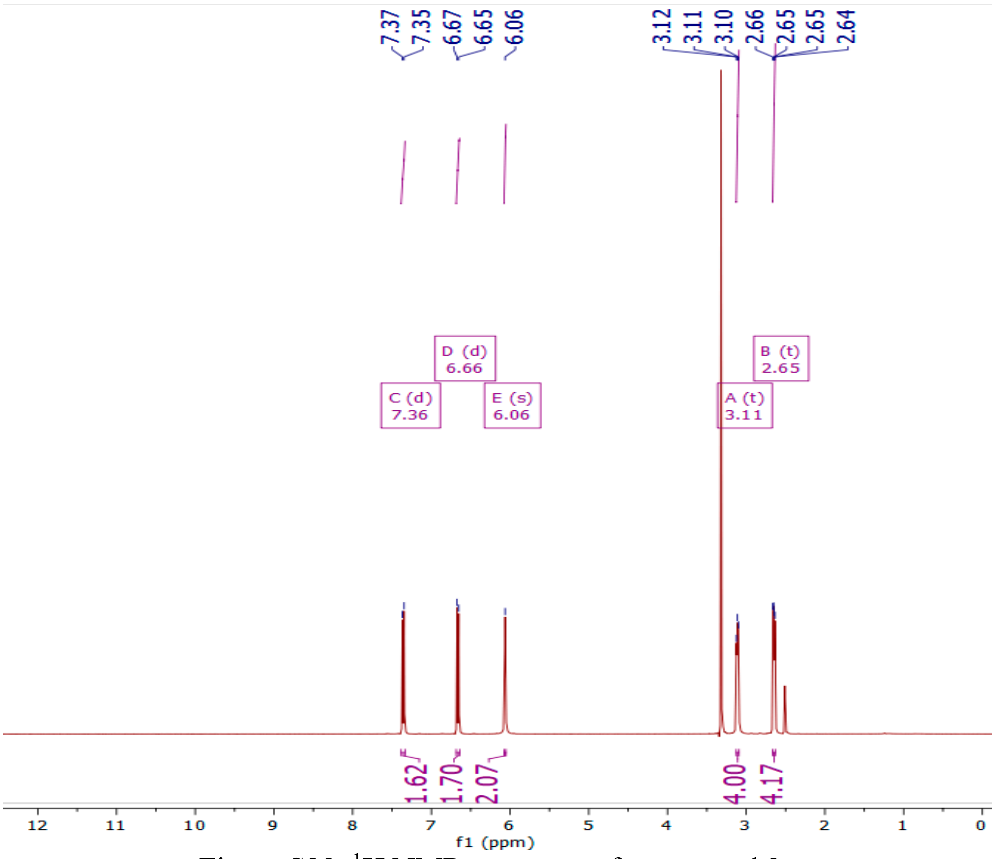

Figure S29. ^{1}H NMR spectrum of compound 3e

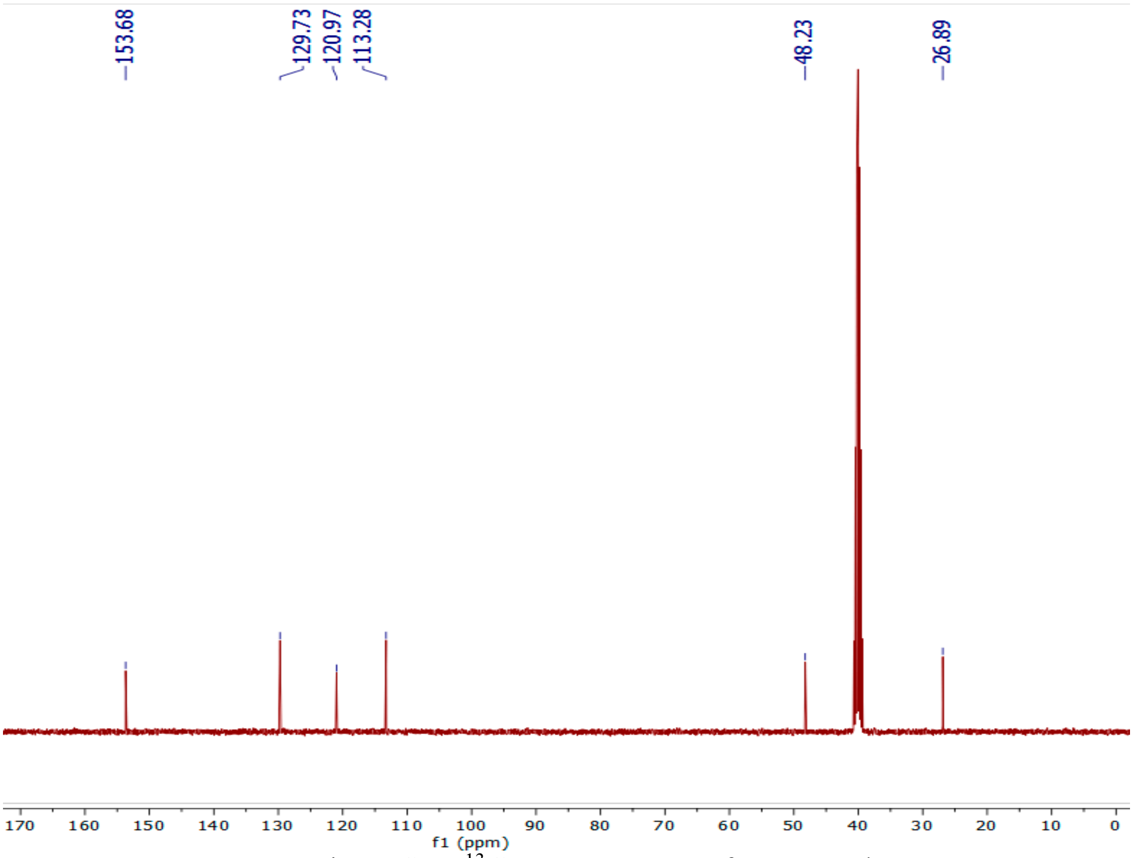

Figure S30. ^{13}CNMR spectrum of compound 3e

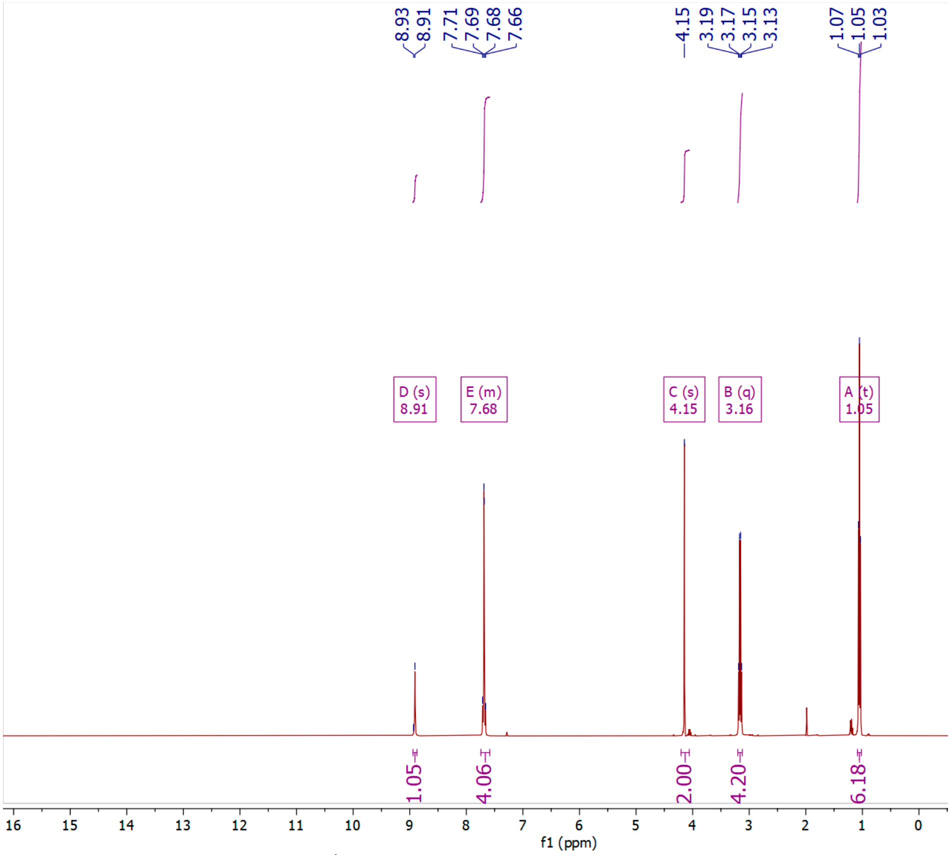

Figure S31. ^{1}H NMR spectrum of compound 4a

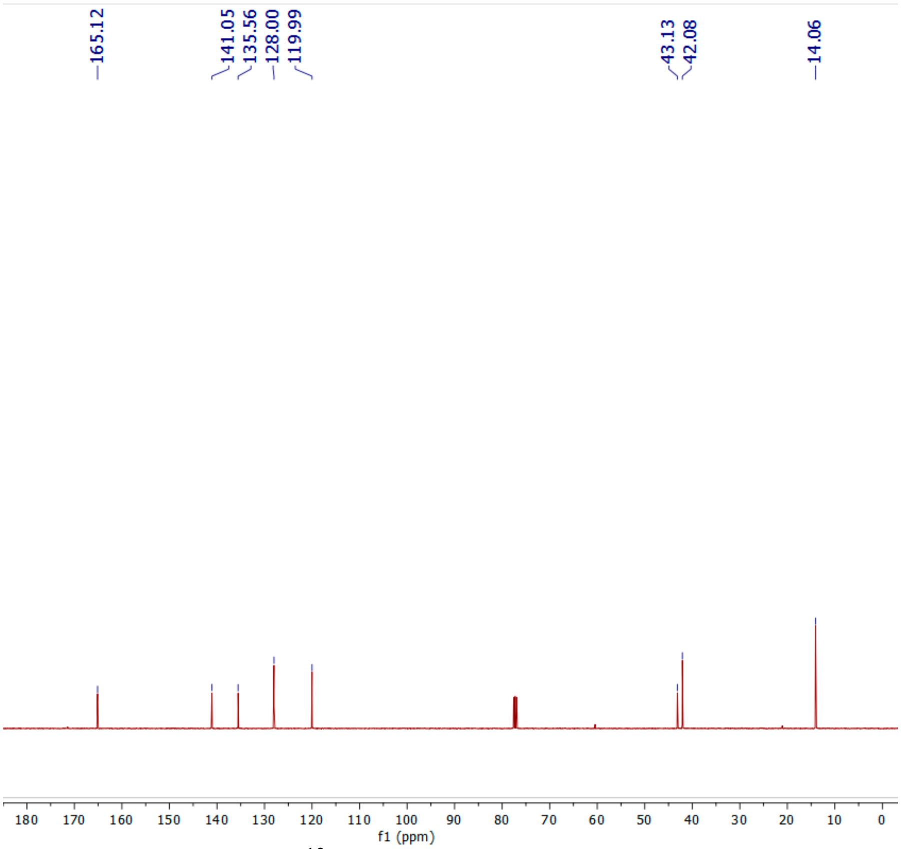

Figure S32. ^{13}CNMR spectrum of compound 4a

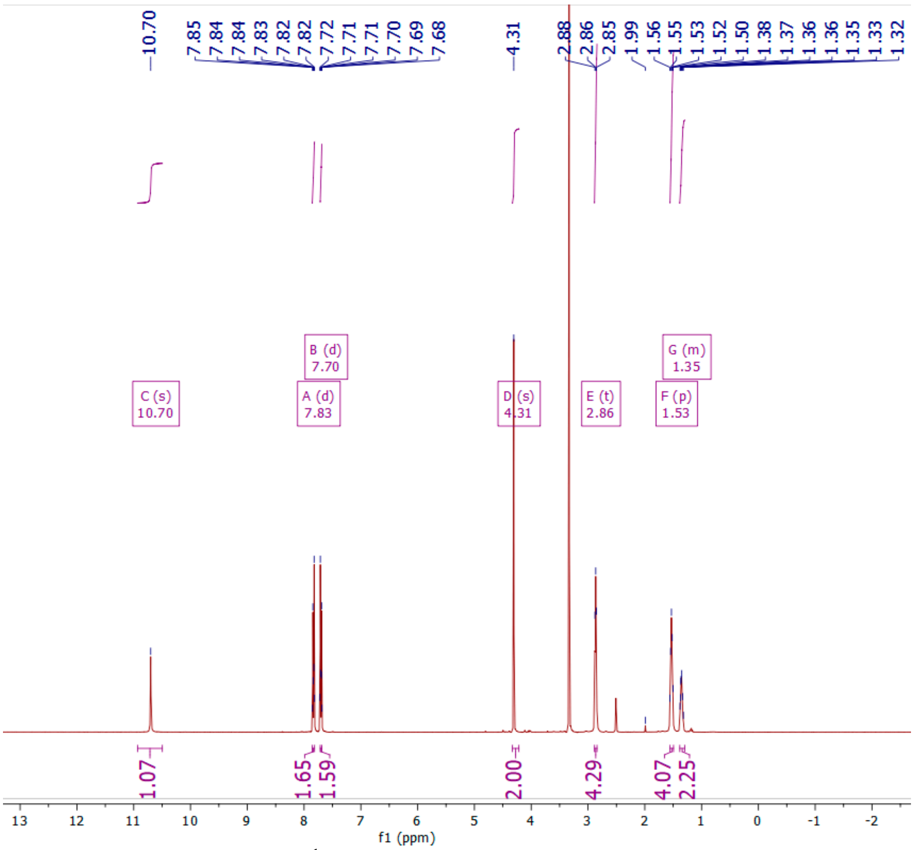

Figure S33. ^{1}H NMR spectrum of compound 4b

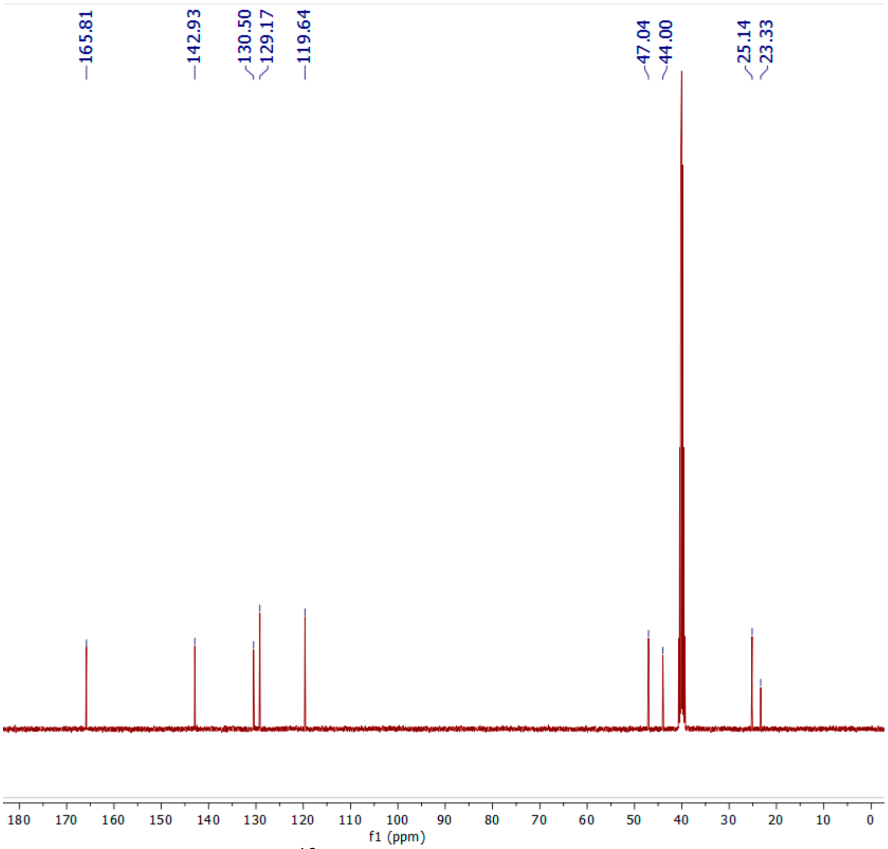

Figure S34. ^{13}CNMR spectrum of compound 4b

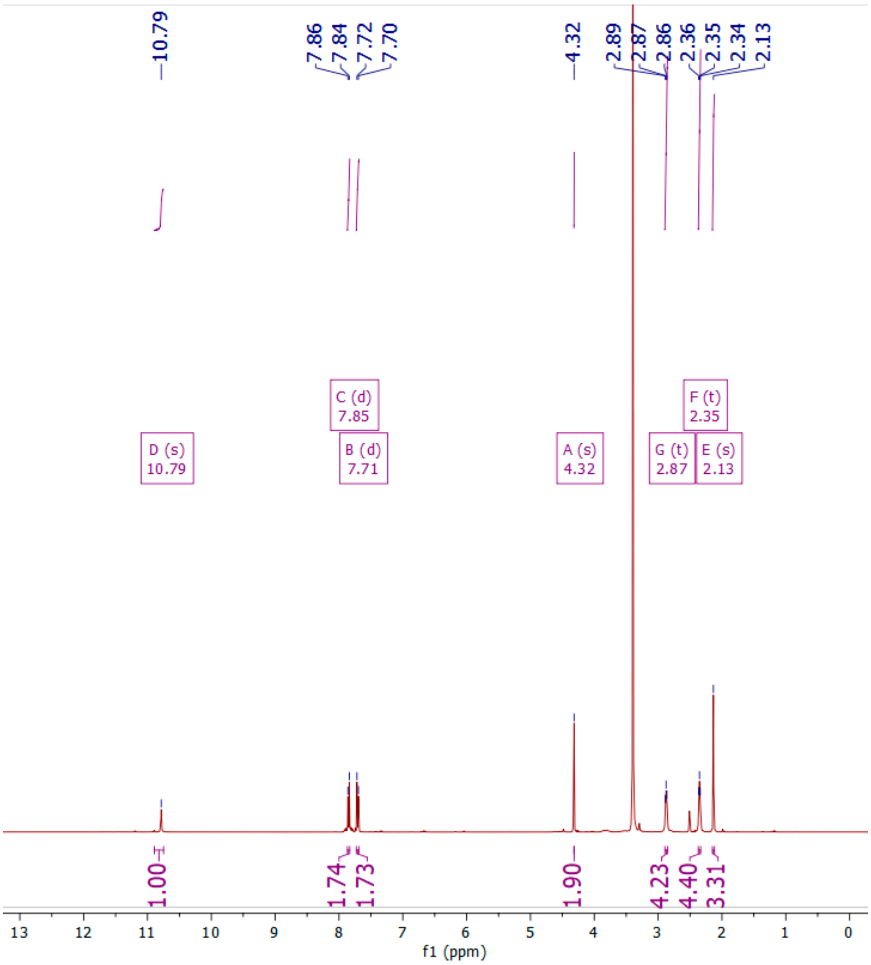

Figure S35. ^{1}H NMR spectrum of compound 4c

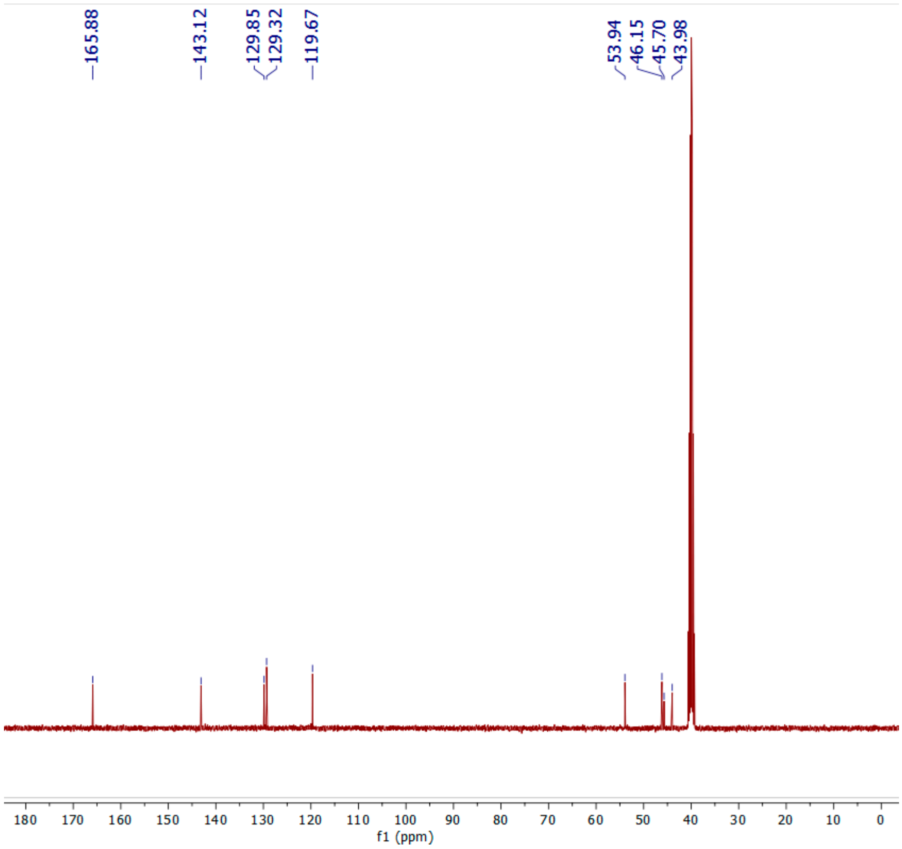

Figure S36. ^{13}CNMR spectrum of compound 4c

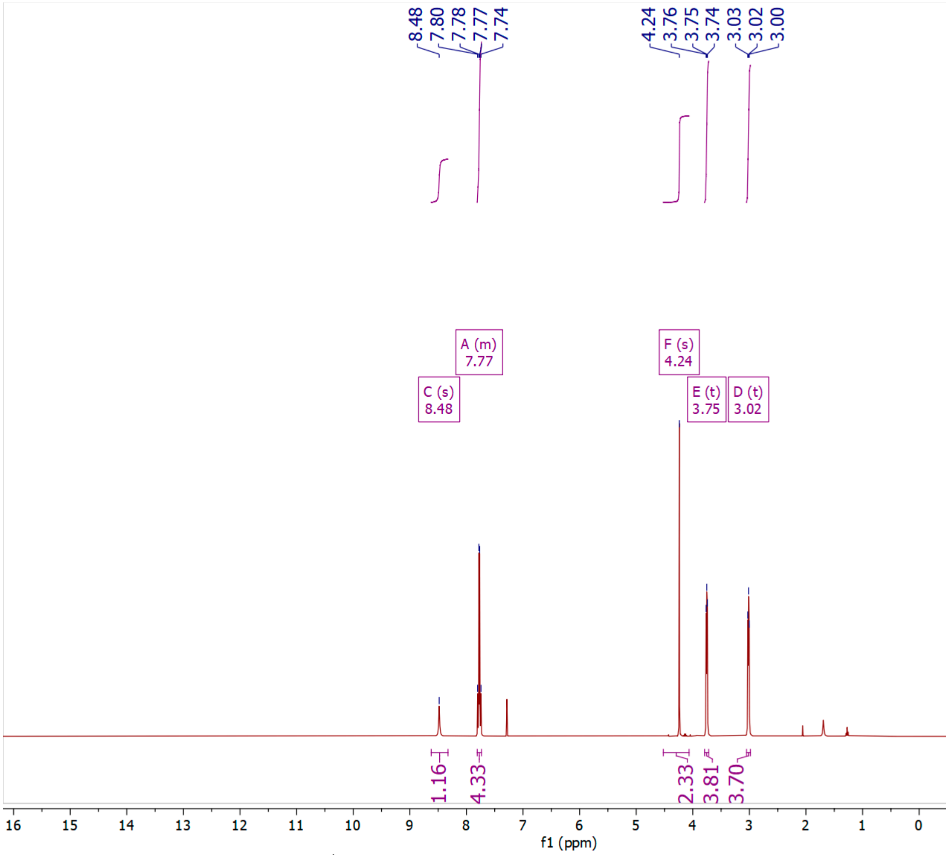

Figure S37. ^{1}H NMR spectrum of compound 4d

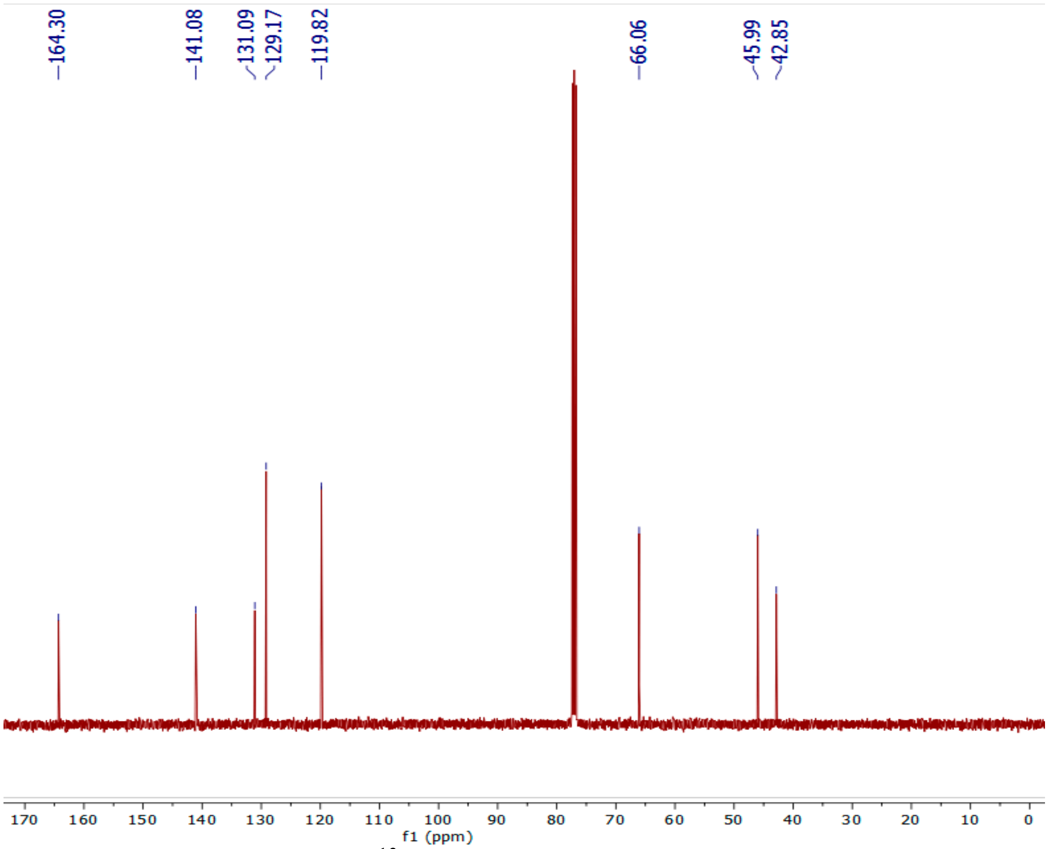

Figure S38. ^{13}CNMR spectrum of compound 4d

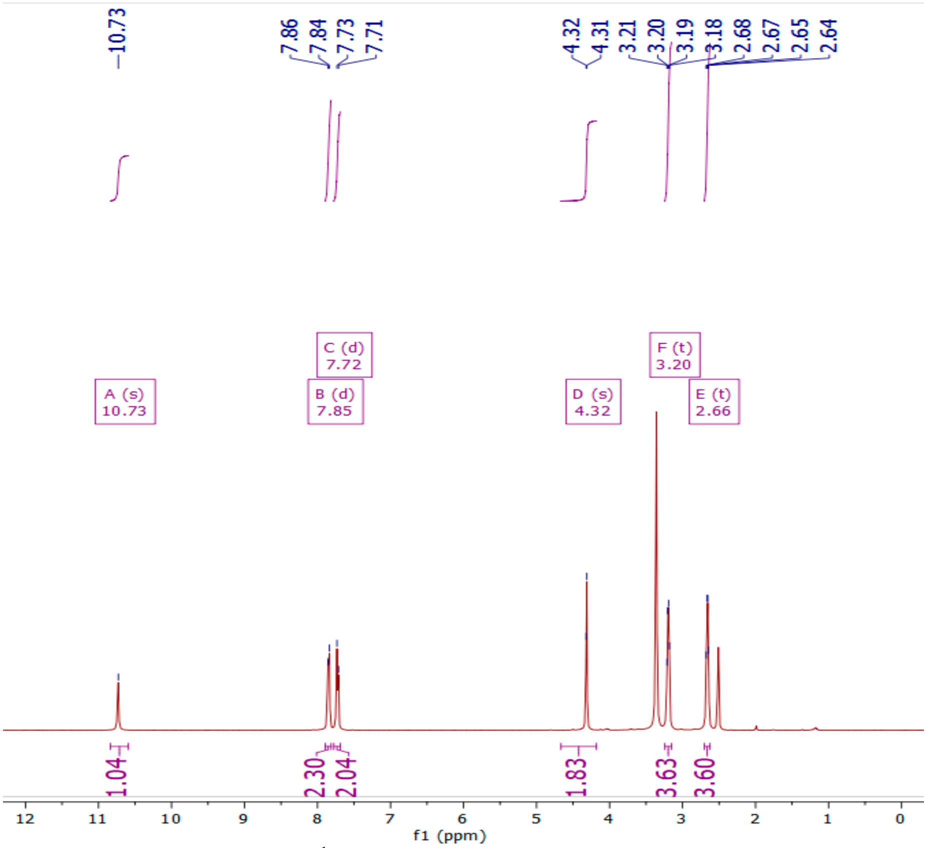

Figure S39. ^{1}H NMR spectrum of compound 4e

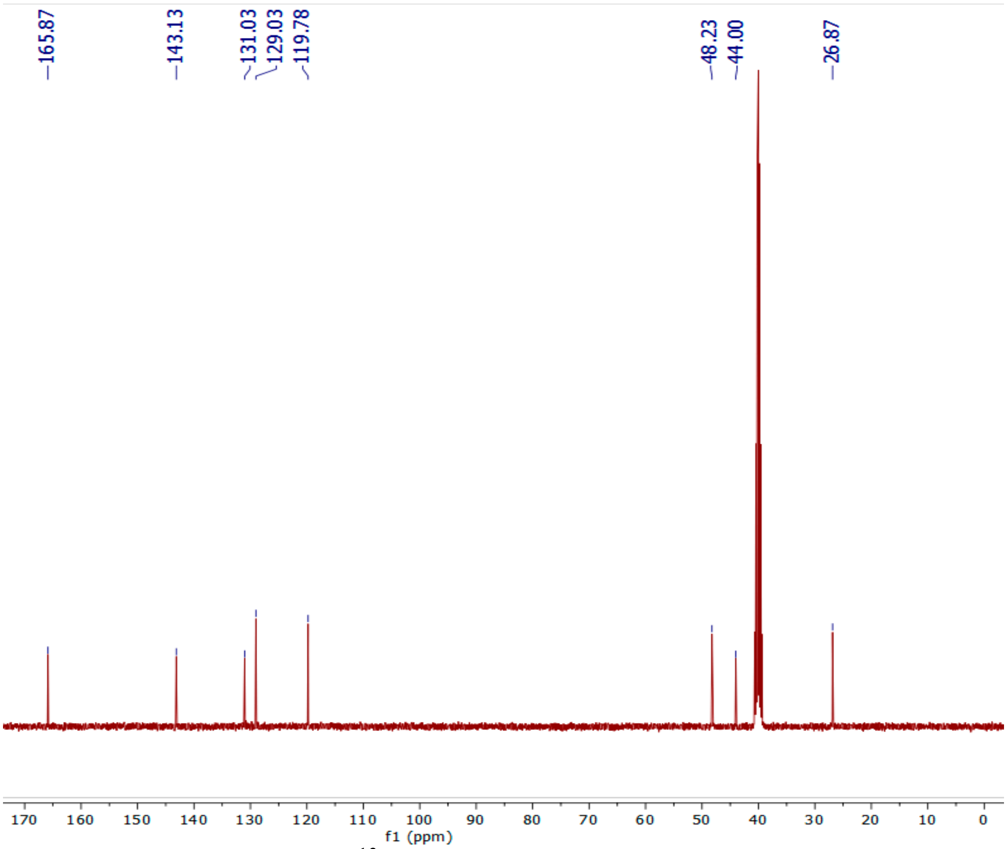

Figure S40. ^{13}CNMR spectrum of compound 4e

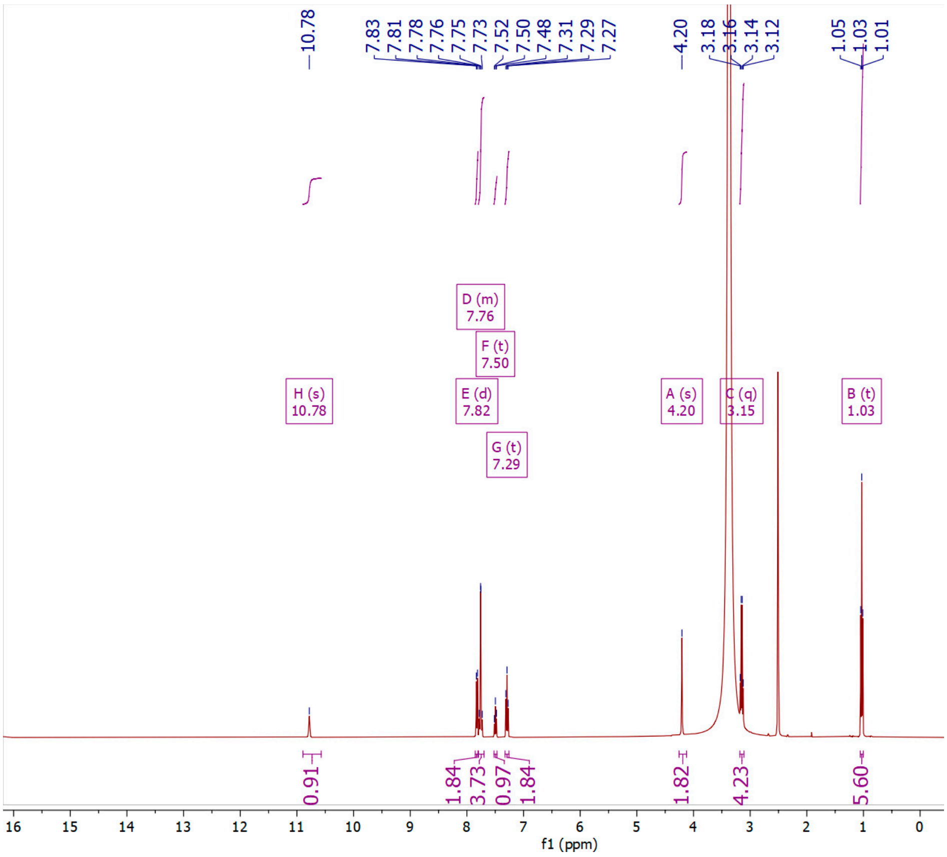

Figure S41. ¹H NMR spectrum of compound M1

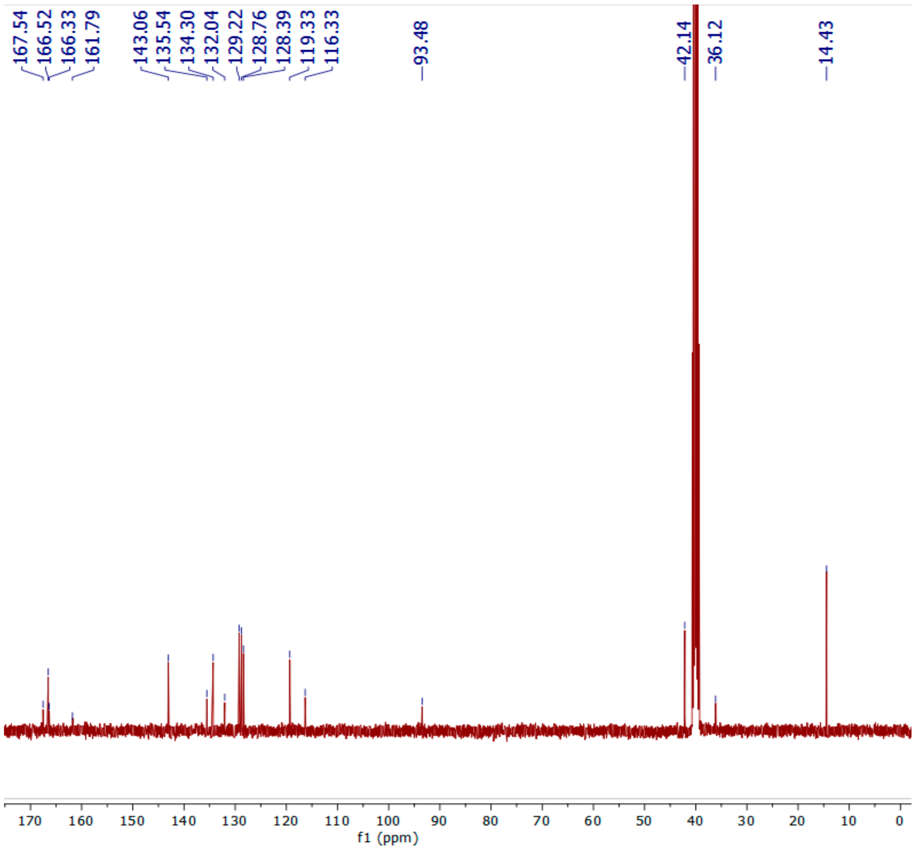

Figure S42. ^{13}C NMR spectrum of compound M1

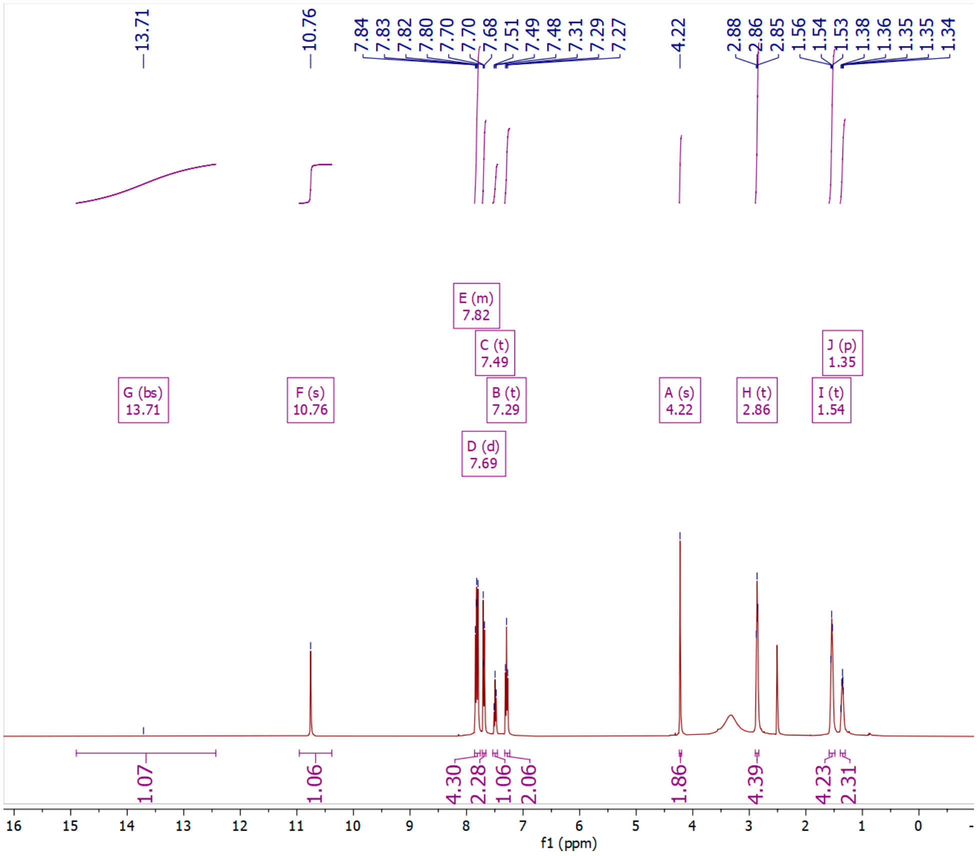

Figure S43. ^{1}H NMR spectrum of compound M2

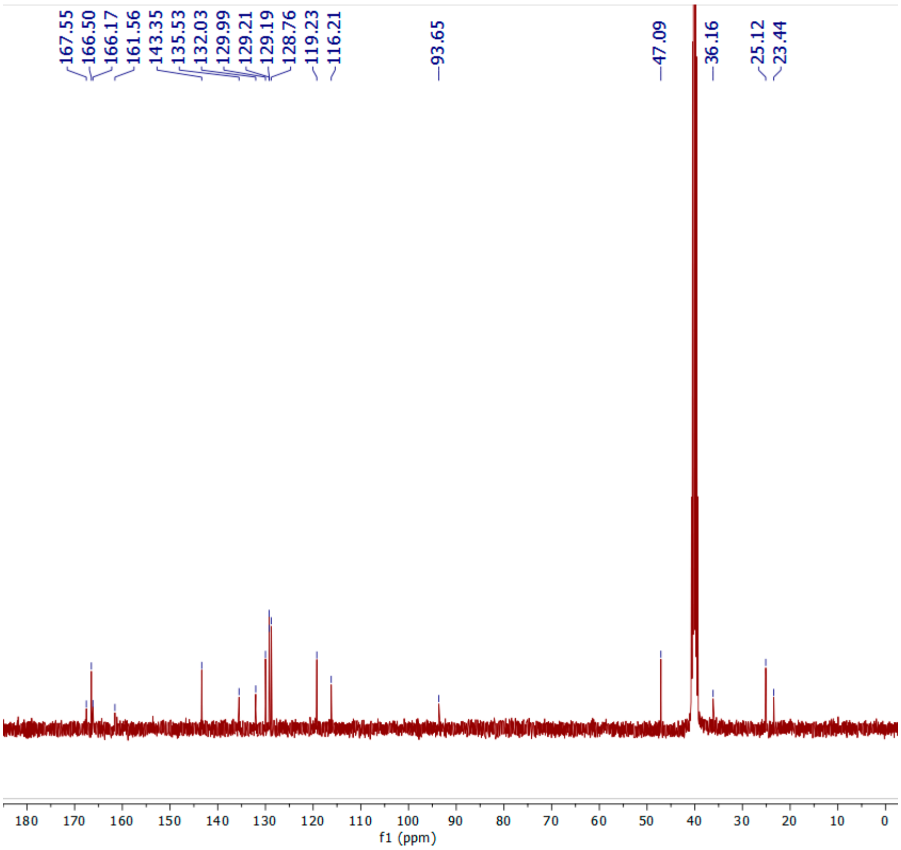

Figure S44. ^{13}C NMR spectrum of compound M2

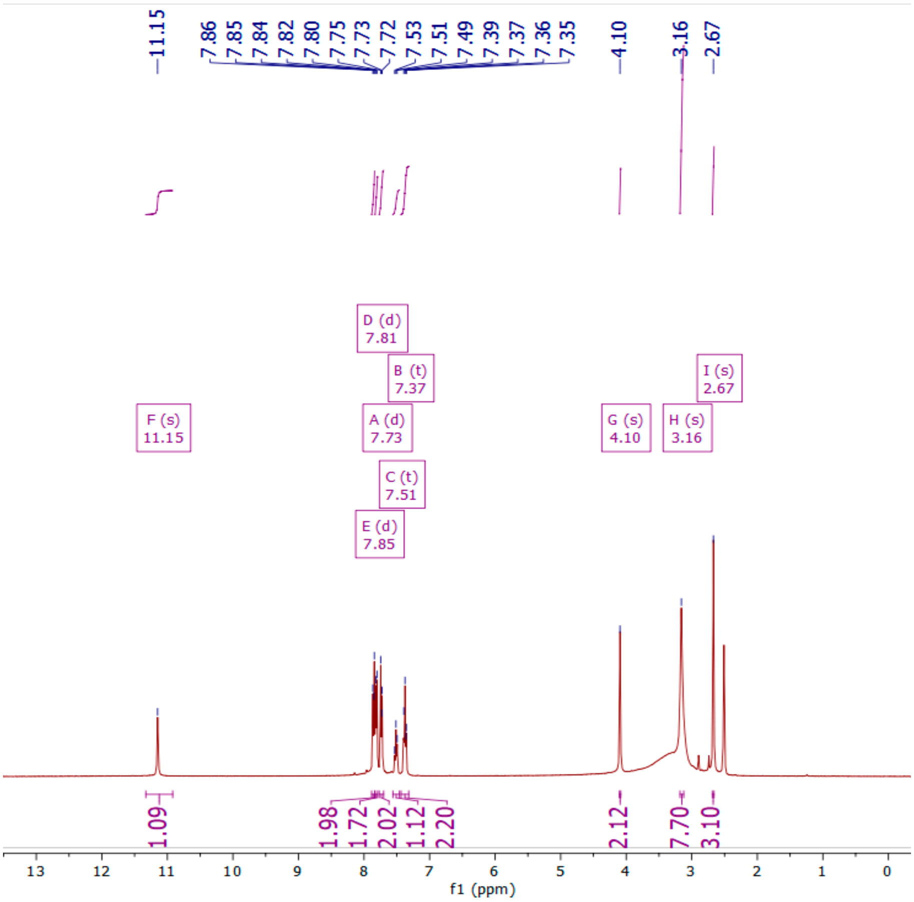

Figure S45. ^{1}H NMR spectrum of compound M3

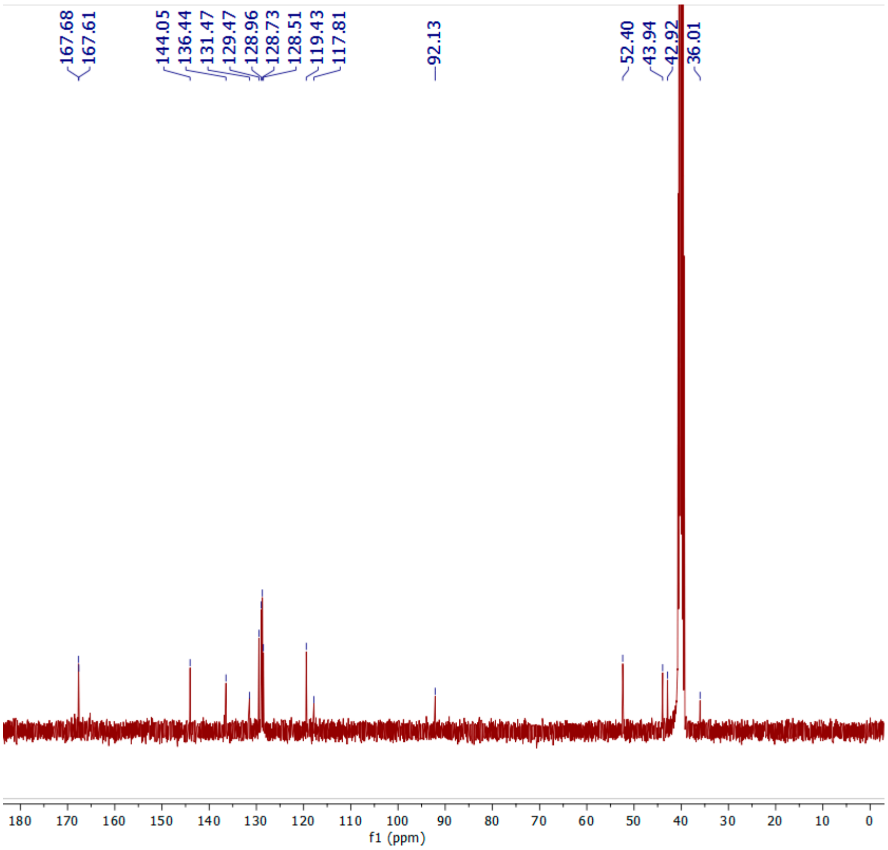

Figure S46. ^{13}C NMR spectrum of compound M3

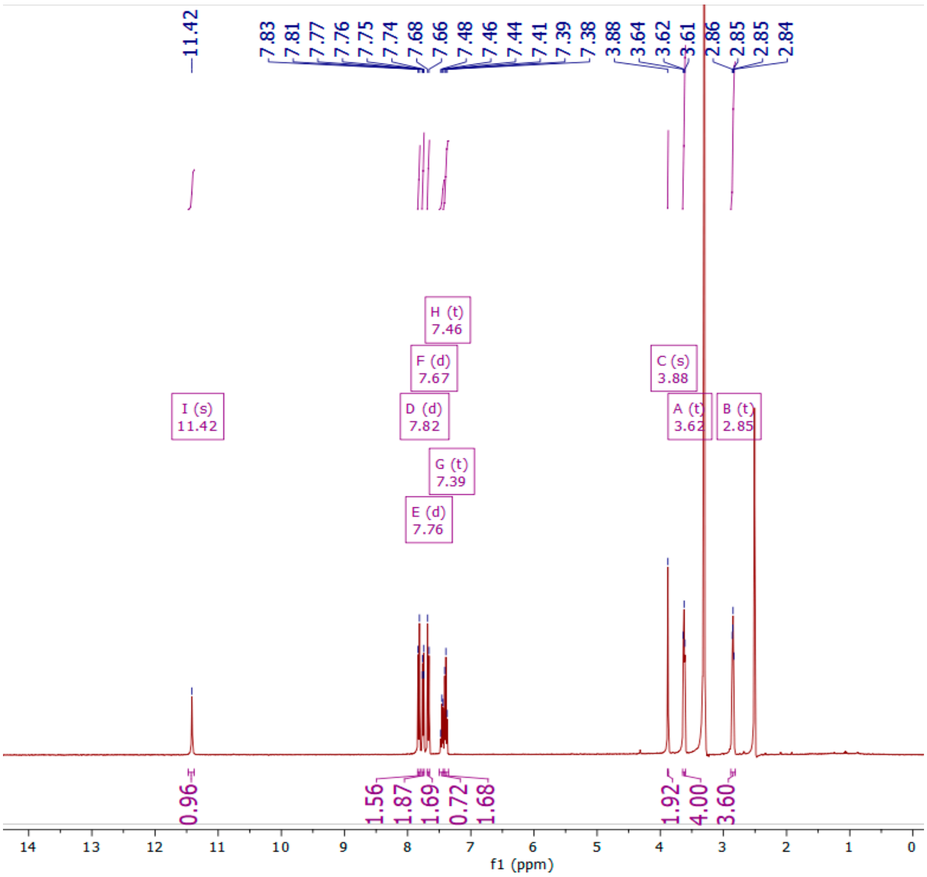

Figure S47. ^{1}H NMR spectrum of compound M4

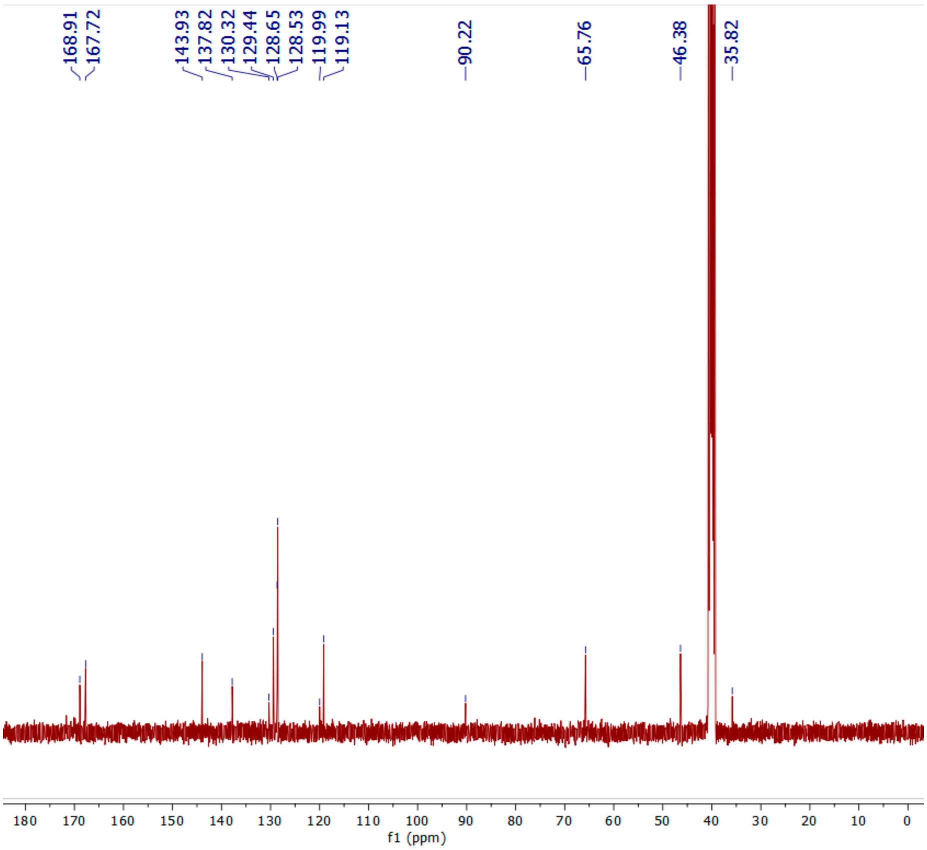

Figure S48. ^{13}C NMR spectrum of compound M4

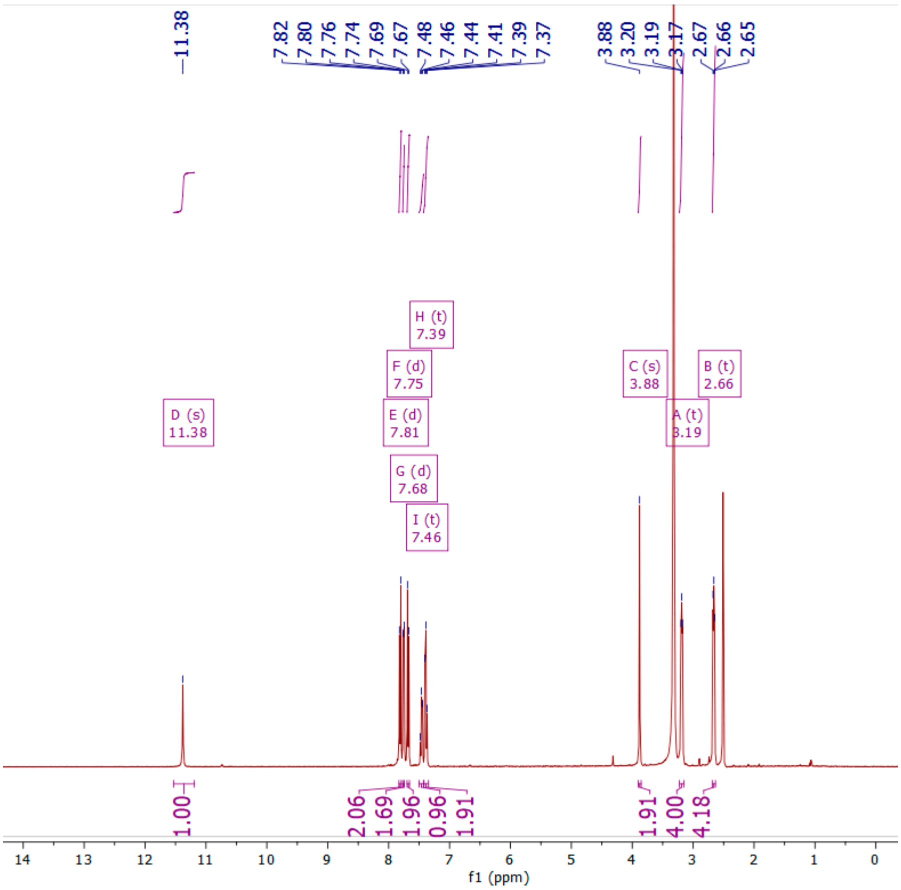

Figure S49. ^{1}H NMR spectrum of compound M5

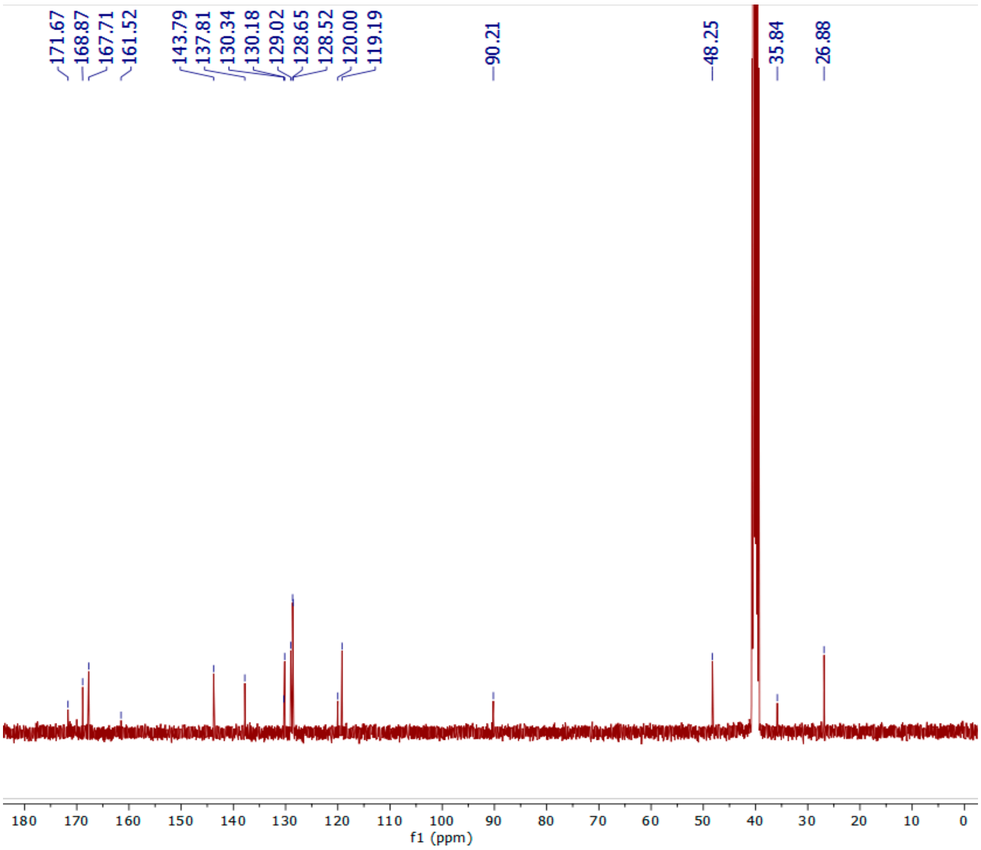

Figure S50. ^{13}C NMR spectrum of compound M5

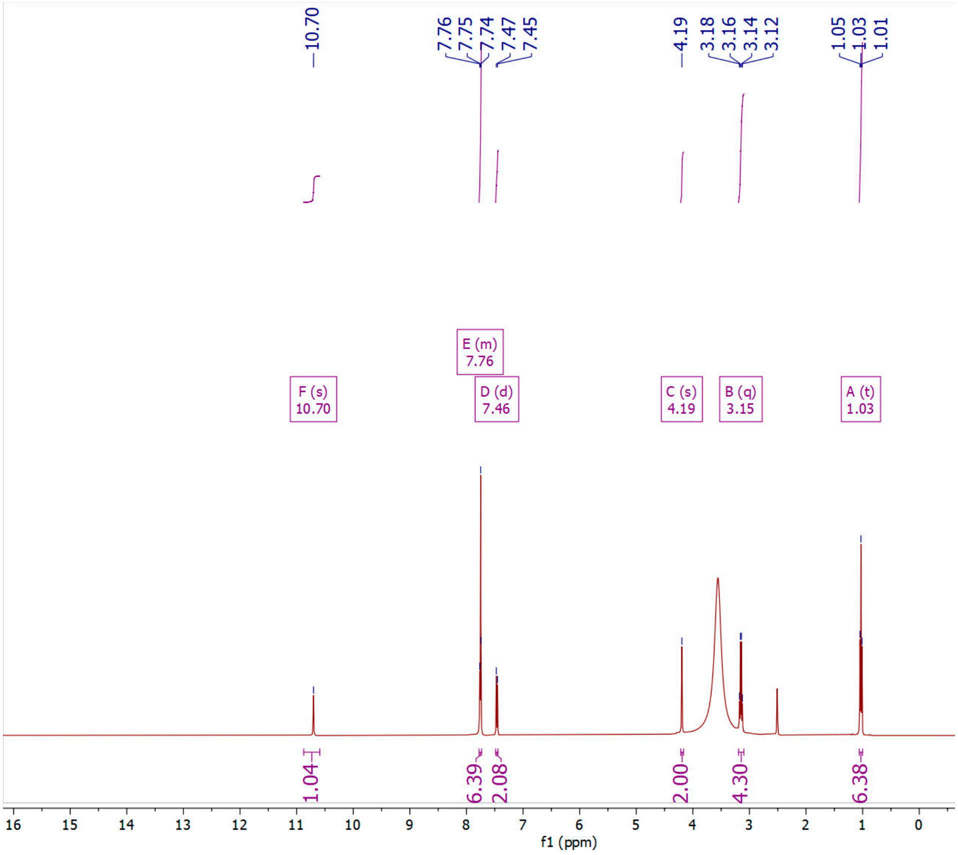

Figure S51. ^{1}H NMR spectrum of compound M6

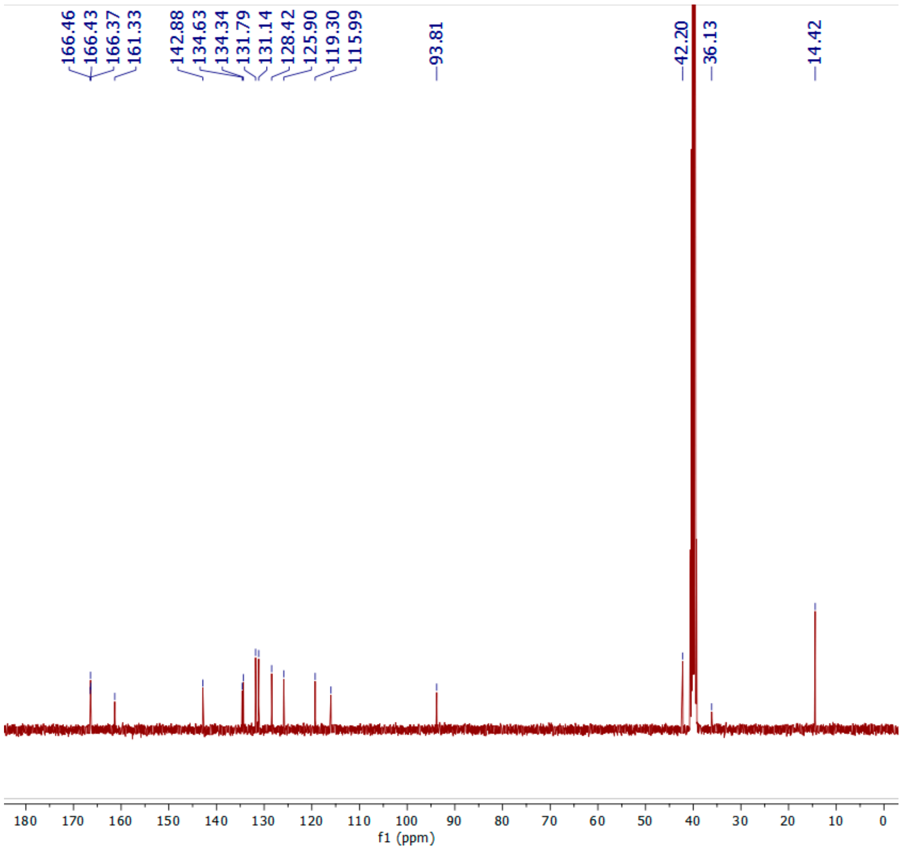

Figure S52. ^{13}C NMR spectrum of compound M6

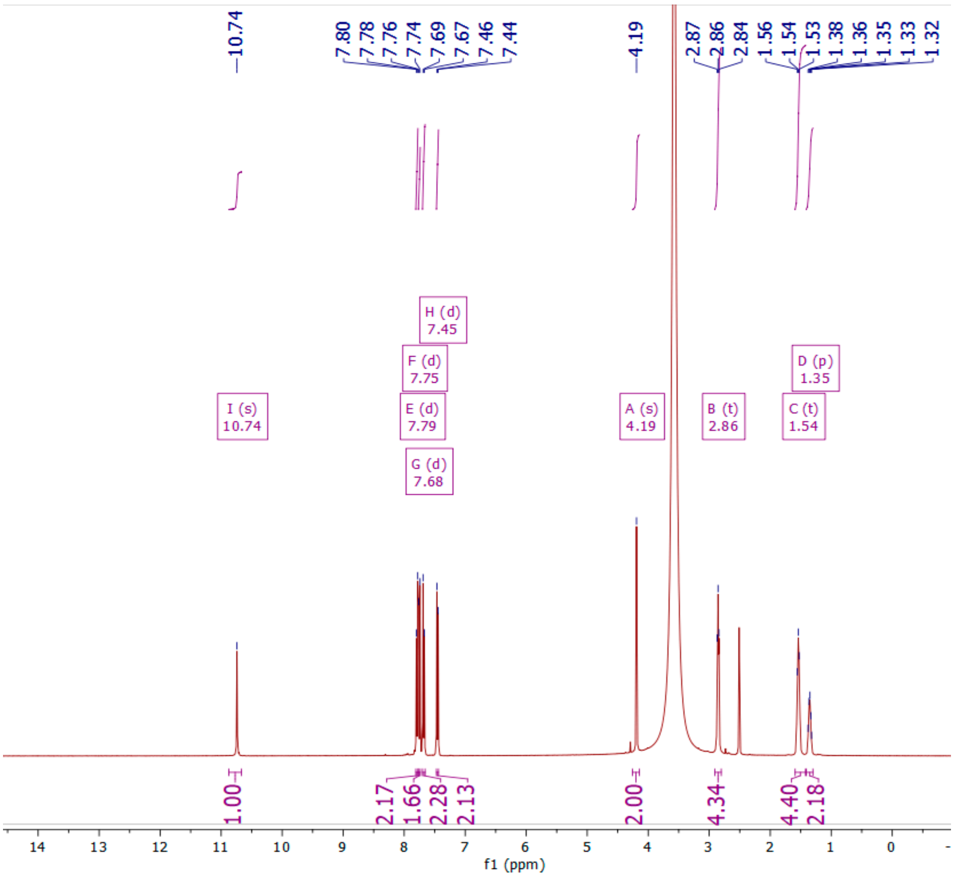

Figure S53. ^{1}H NMR spectrum of compound M7

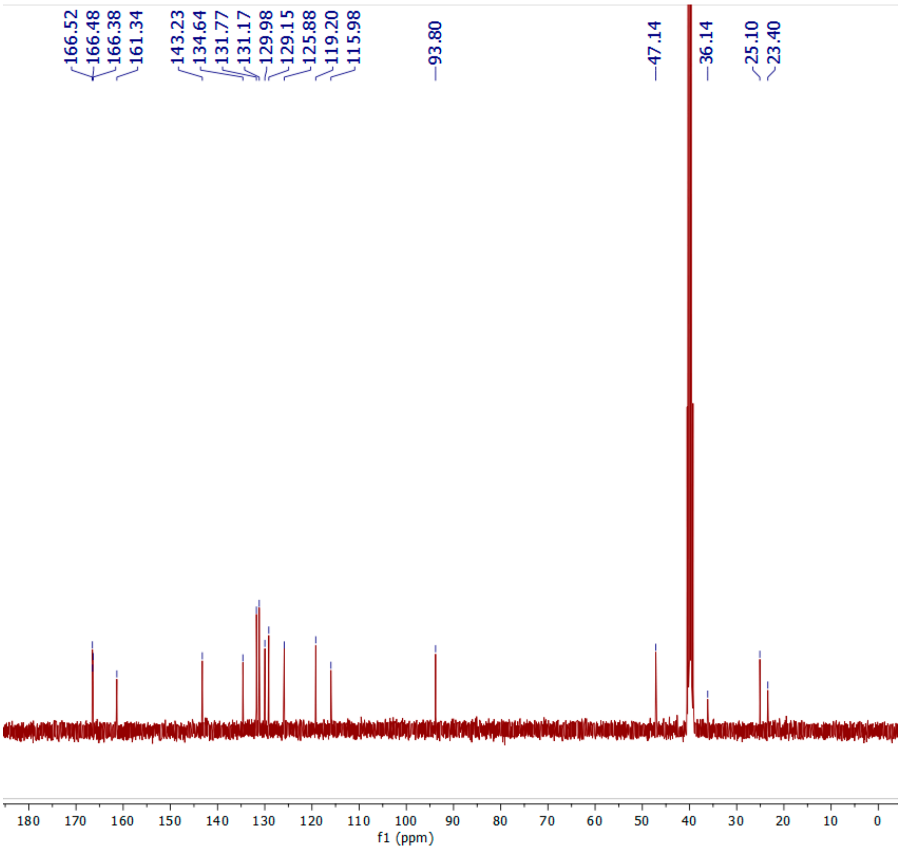

Figure S54. ^{13}C NMR spectrum of compound M7

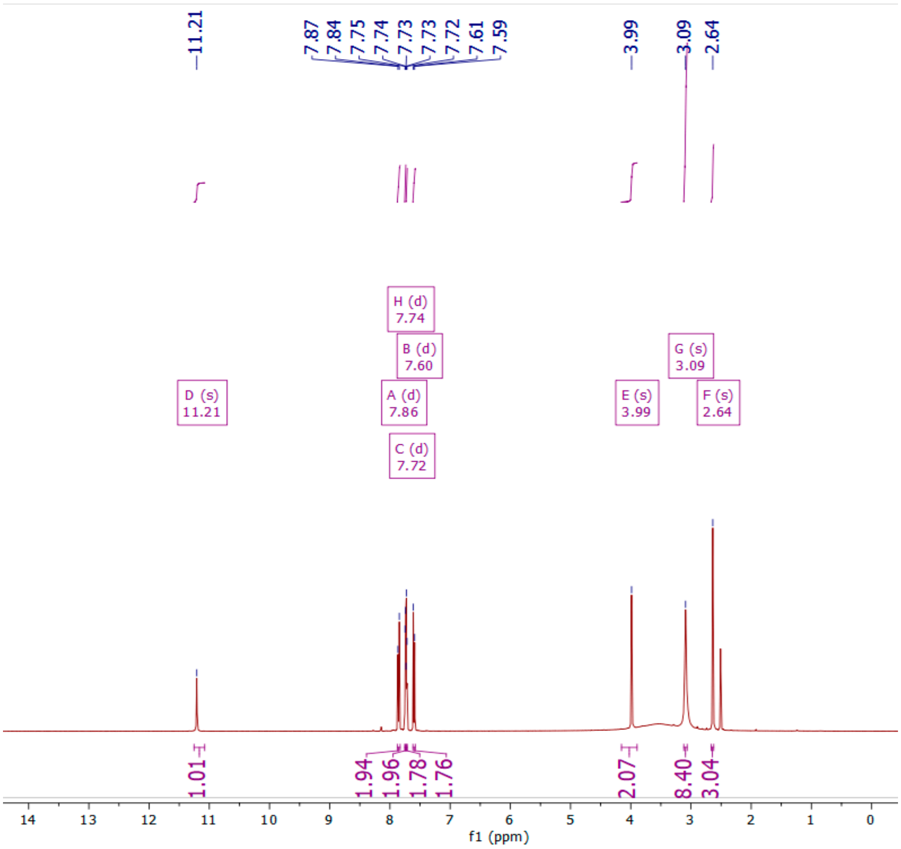

Figure S55. ^{1}H NMR spectrum of compound M8

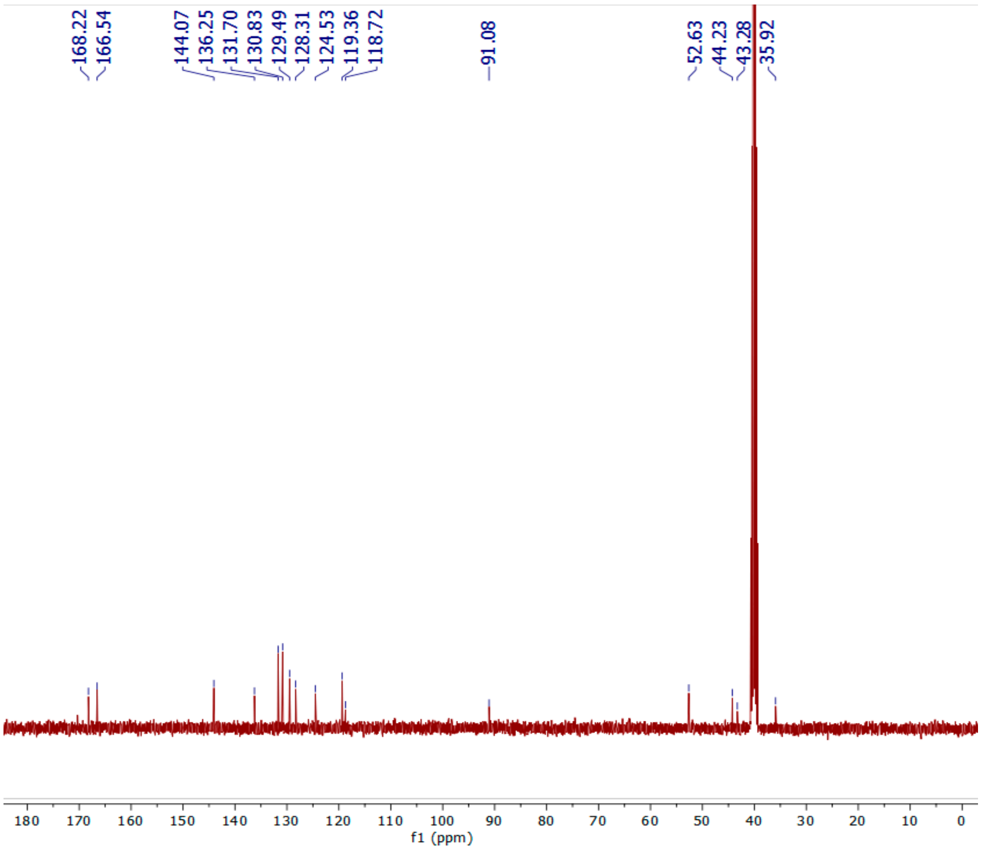

Figure S56. ^{13}C NMR spectrum of compound M8

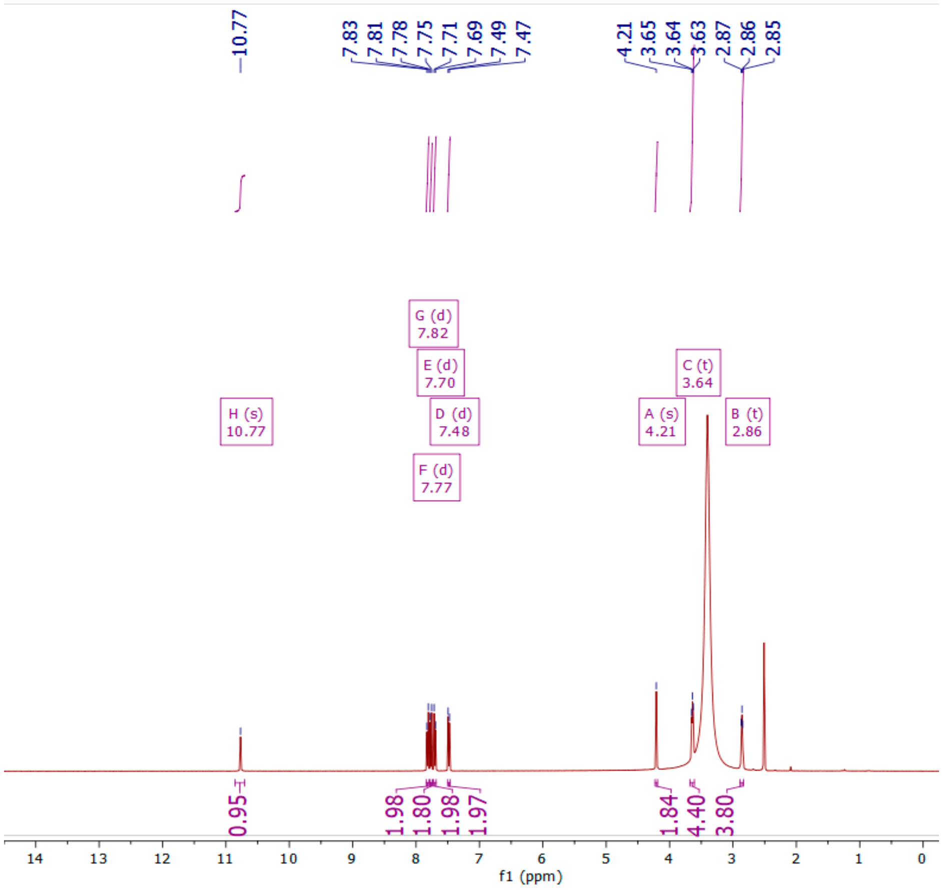

Figure S57. ^{1}H NMR spectrum of compound M9

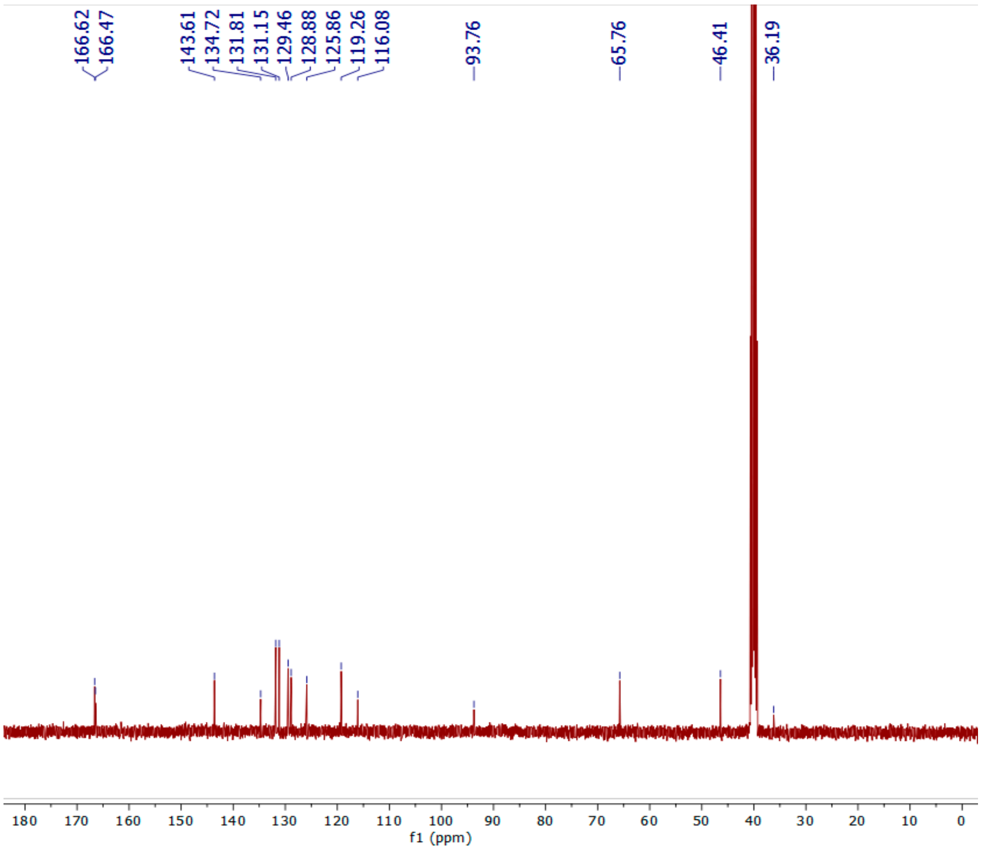

Figure S58. ^{13}C NMR spectrum of compound M9

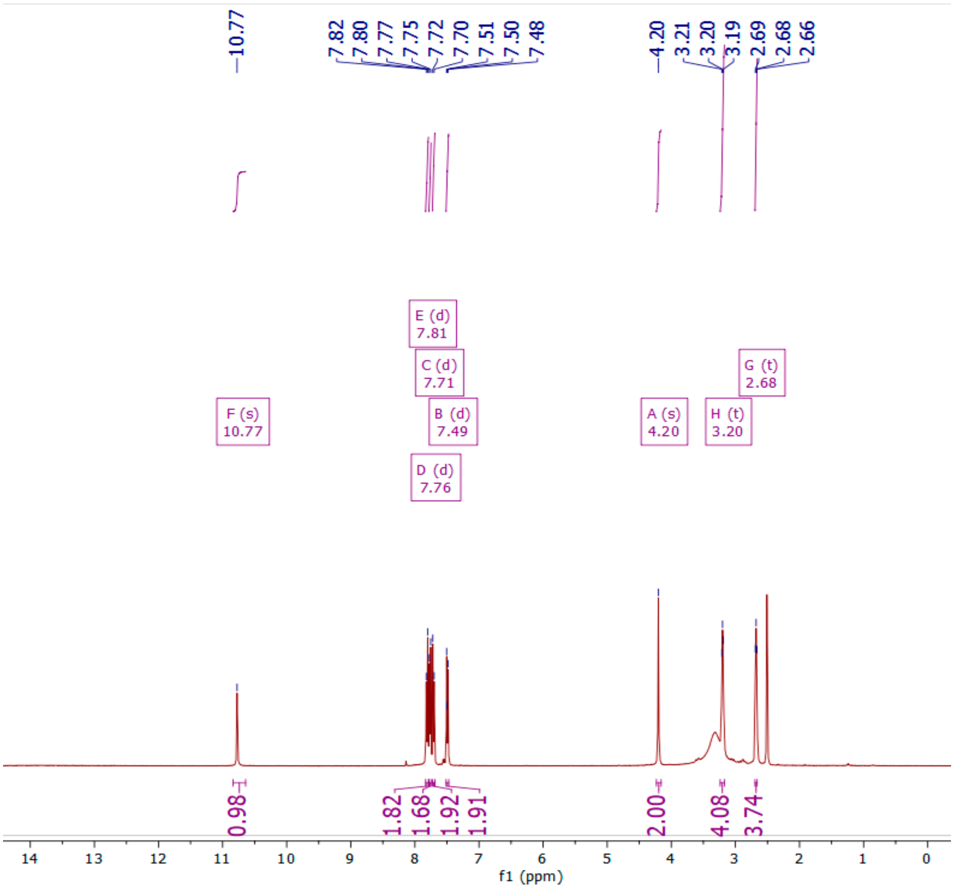

Figure S59. ^{1}H NMR spectrum of compound M10

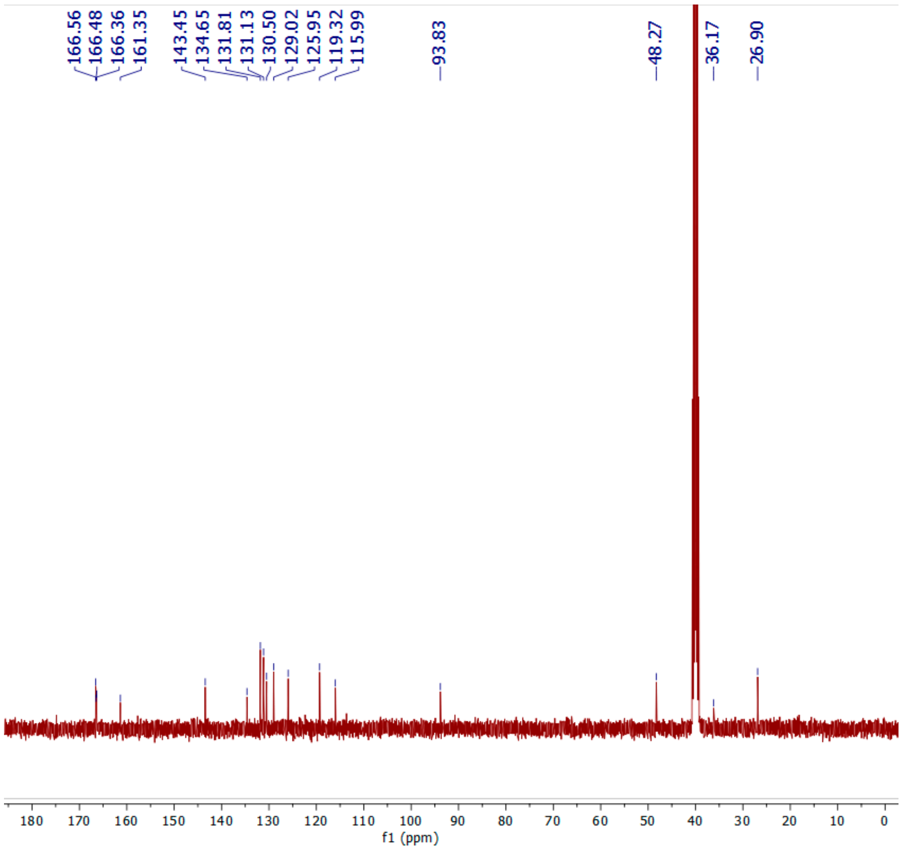

Figure S60. ^{13}C NMR spectrum of compound M10

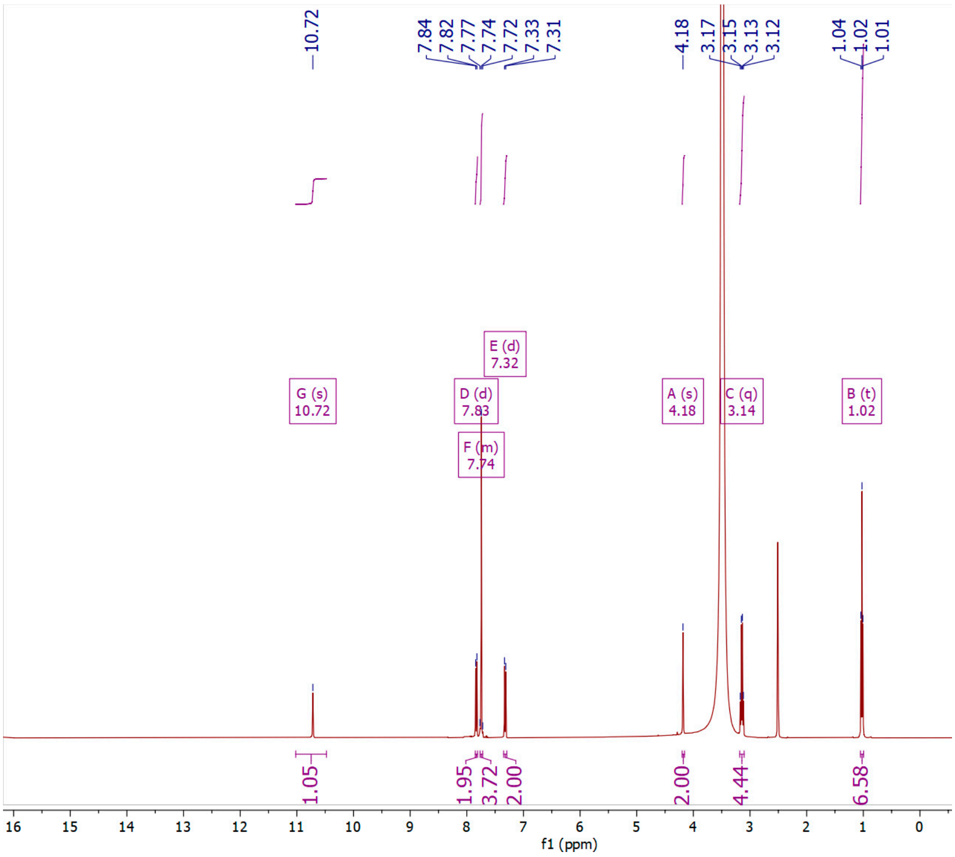

Figure S61. ¹H NMR spectrum of compound M11

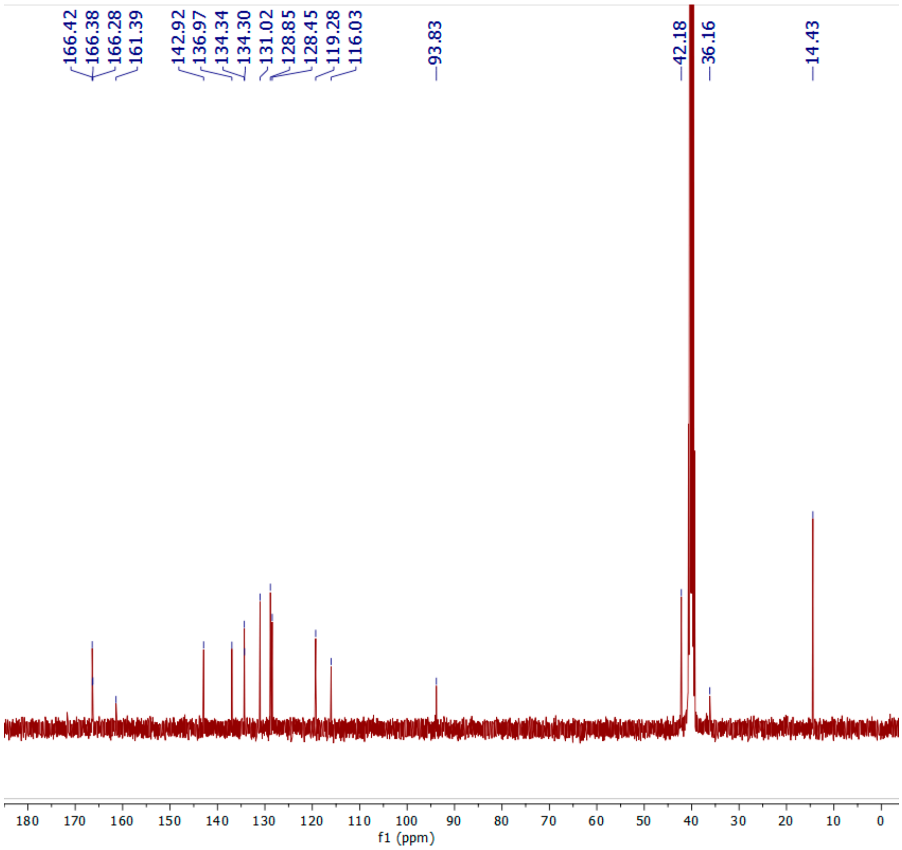

Figure S62. ^{13}C NMR spectrum of compound M11

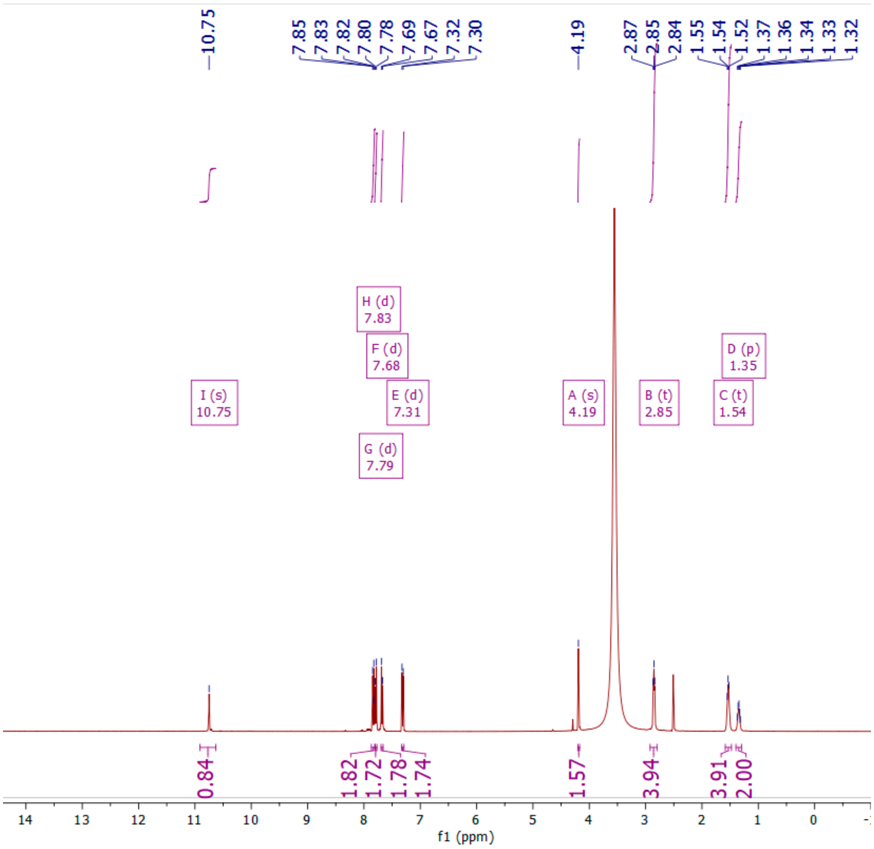

Figure S63. ^{1}H NMR spectrum of compound M12

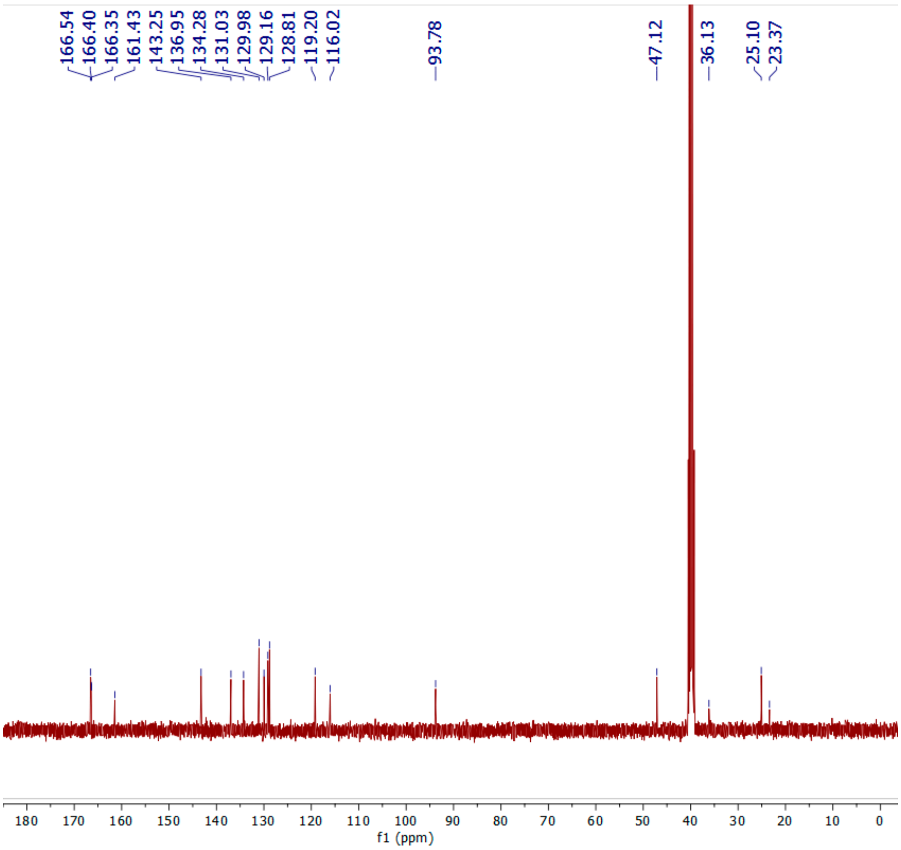

Figure S64. ^{13}C NMR spectrum of compound M12

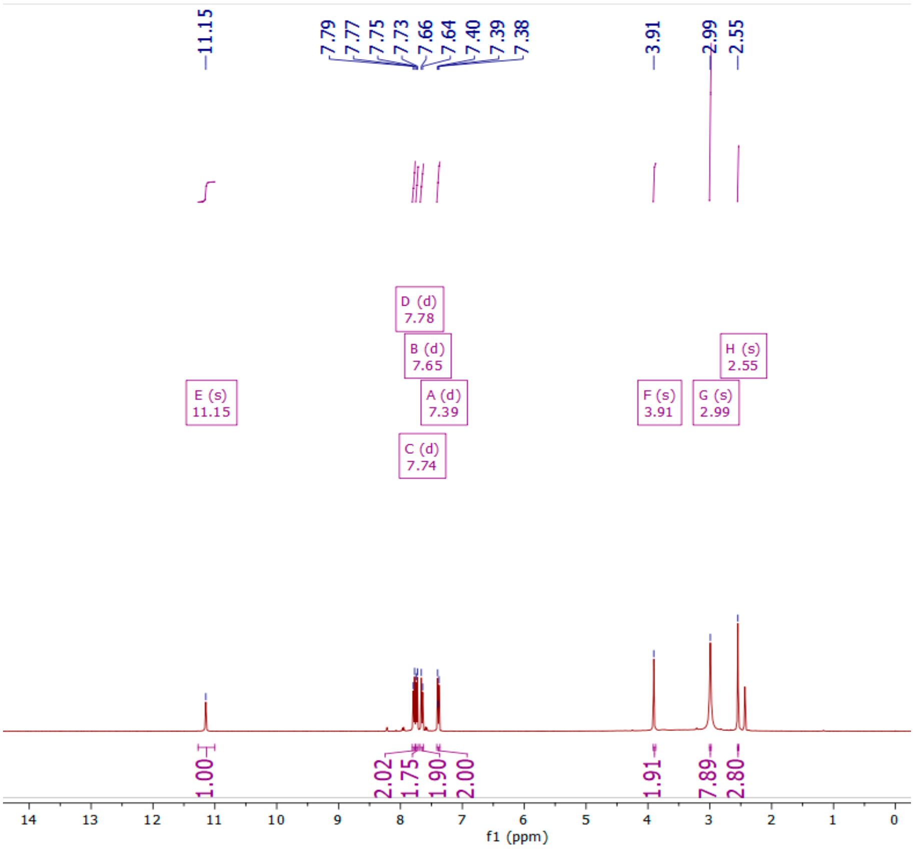

Figure S65. ^{1}H NMR spectrum of compound M13

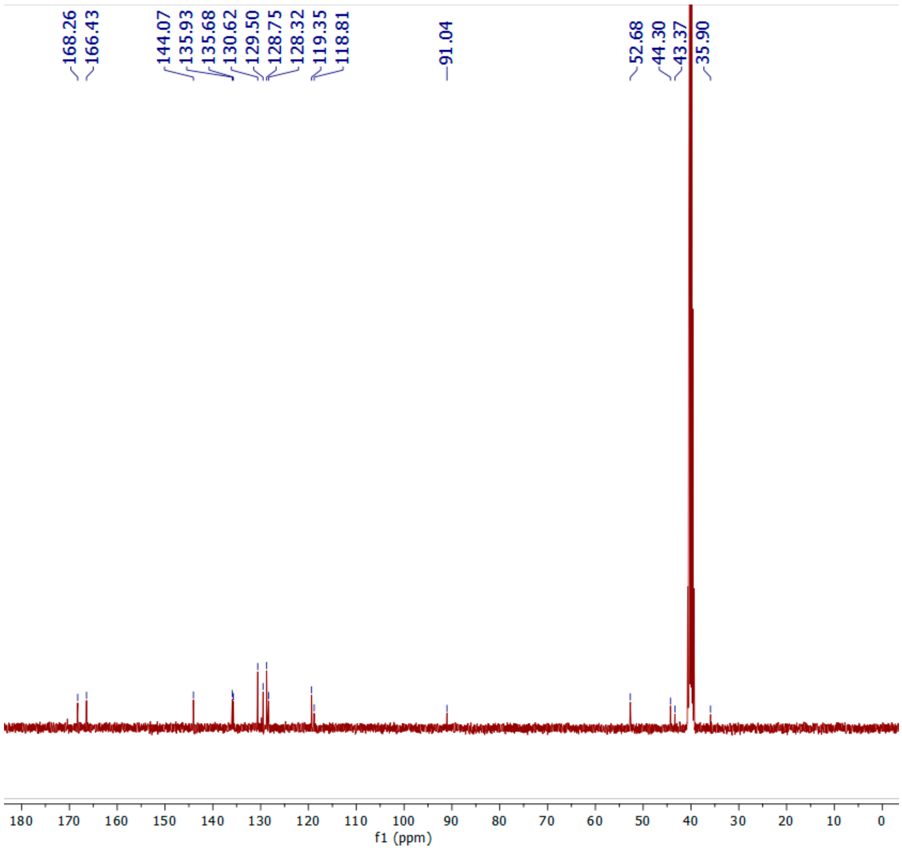

Figure S66. ^{13}C NMR spectrum of compound M13

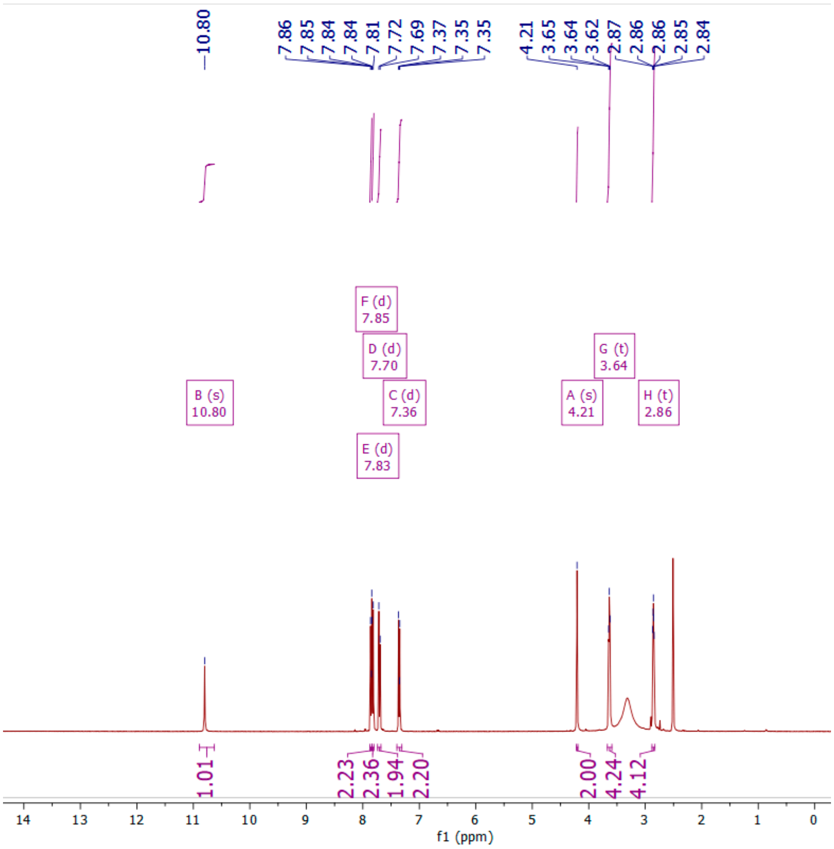

Figure S67. ^{1}H NMR spectrum of compound M14

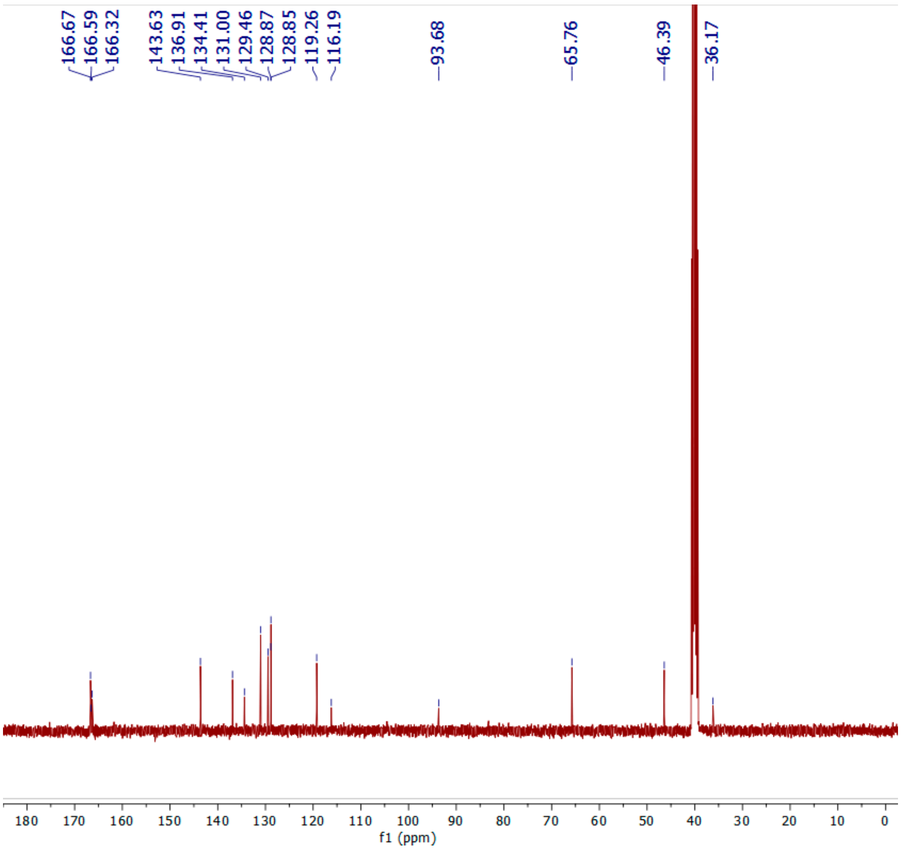

Figure S68. ^{13}C NMR spectrum of compound M14

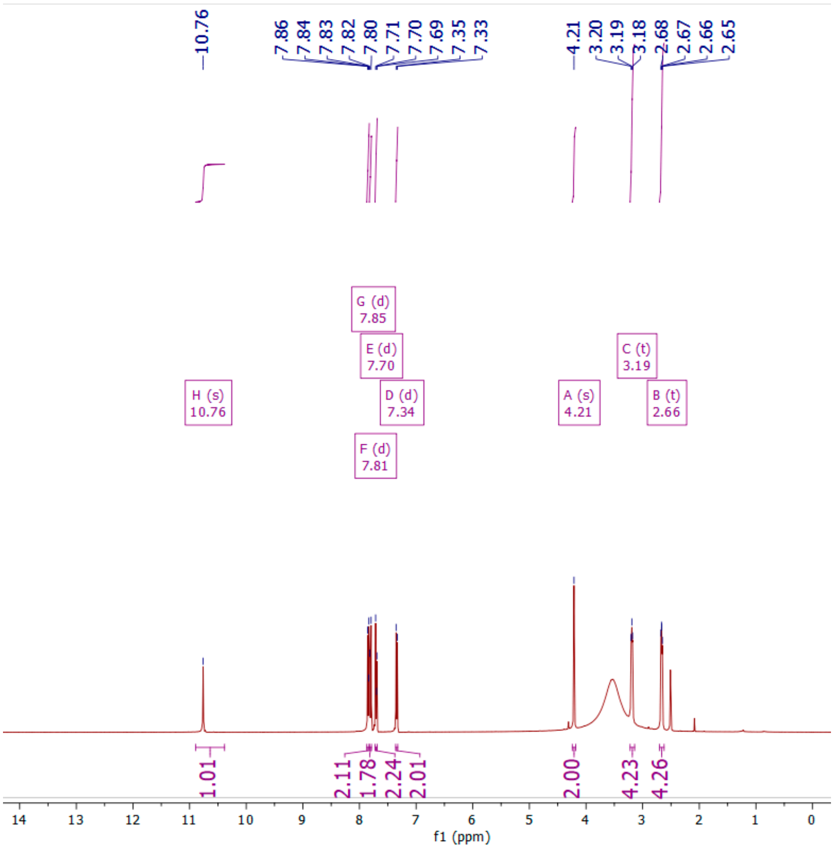

Figure S69. ^{1}H NMR spectrum of compound M15

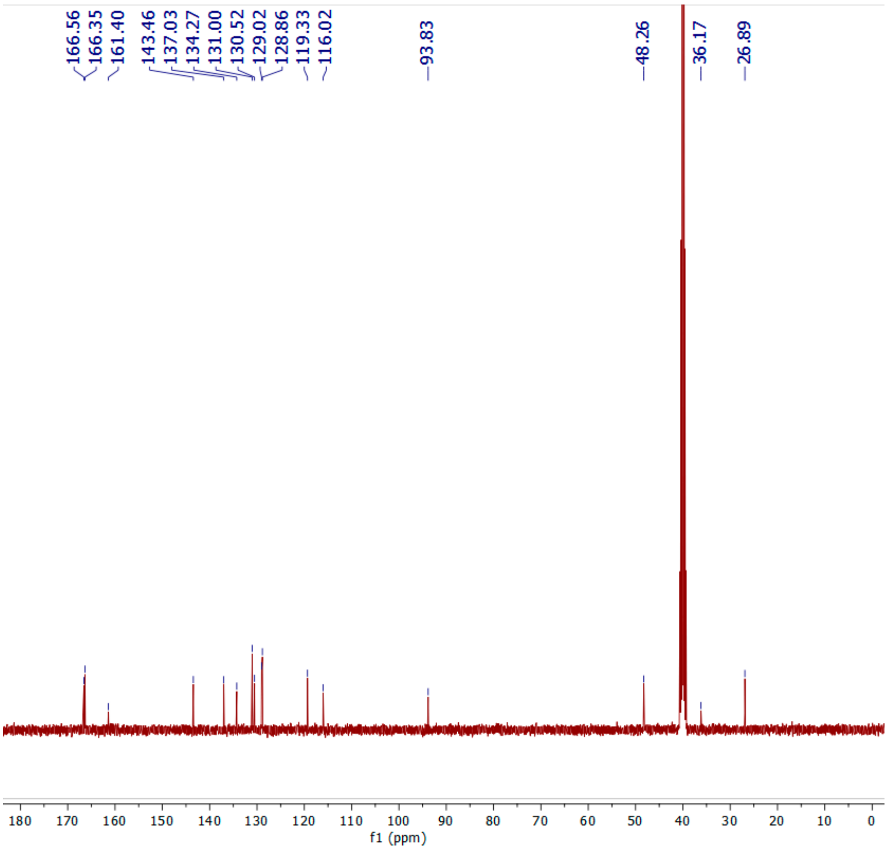

Figure S70. ^{13}C NMR spectrum of compound M15

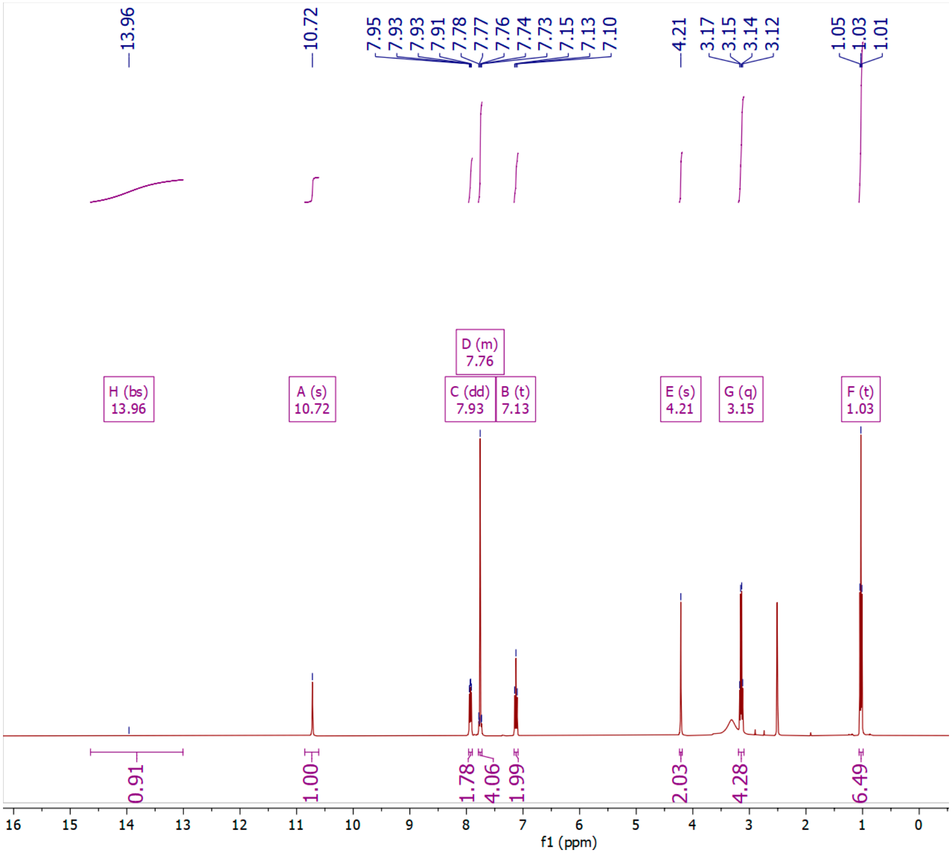

Figure S71. ^{1}H NMR spectrum of compound M16

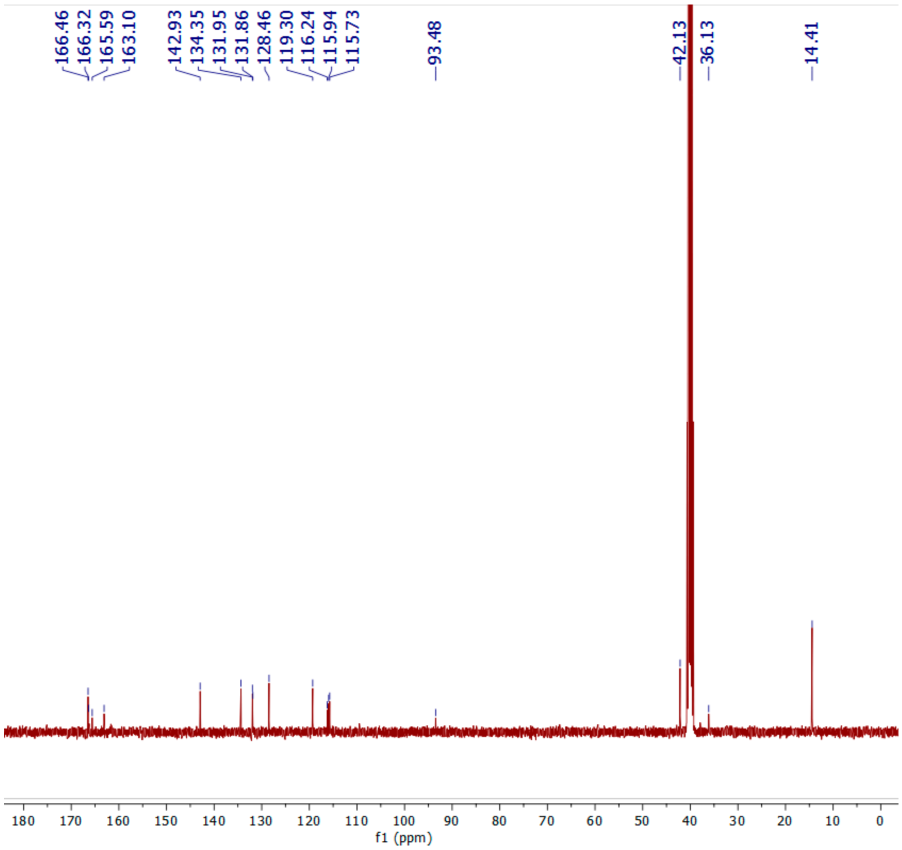

Figure S72. ^{13}C NMR spectrum of compound M16

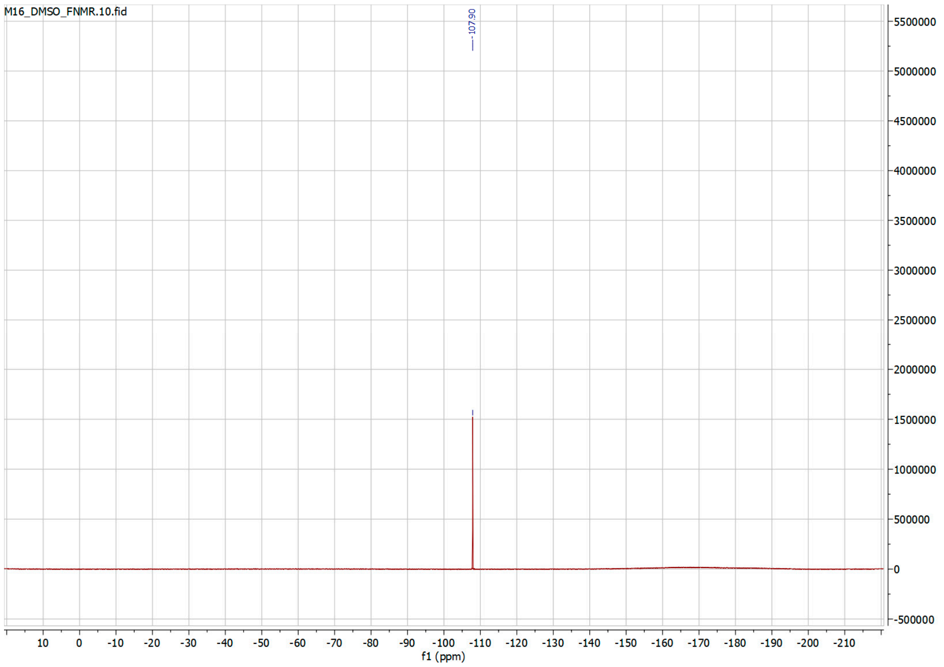

Figure S73. ^{19}FNMR spectrum of compound M16

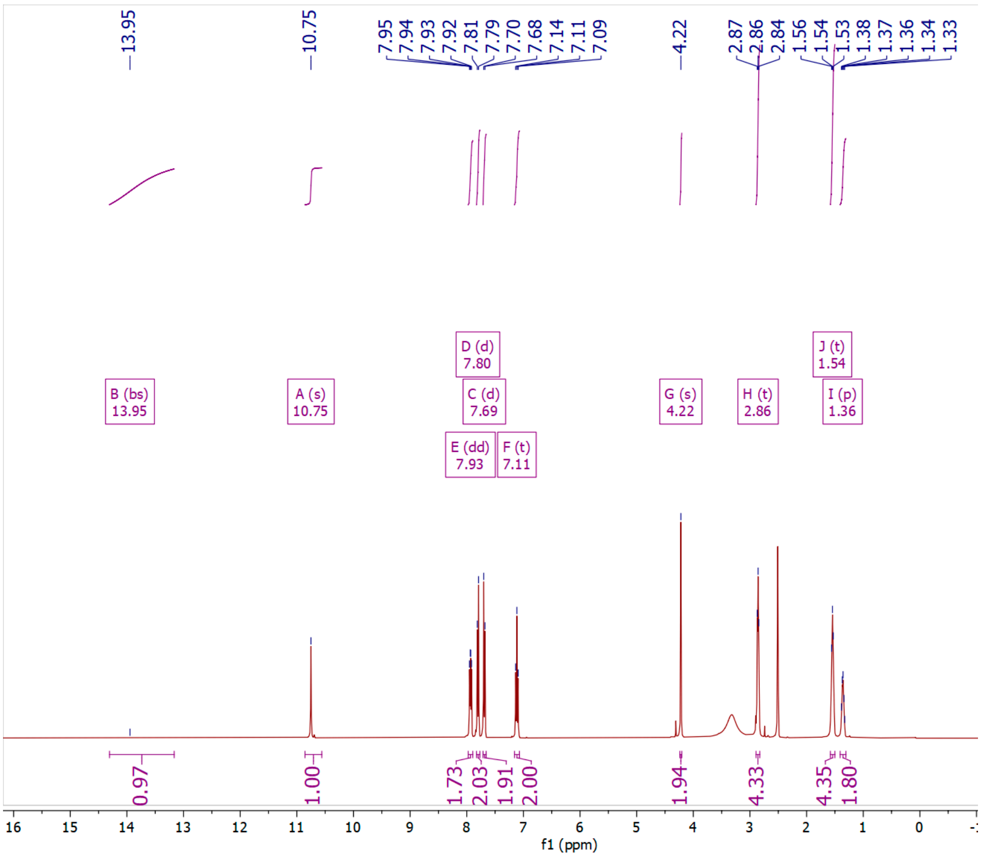

Figure S74. ^{1}HNMR spectrum of compound M17

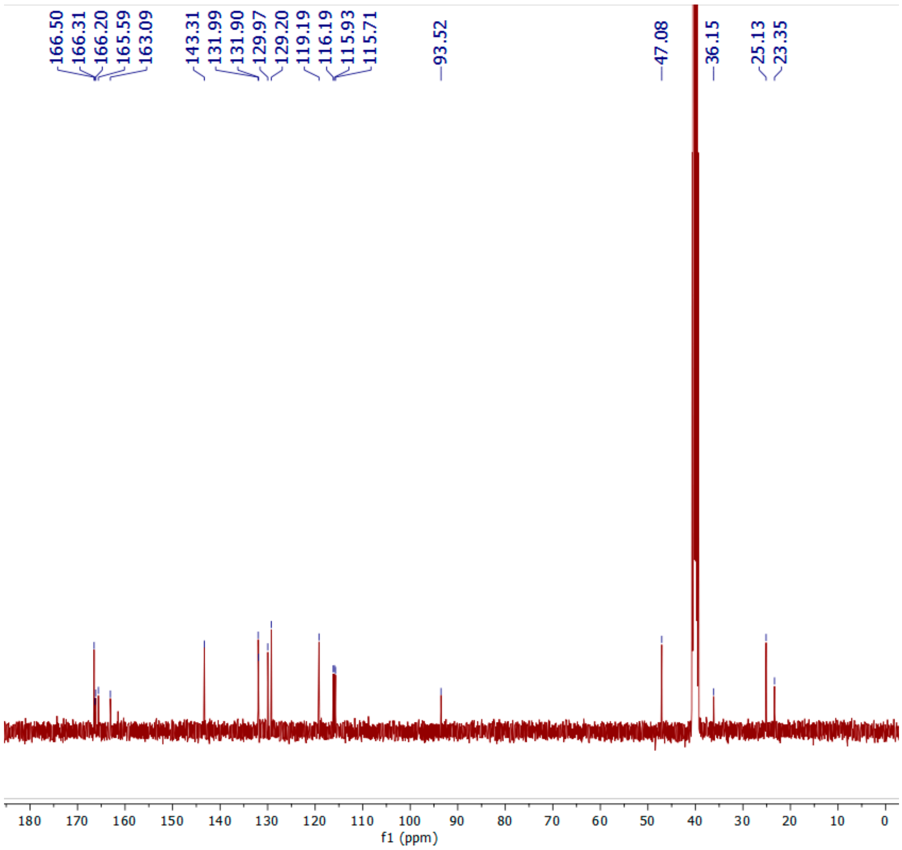

Figure S75. ^{13}CNMR spectrum of compound M17

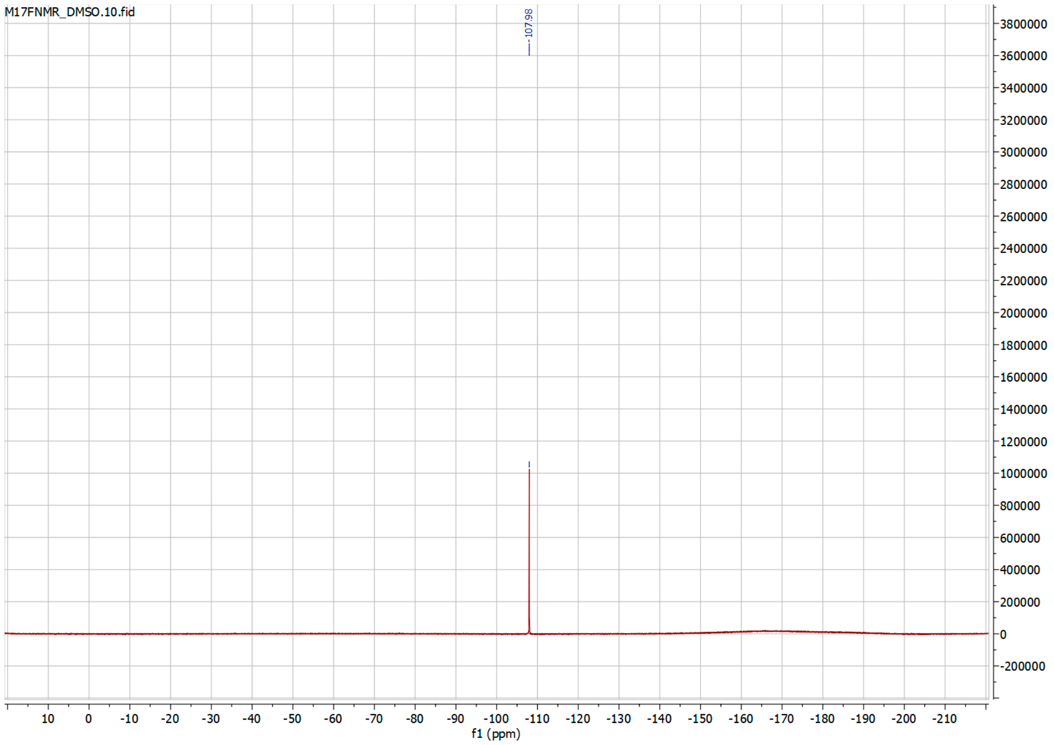

Figure S76. ^{19}FNMR spectrum of compound M17

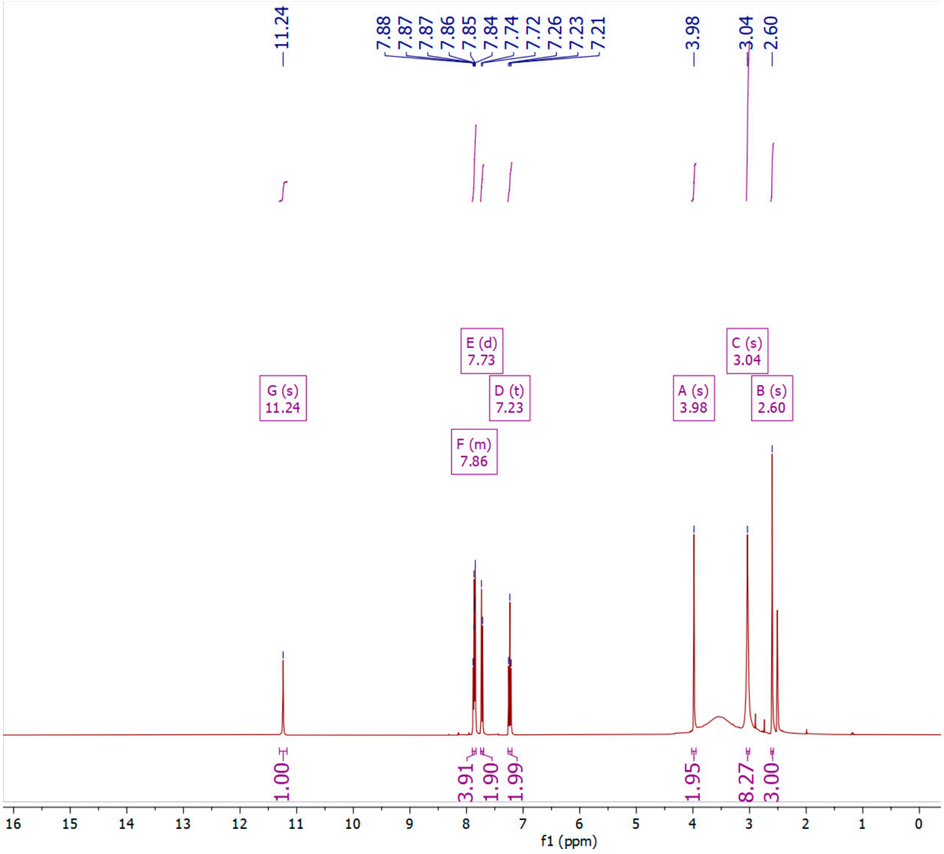

Figure S77. ¹H NMR spectrum of compound M18

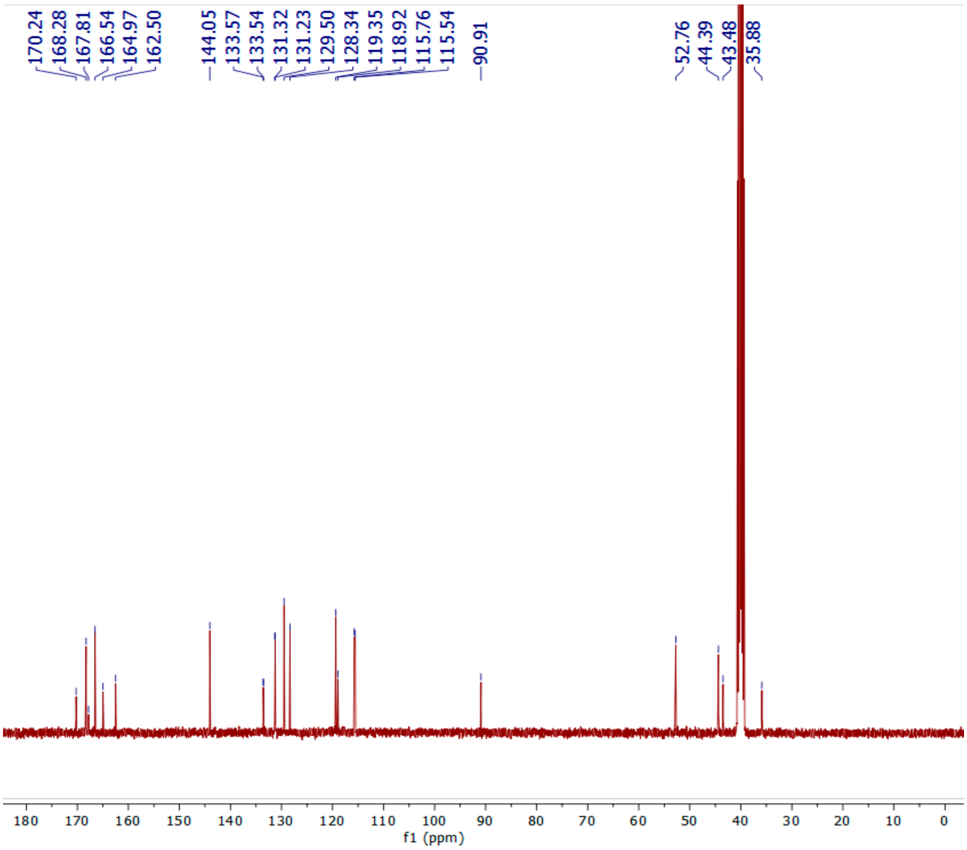

Figure S78. ^{13}CNMR spectrum of compound M18

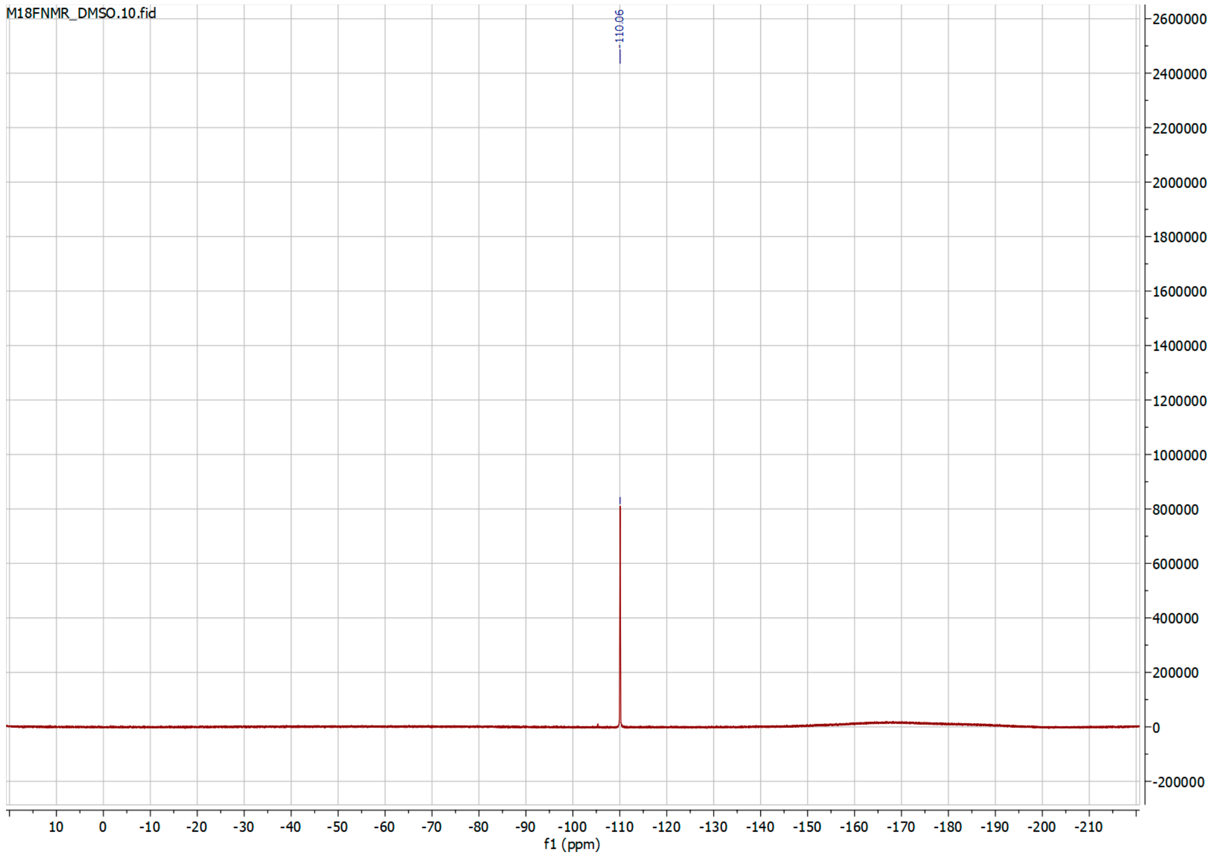

Figure S79. ^{19}FNMR spectrum of compound M18

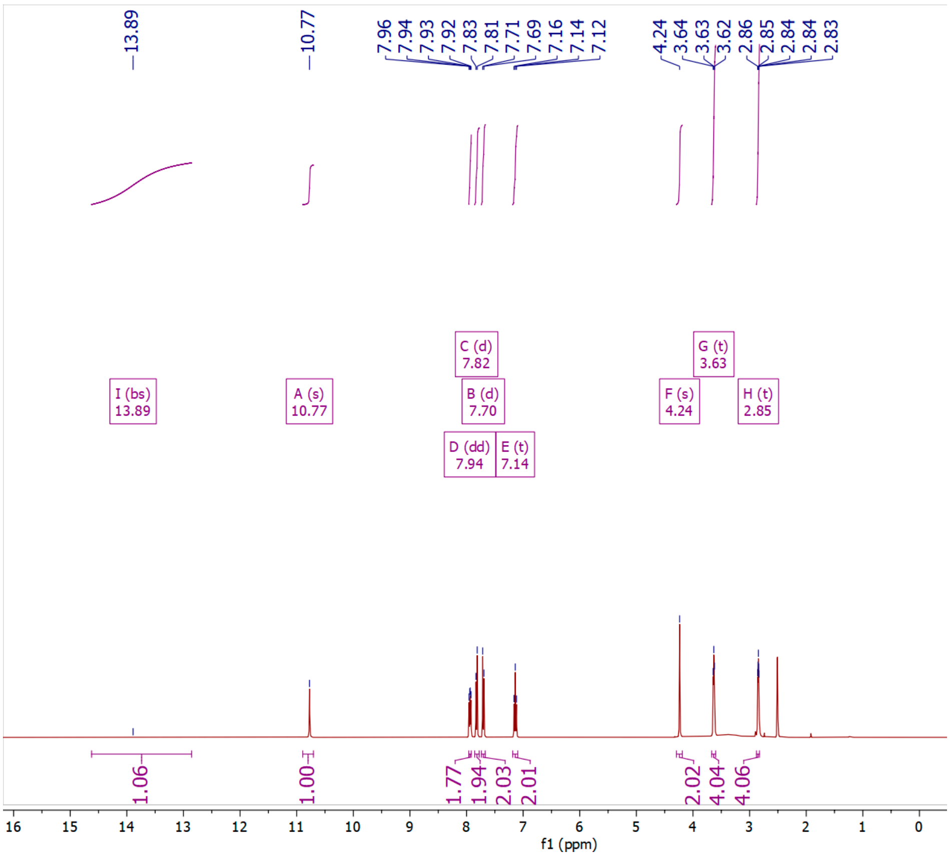

Figure S80. ^{1}HNMR spectrum of compound M18

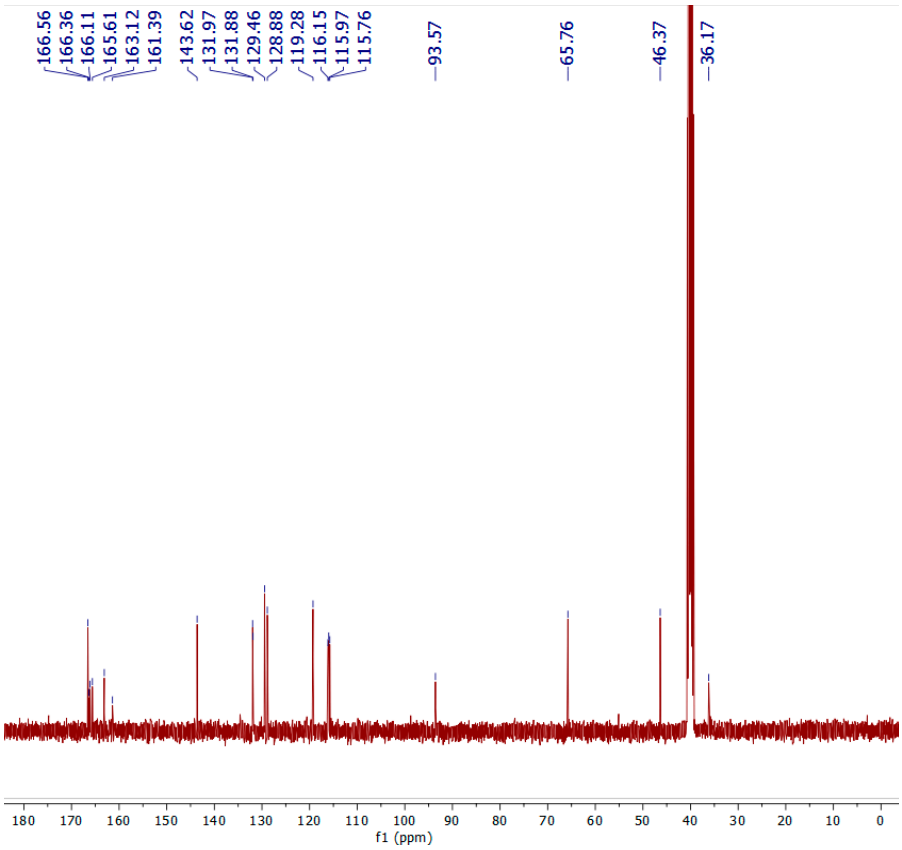

Figure S81. ^{13}CNMR spectrum of compound M18

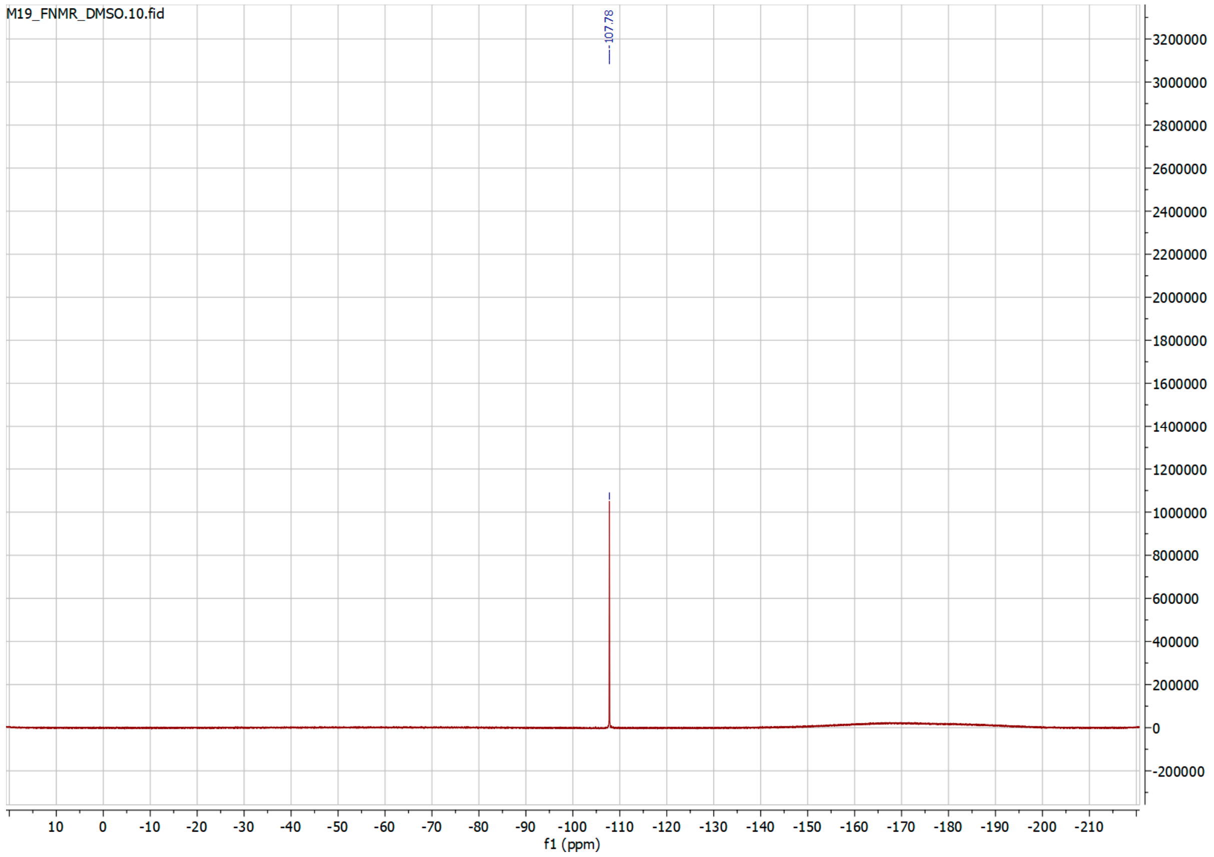

Figure S82. ^{19}FNMR spectrum of compound M18

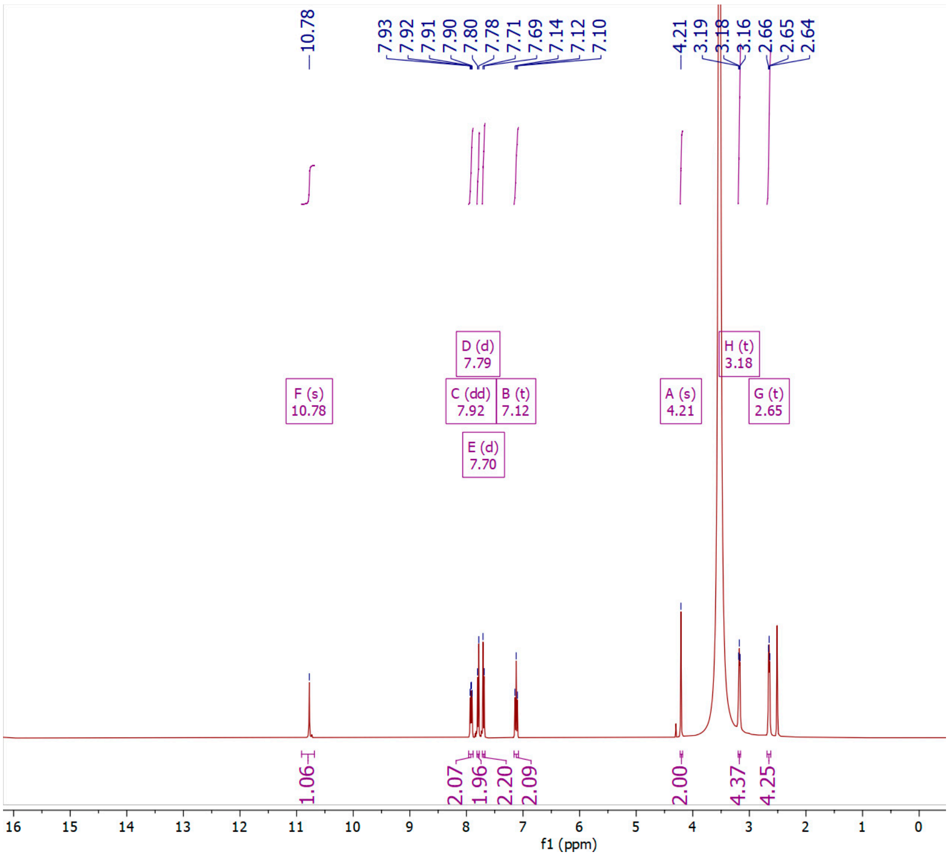

Figure S83. ^{1}HNMR spectrum of compound M20

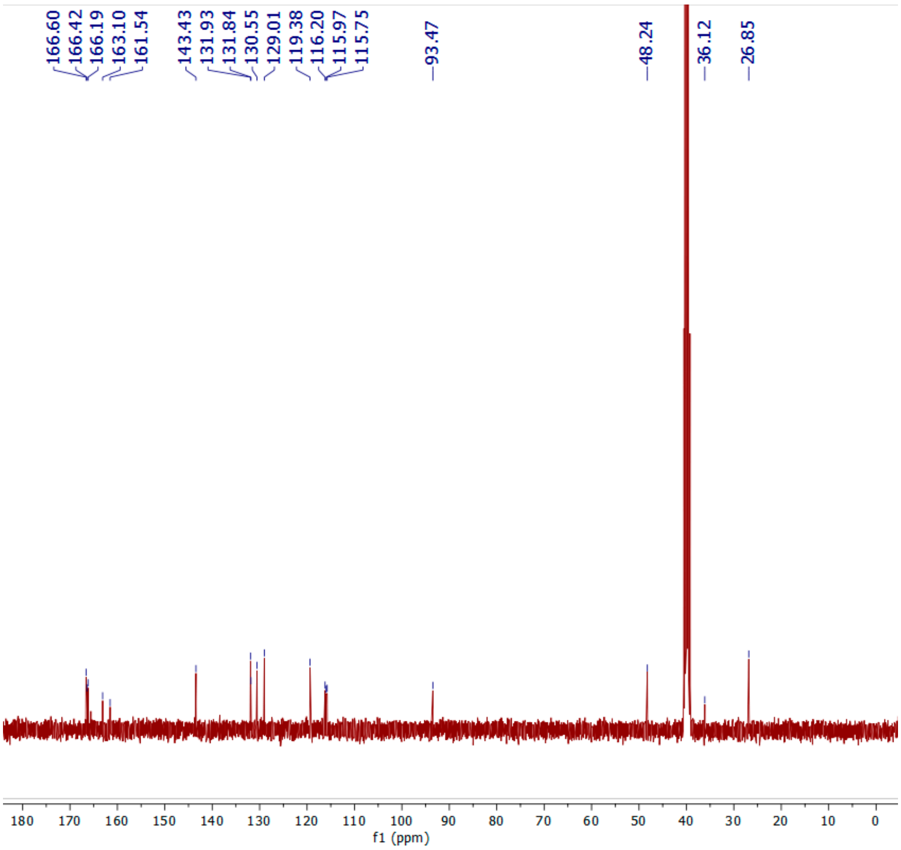

Figure S84. ^{13}CNMR spectrum of compound M20

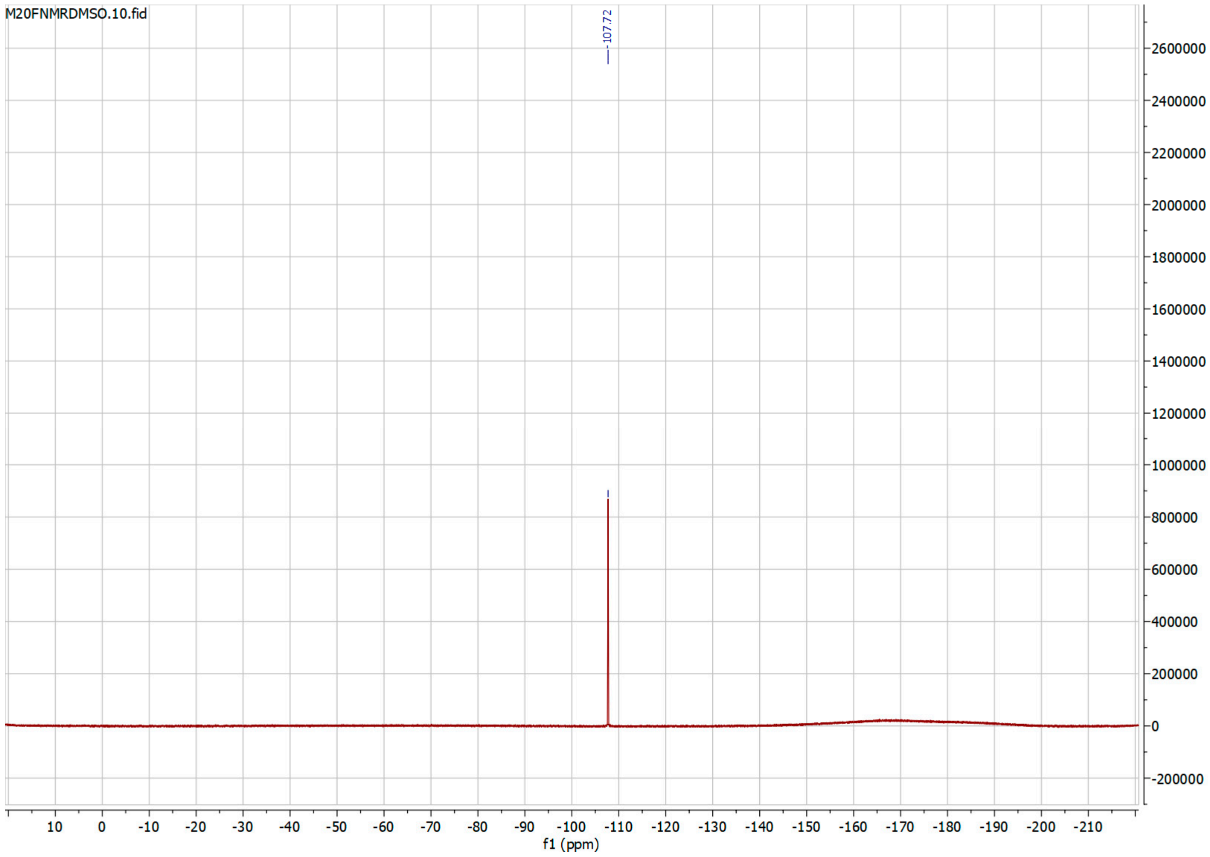

Figure S85. ^{19}FNMR spectrum of compound M20

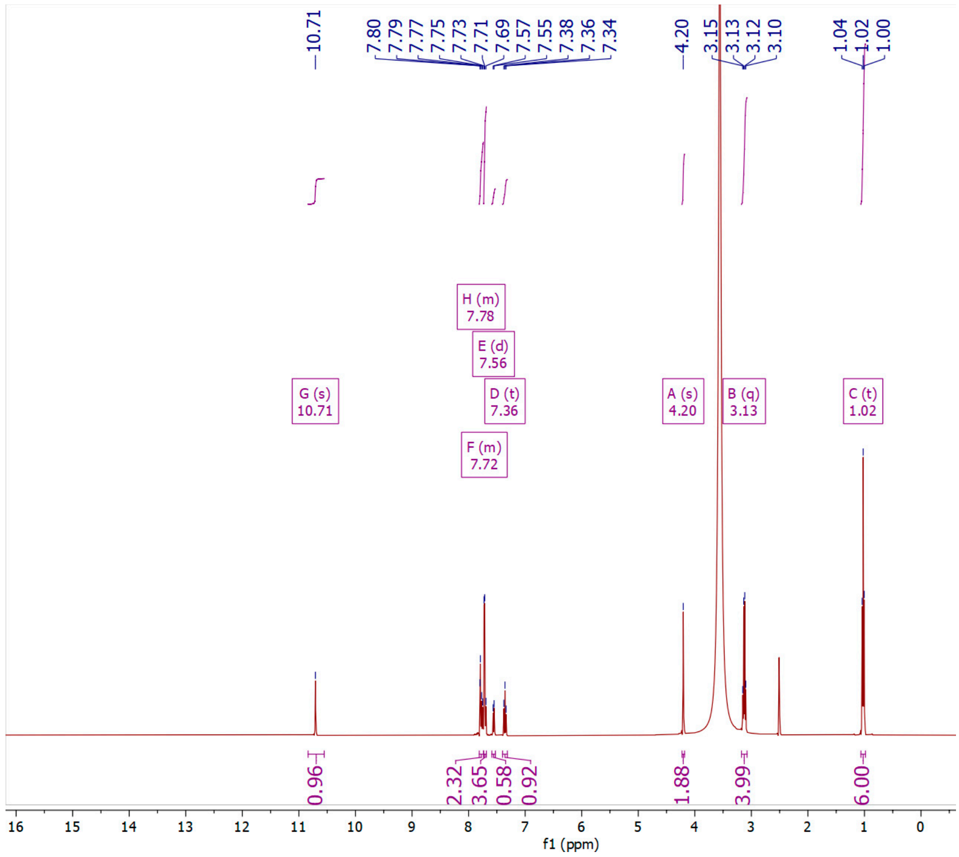

Figure S85. ^{1}HNMR spectrum of compound M21

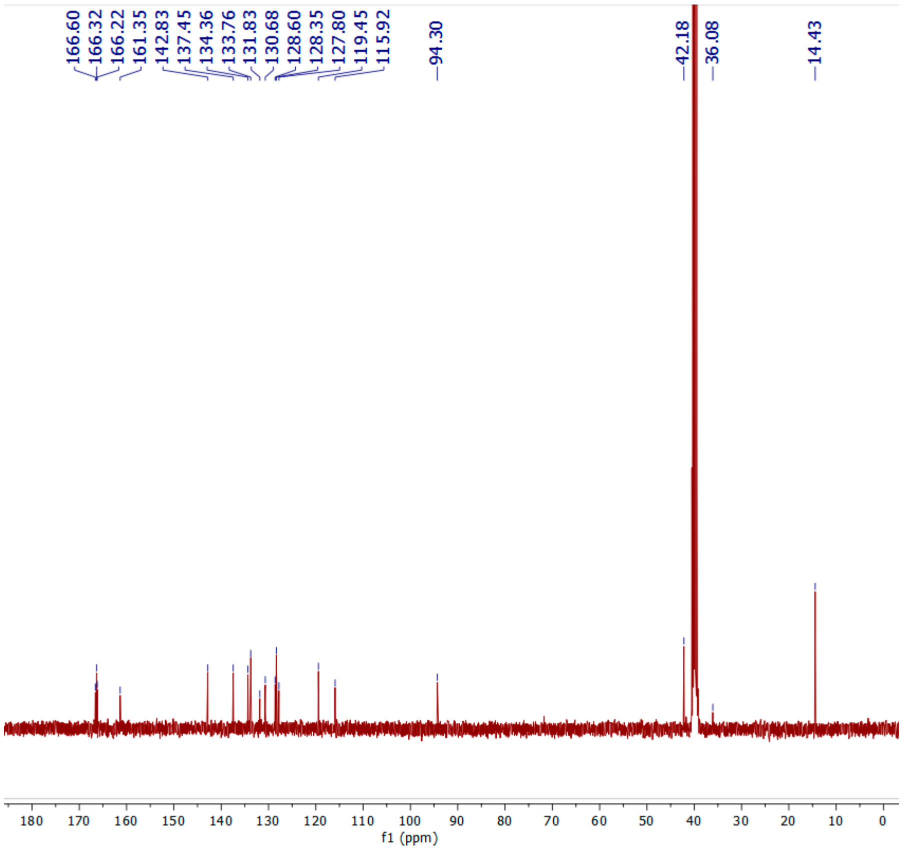

Figure S86. ^{13}CNMR spectrum of compound M21

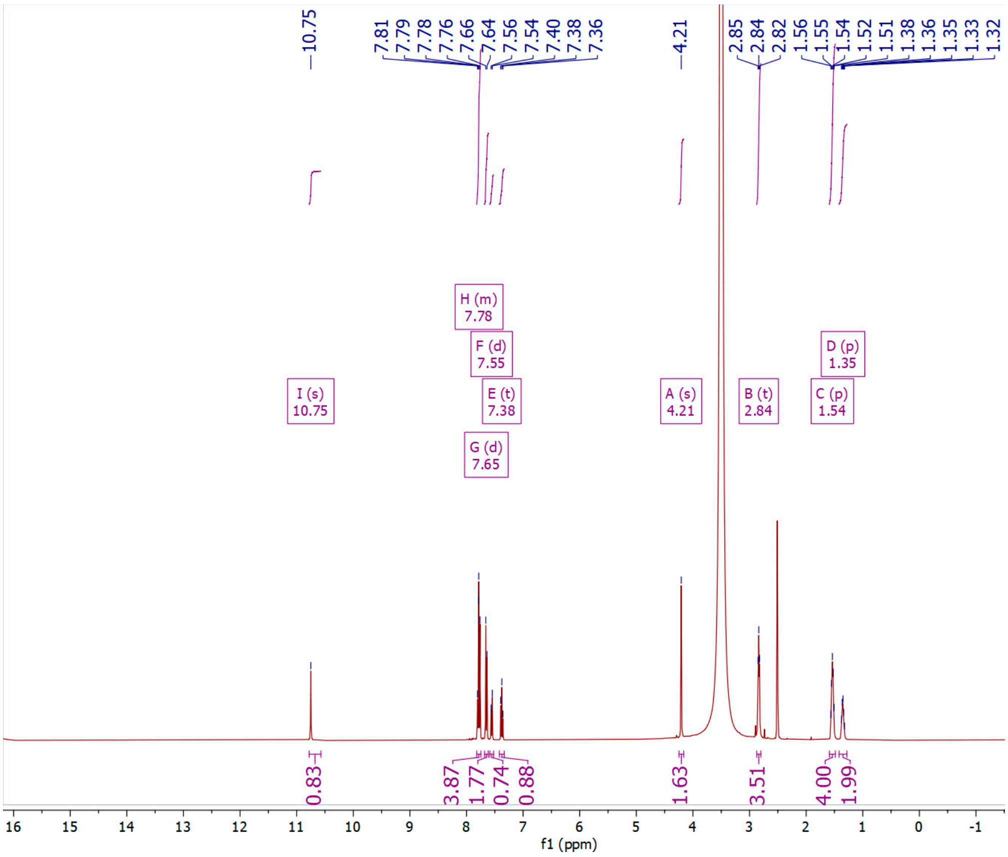

Figure S87. ¹HNMR spectrum of compound M22

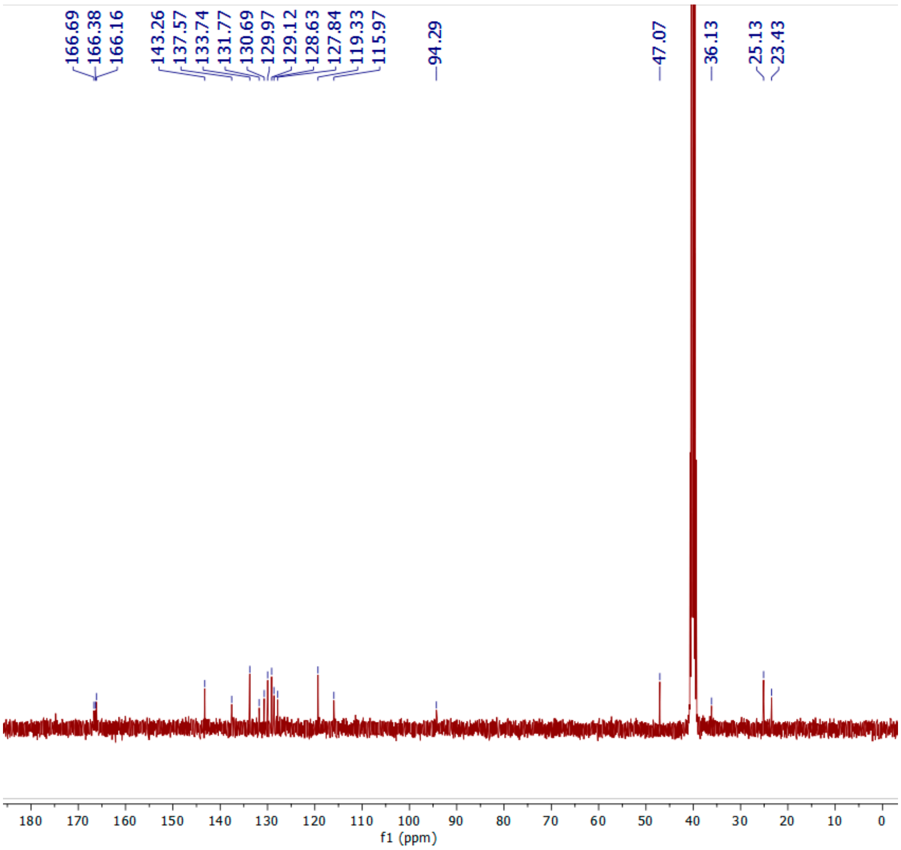

Figure S88. ^{13}CNMR spectrum of compound M22

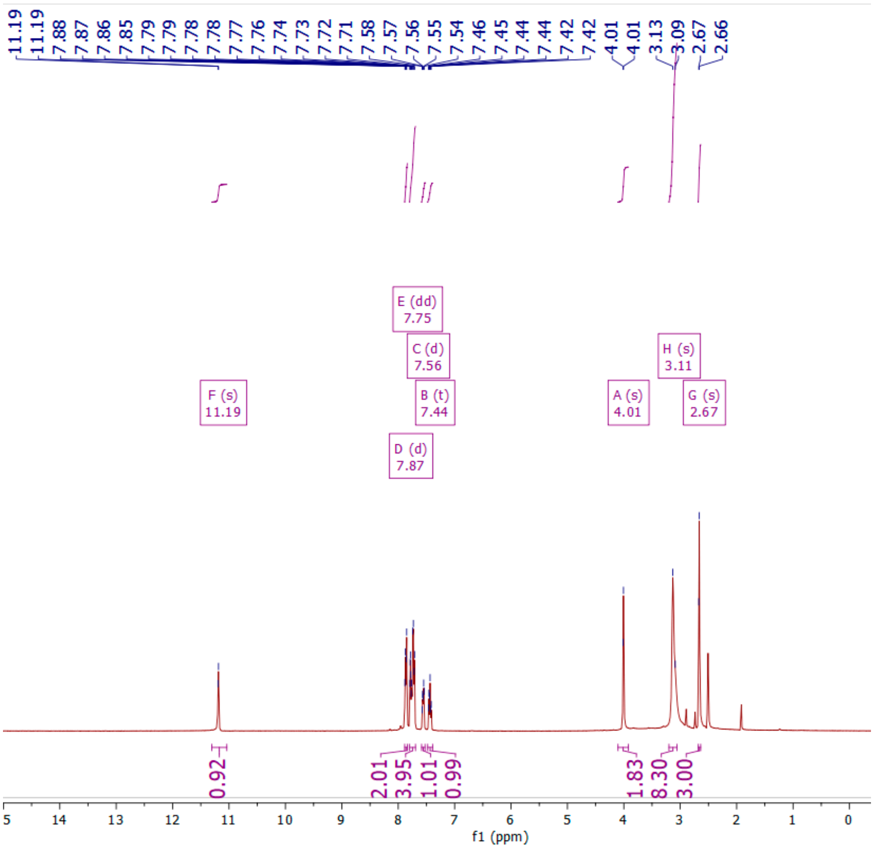

Figure S89. ¹HNMR spectrum of compound M23

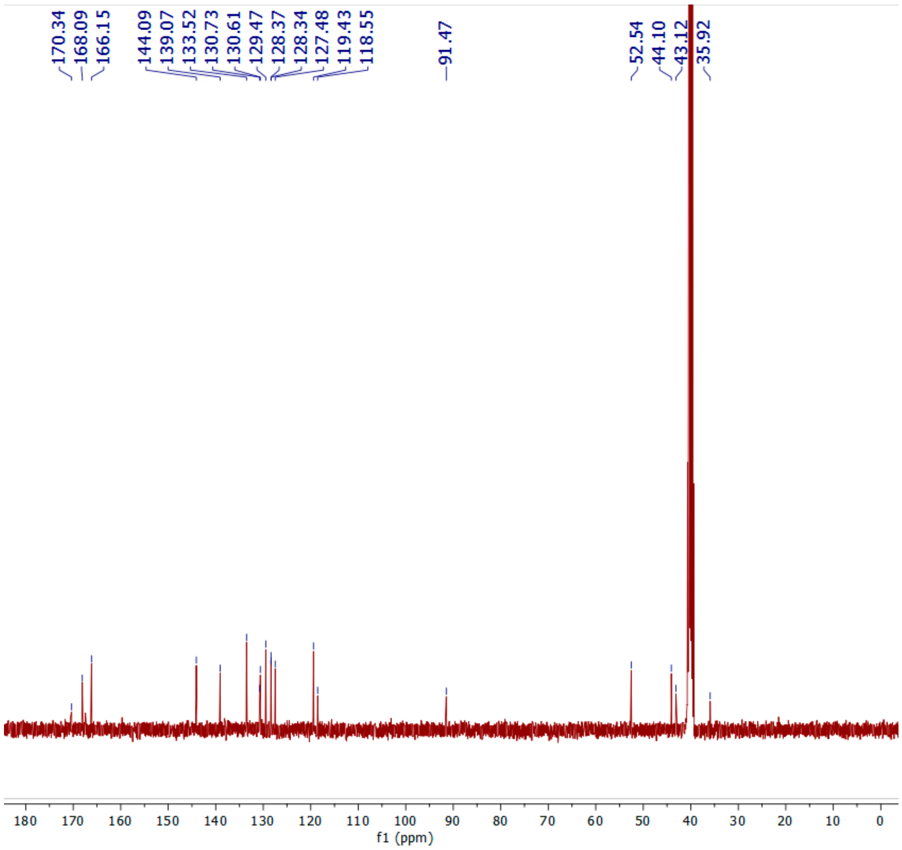

Figure S90. ^{13}CNMR spectrum of compound M23

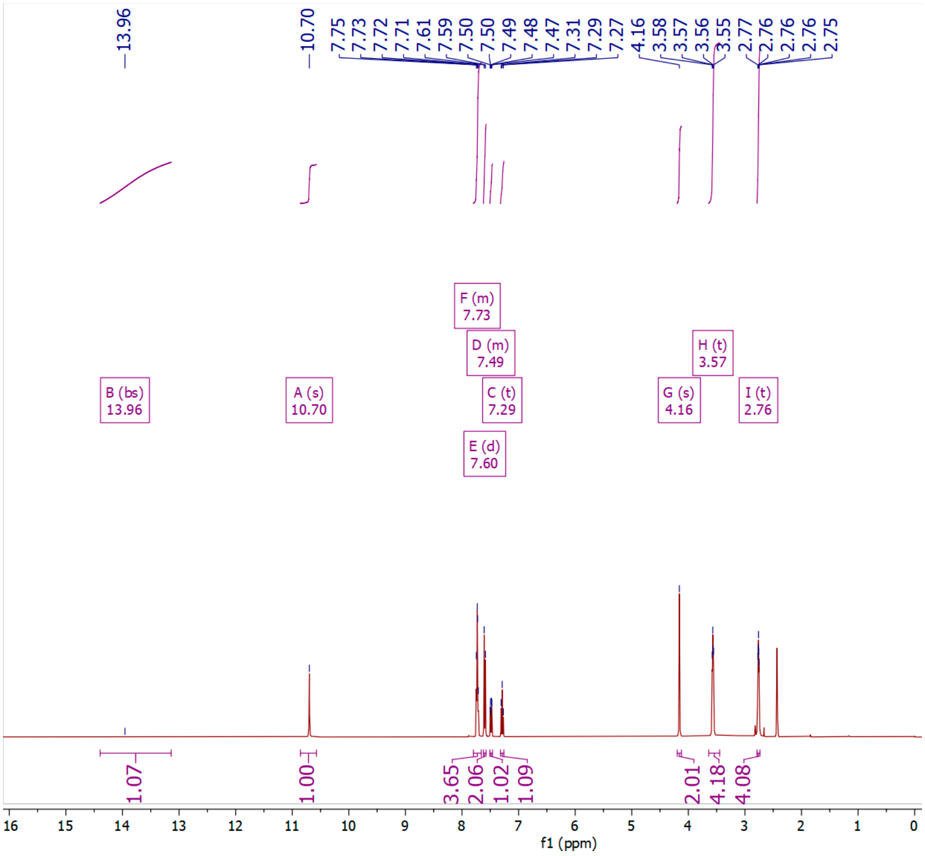

Figure S91. ^{1}HNMR spectrum of compound M24

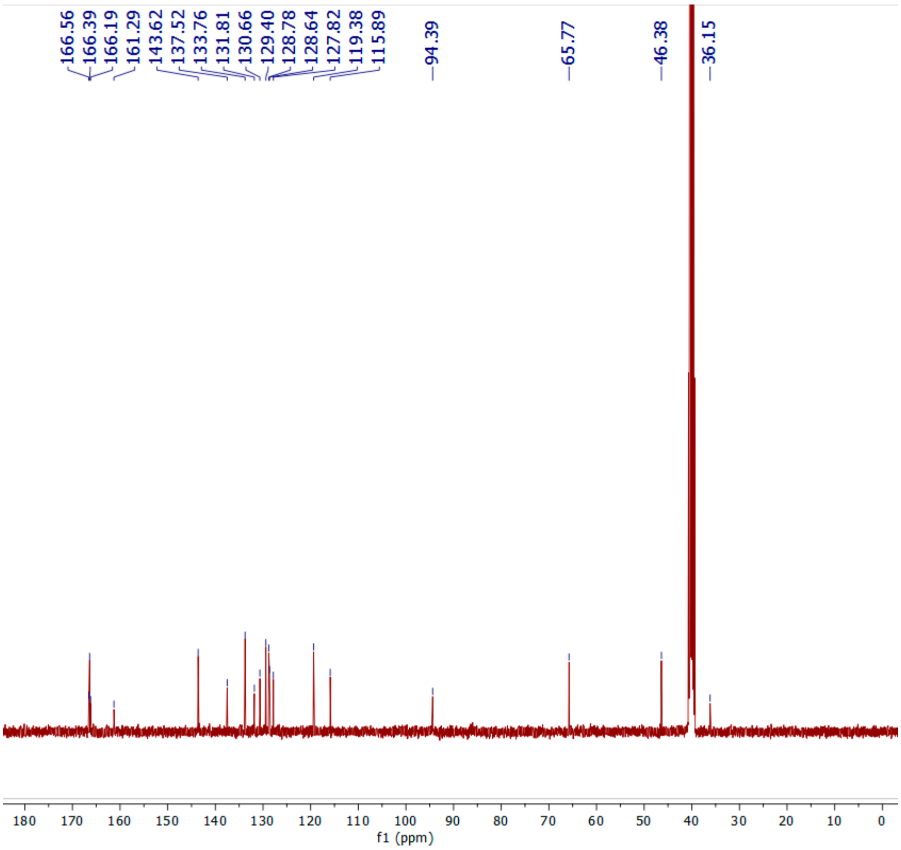

Figure S92. ^{13}CNMR spectrum of compound M24

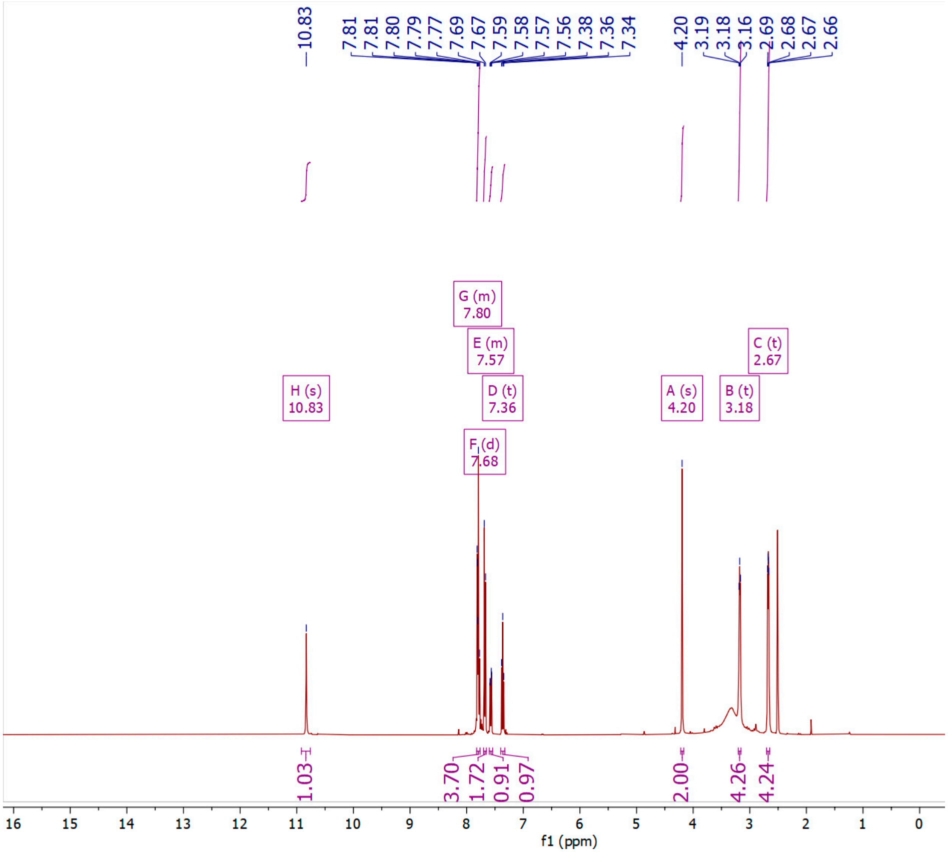

Figure S93. ^{1}HNMR spectrum of compound M25

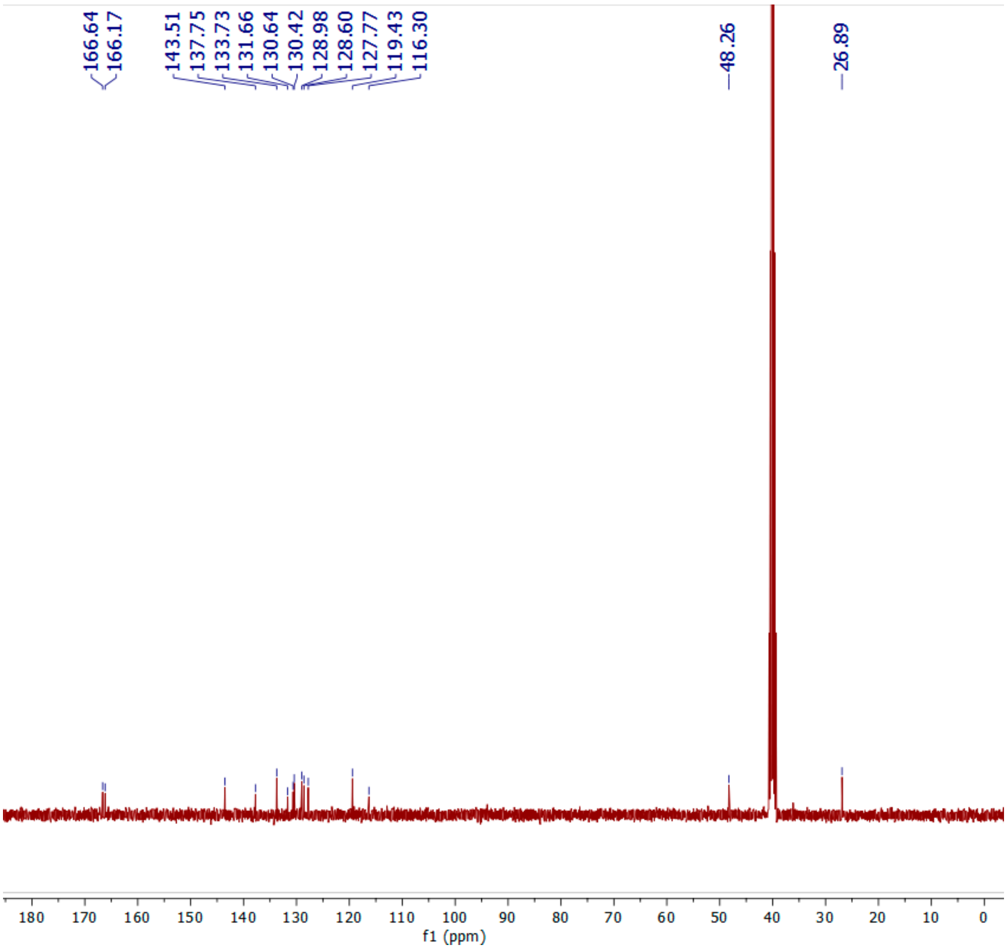

Figure S94. ^{13}CNMR spectrum of compound M25

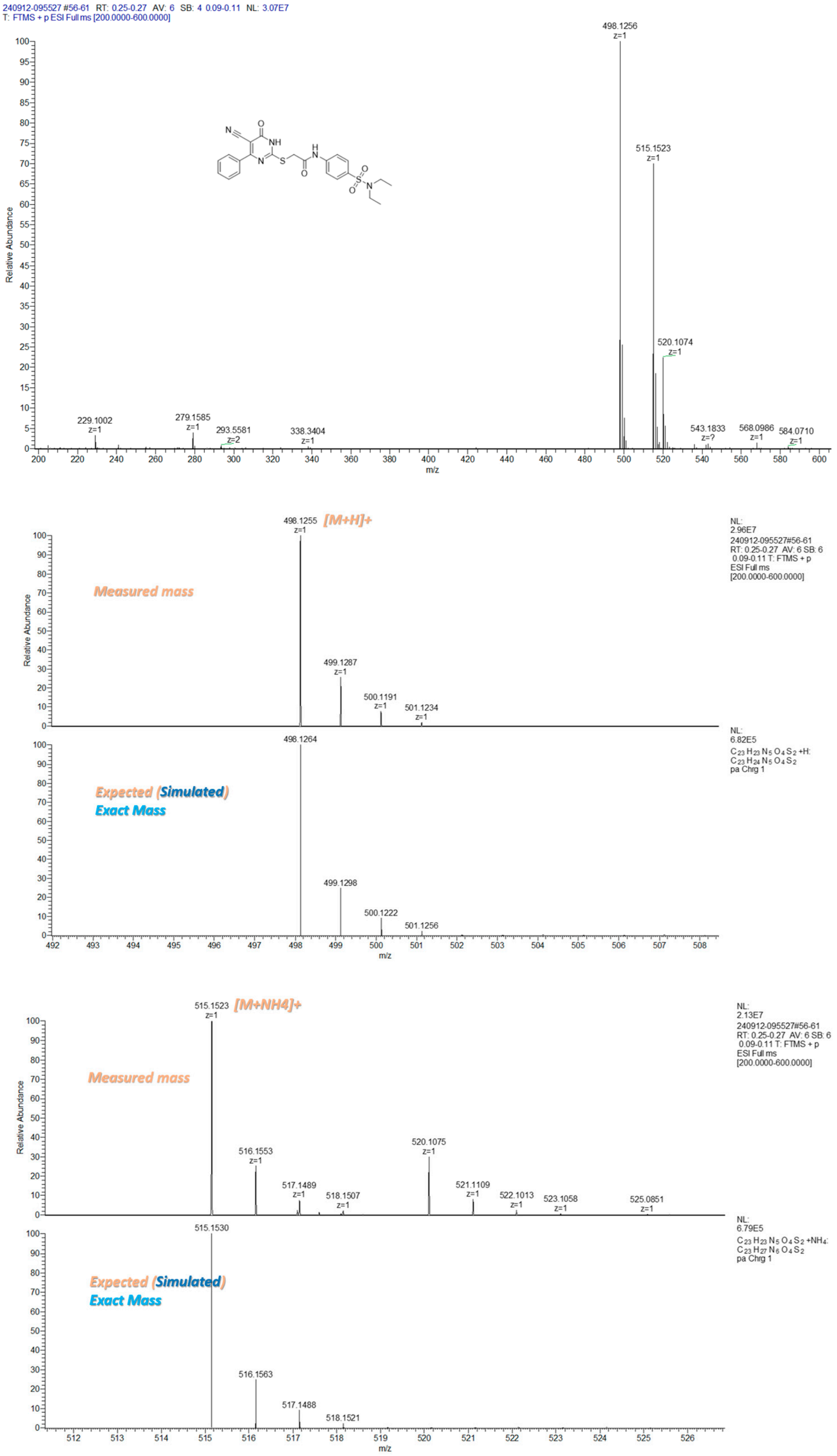

Figure S95. Mass analysis of compound M1

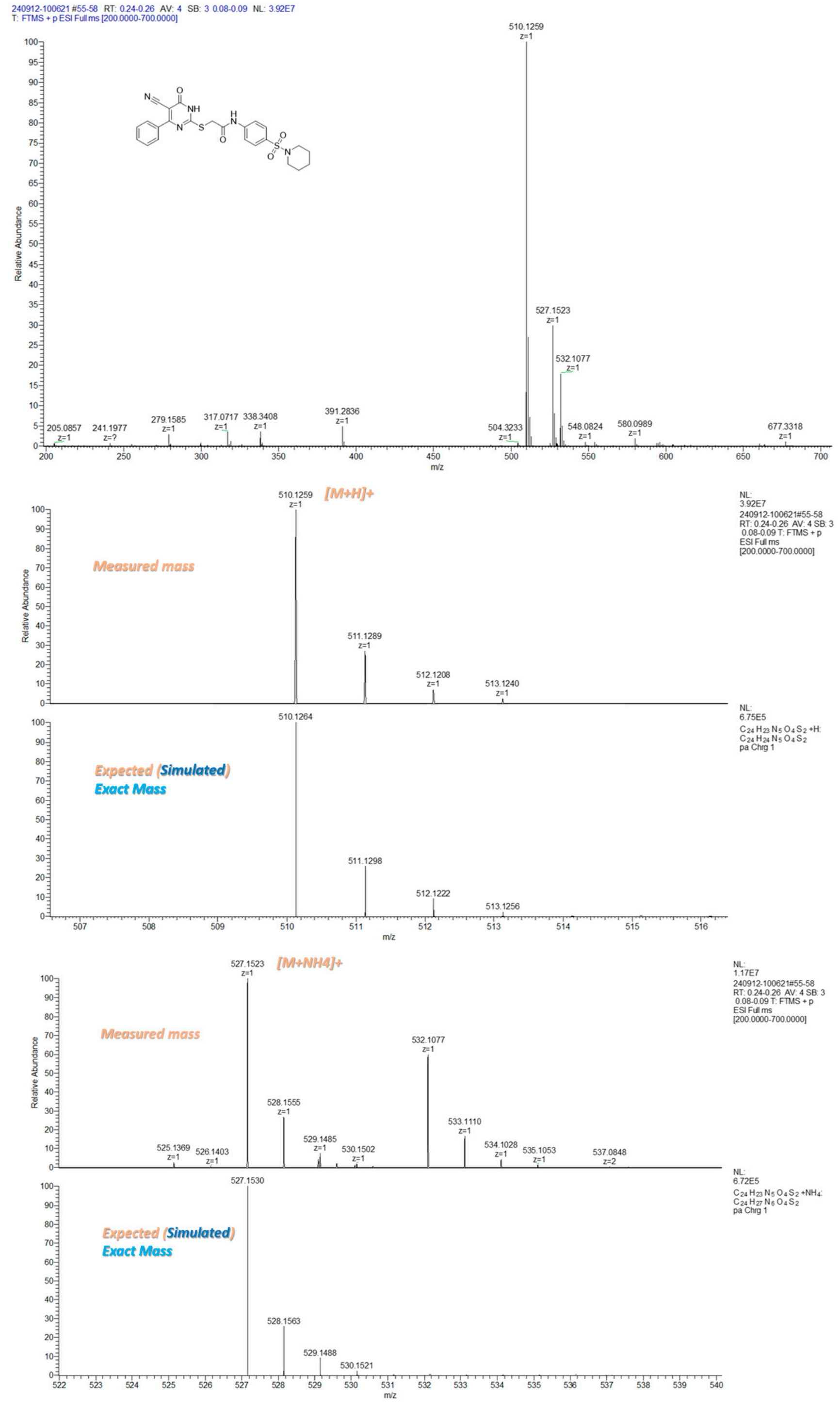

Figure S96. Mass analysis of compound M2

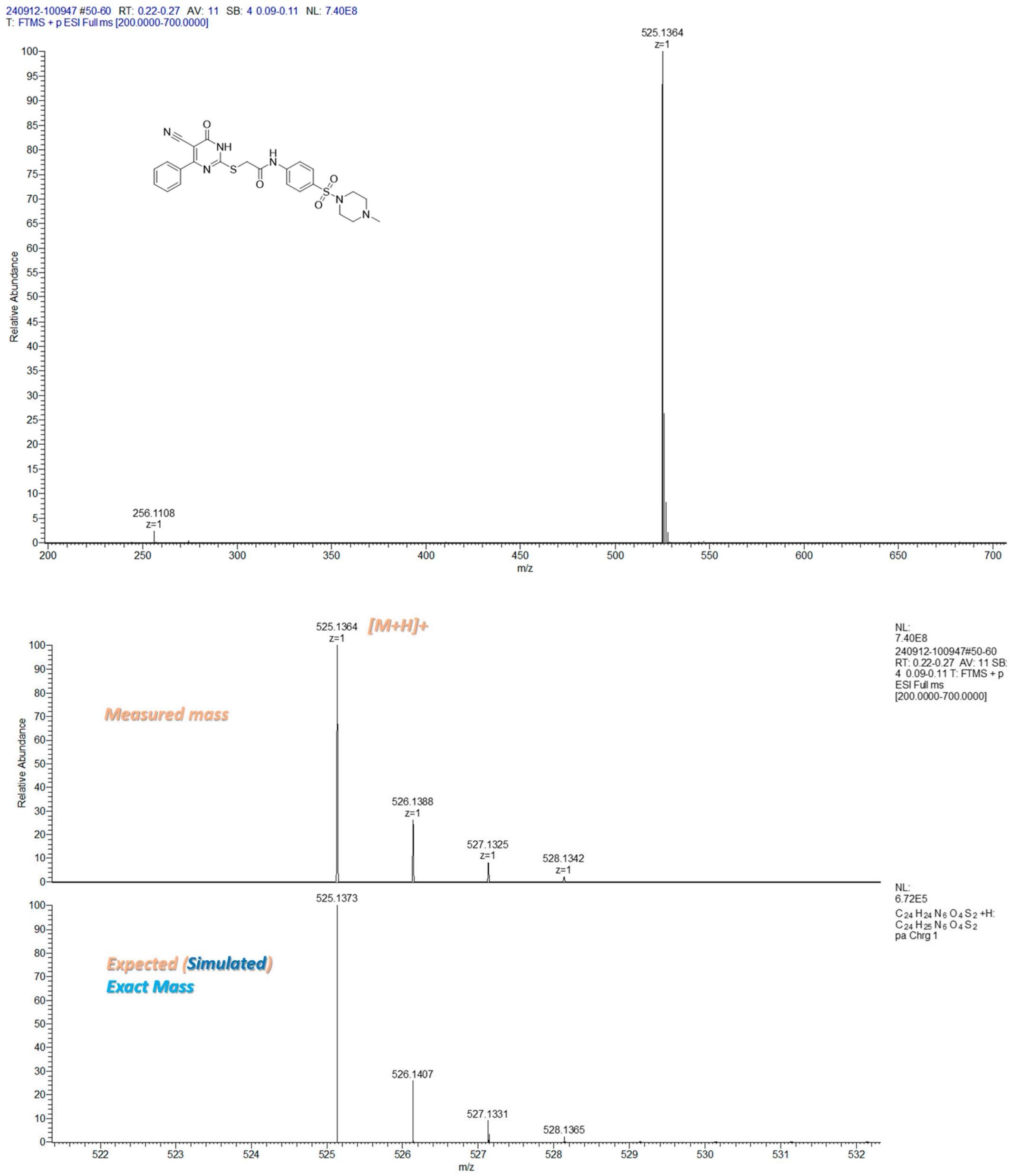

Figure S97. Mass analysis of compound M3

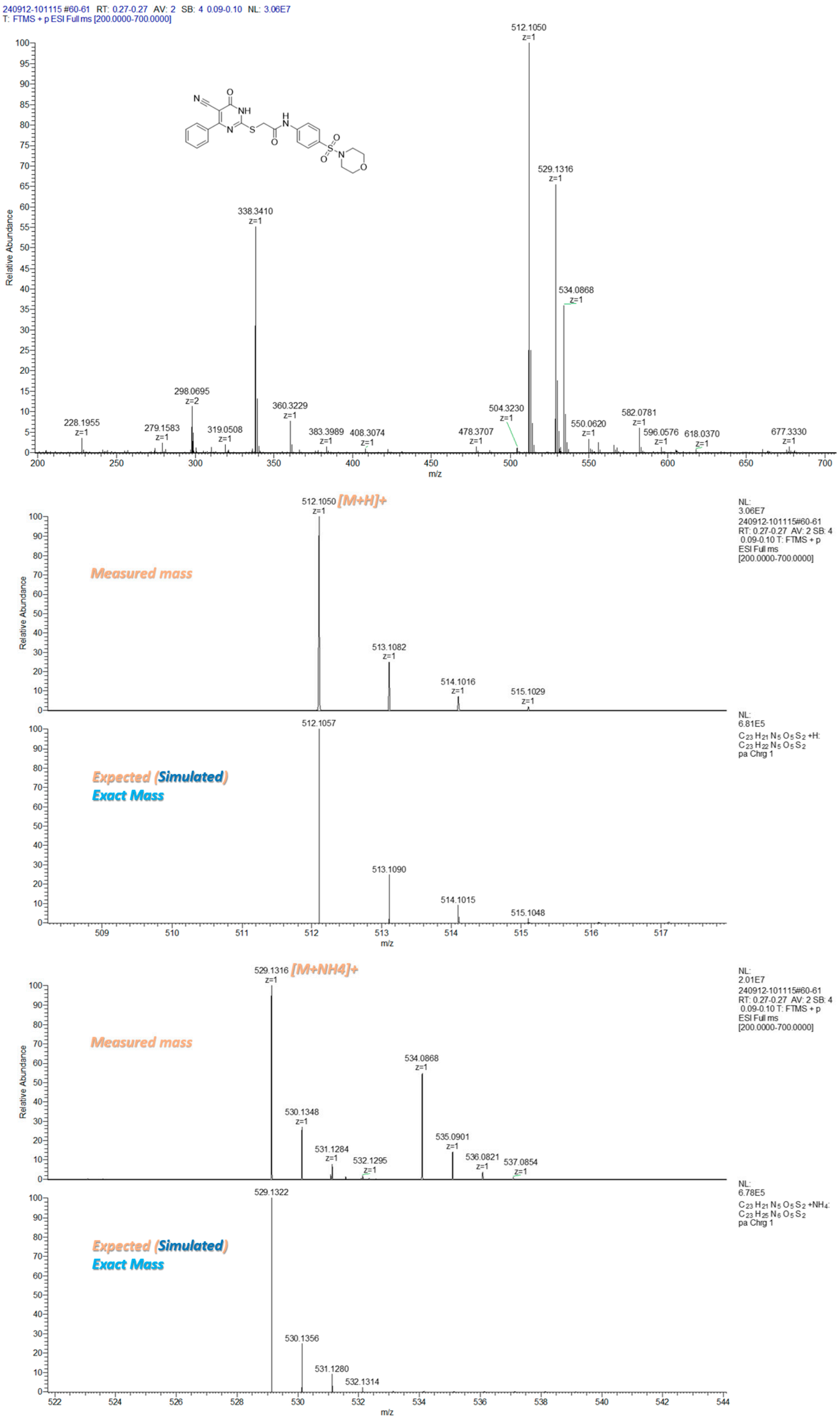

Figure S98. Mass analysis of compound M4

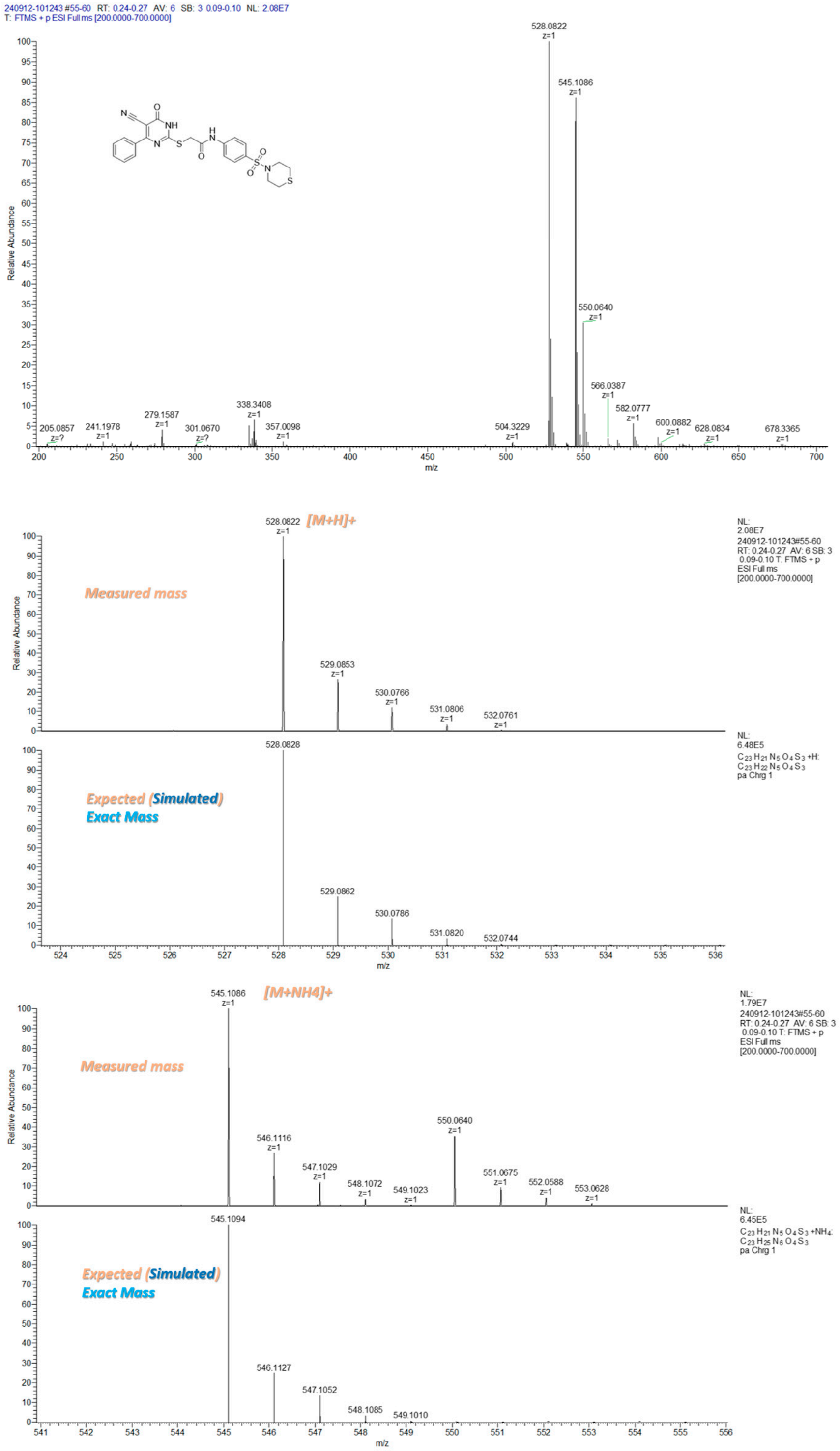

Figure S99. Mass analysis of compound M5

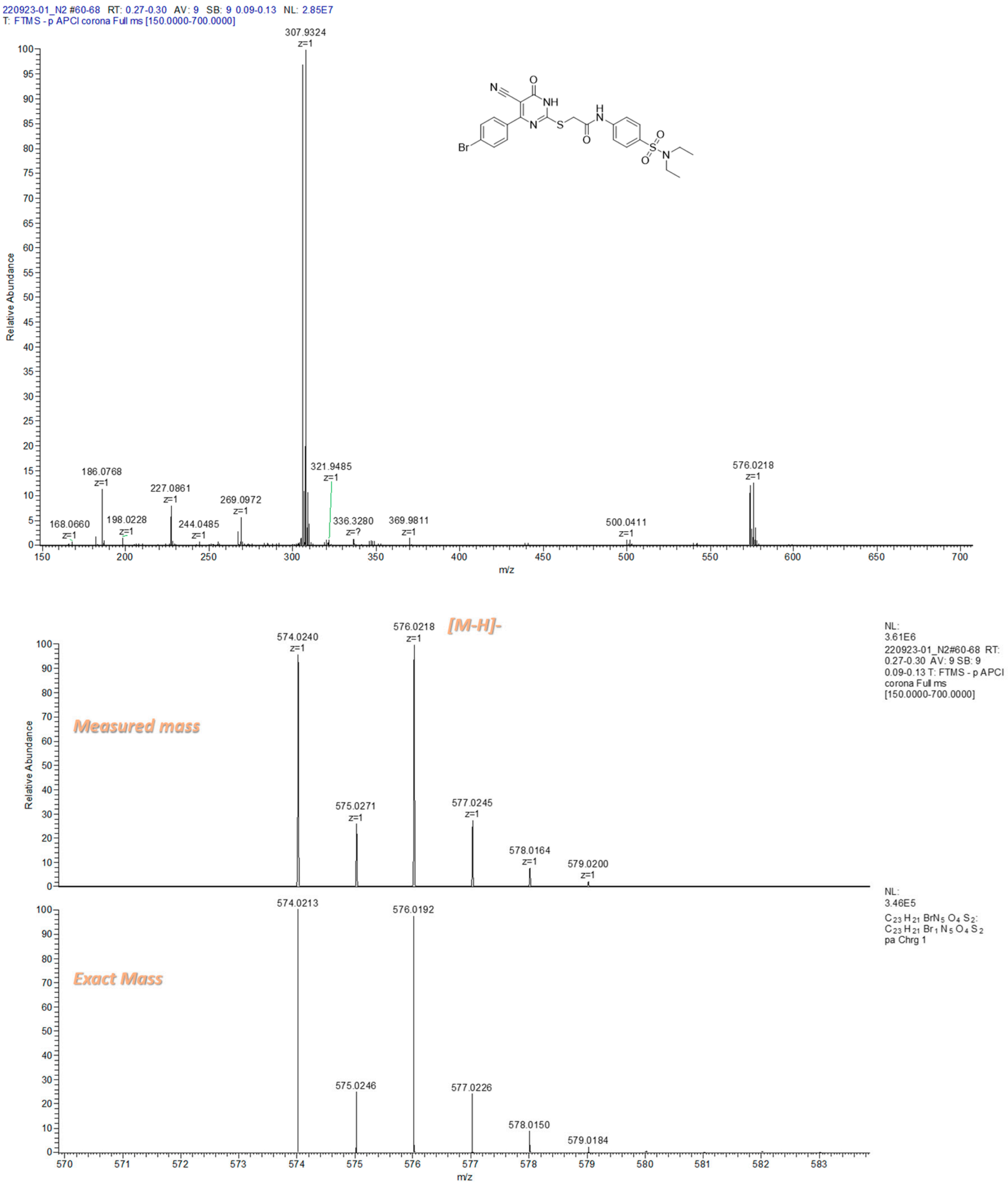

Figure S100. Mass analysis of compound M6

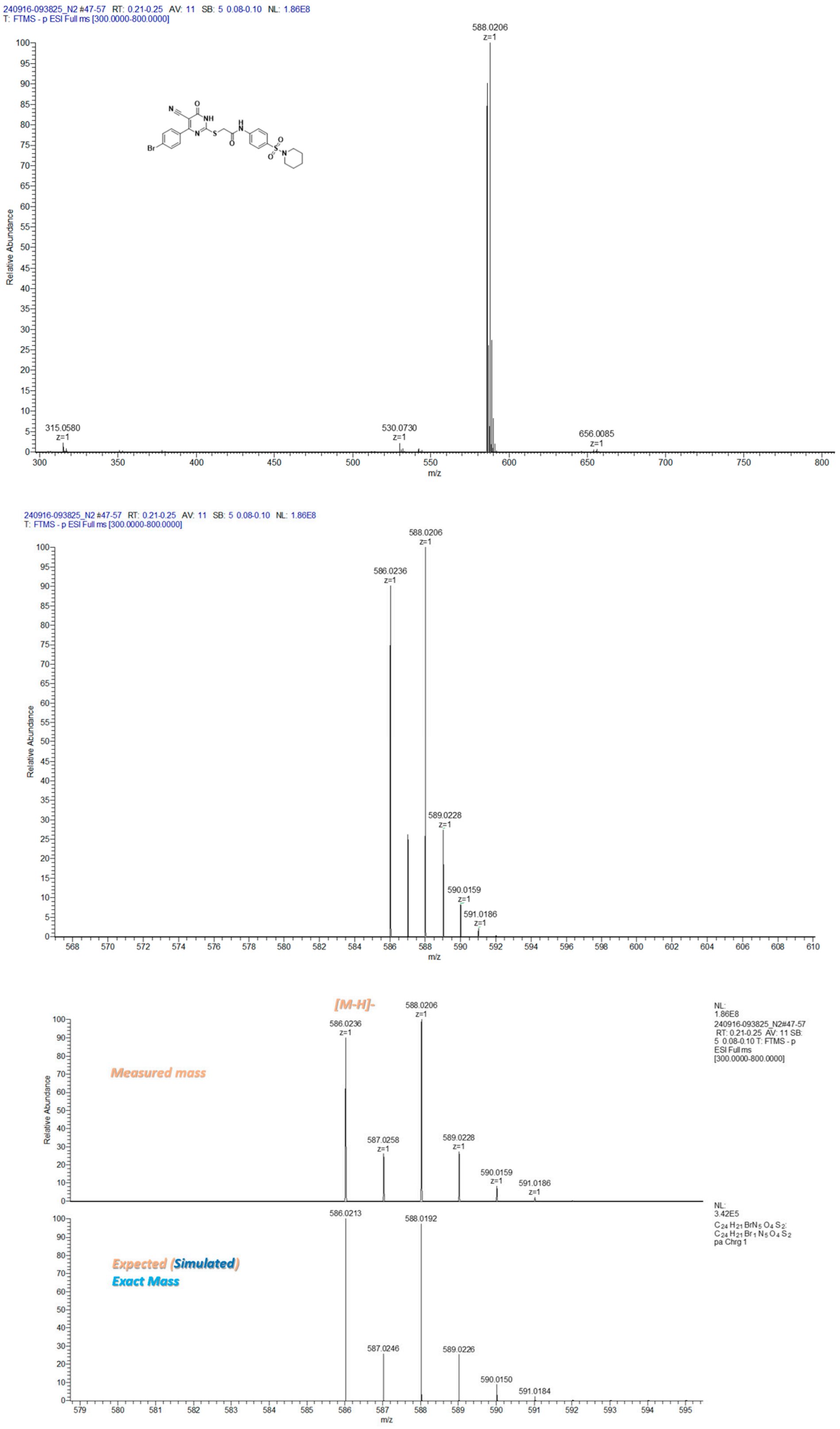

Figure S101. Mass analysis of compound M7

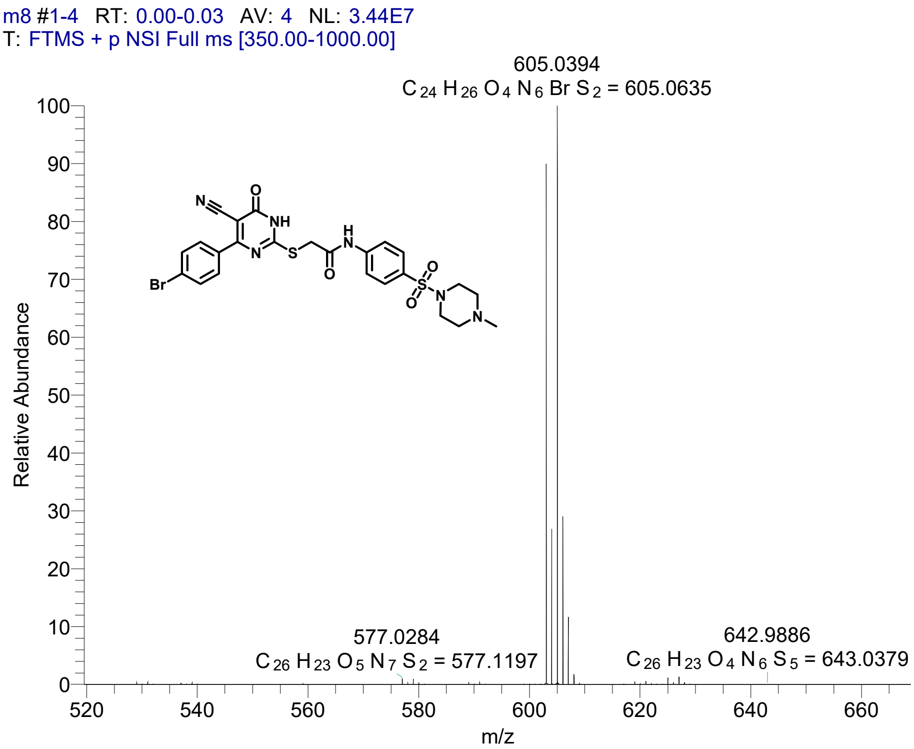

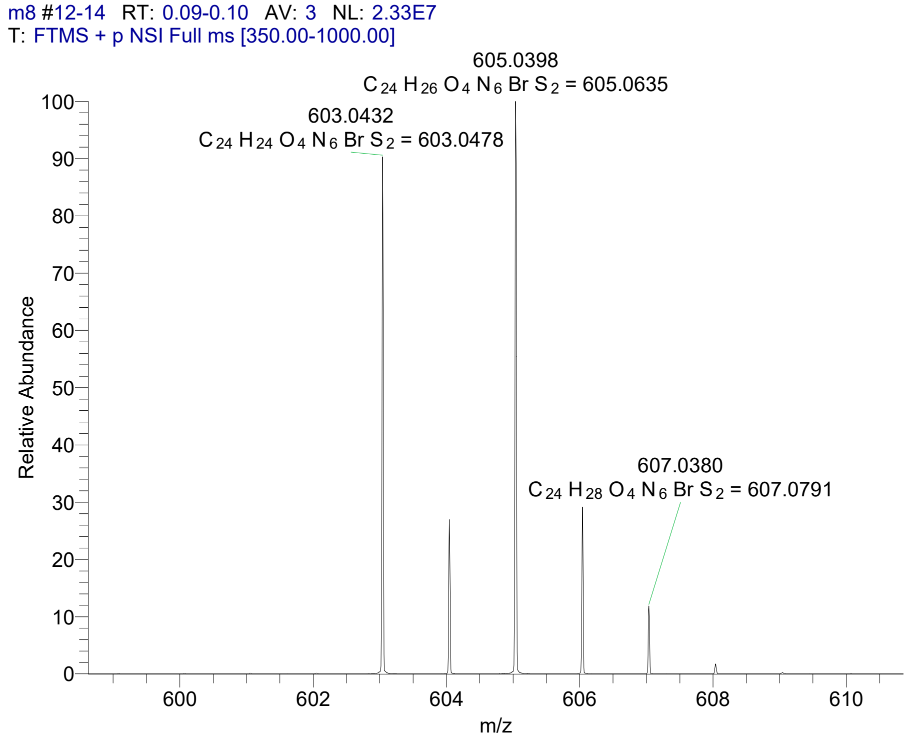

Figure S103. Mass analysis of compound M8

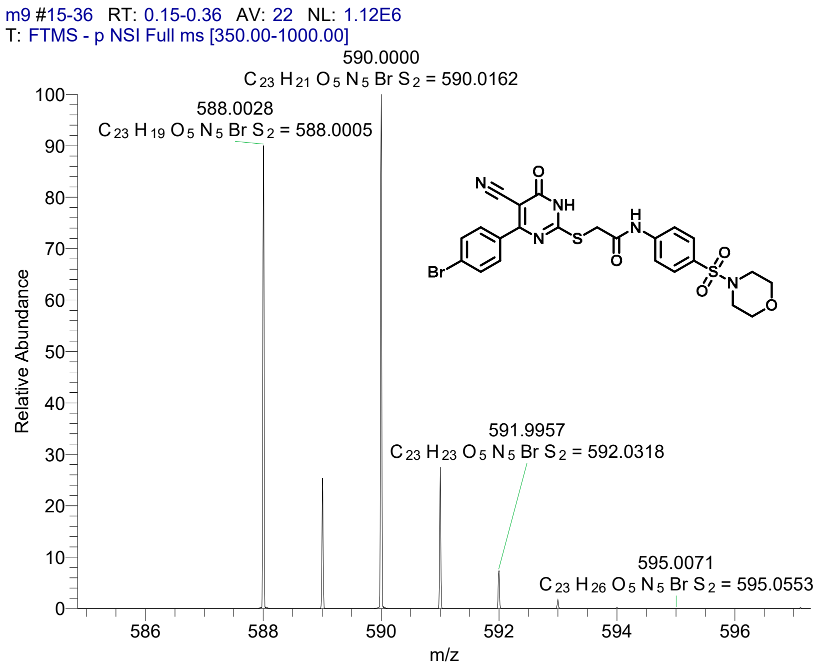

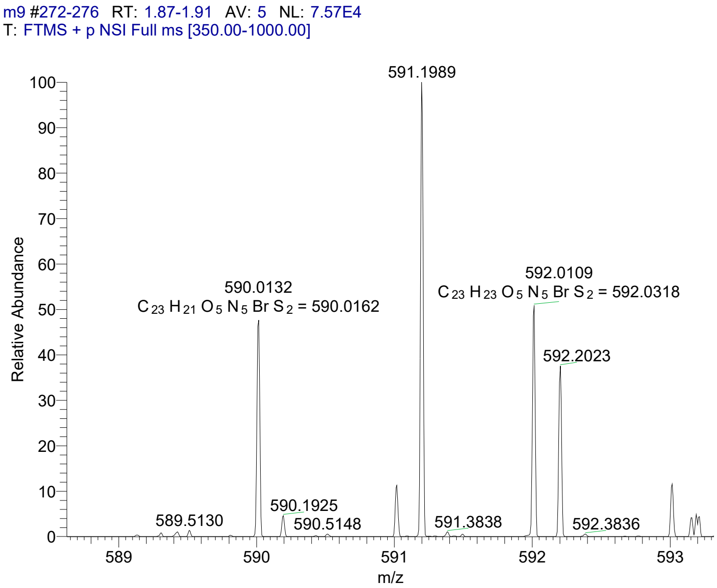

Figure S104. Mass analysis of compound M9

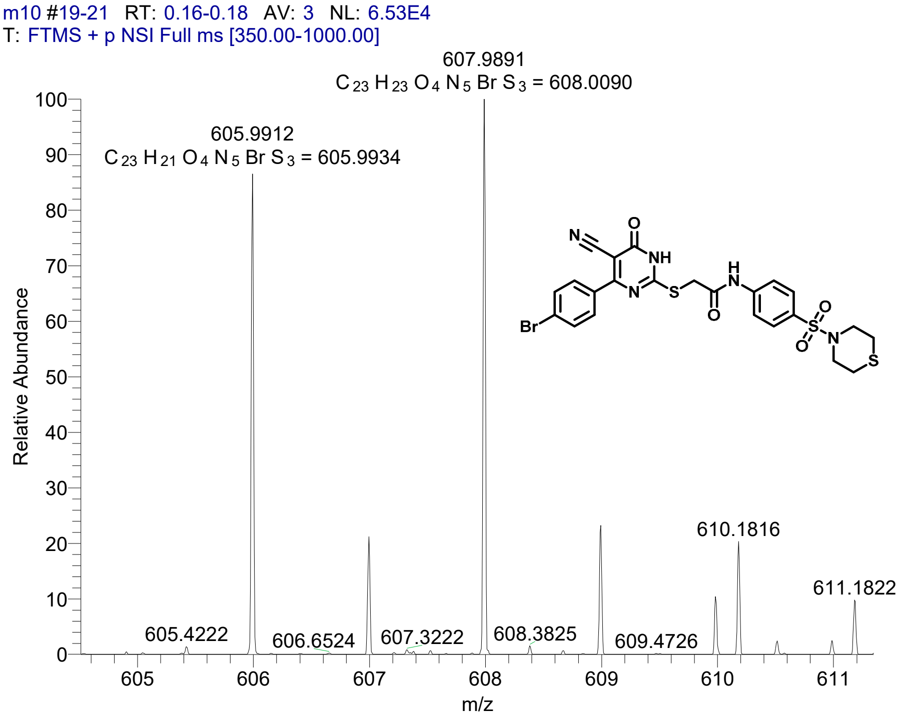

Figure S105. Mass analysis of compound M10

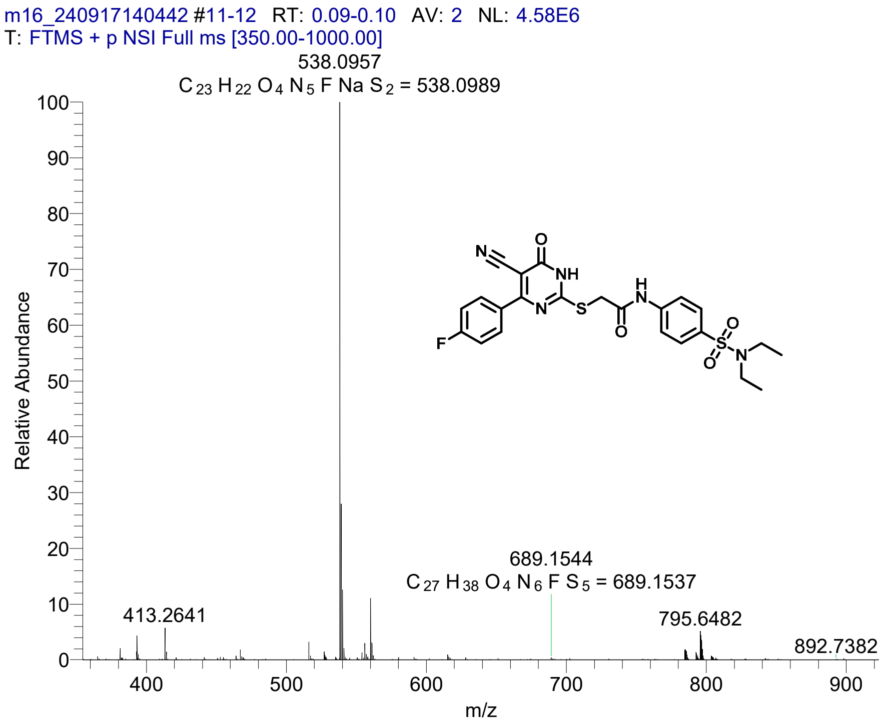

Figure S106. Mass analysis of compound M16

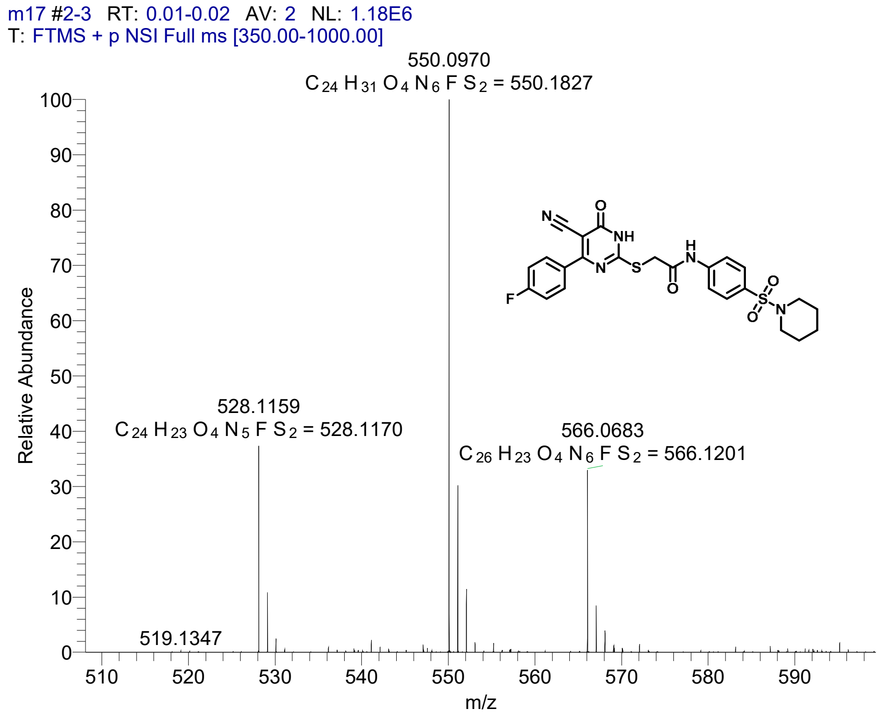

Figure S107. Mass analysis of compound M17

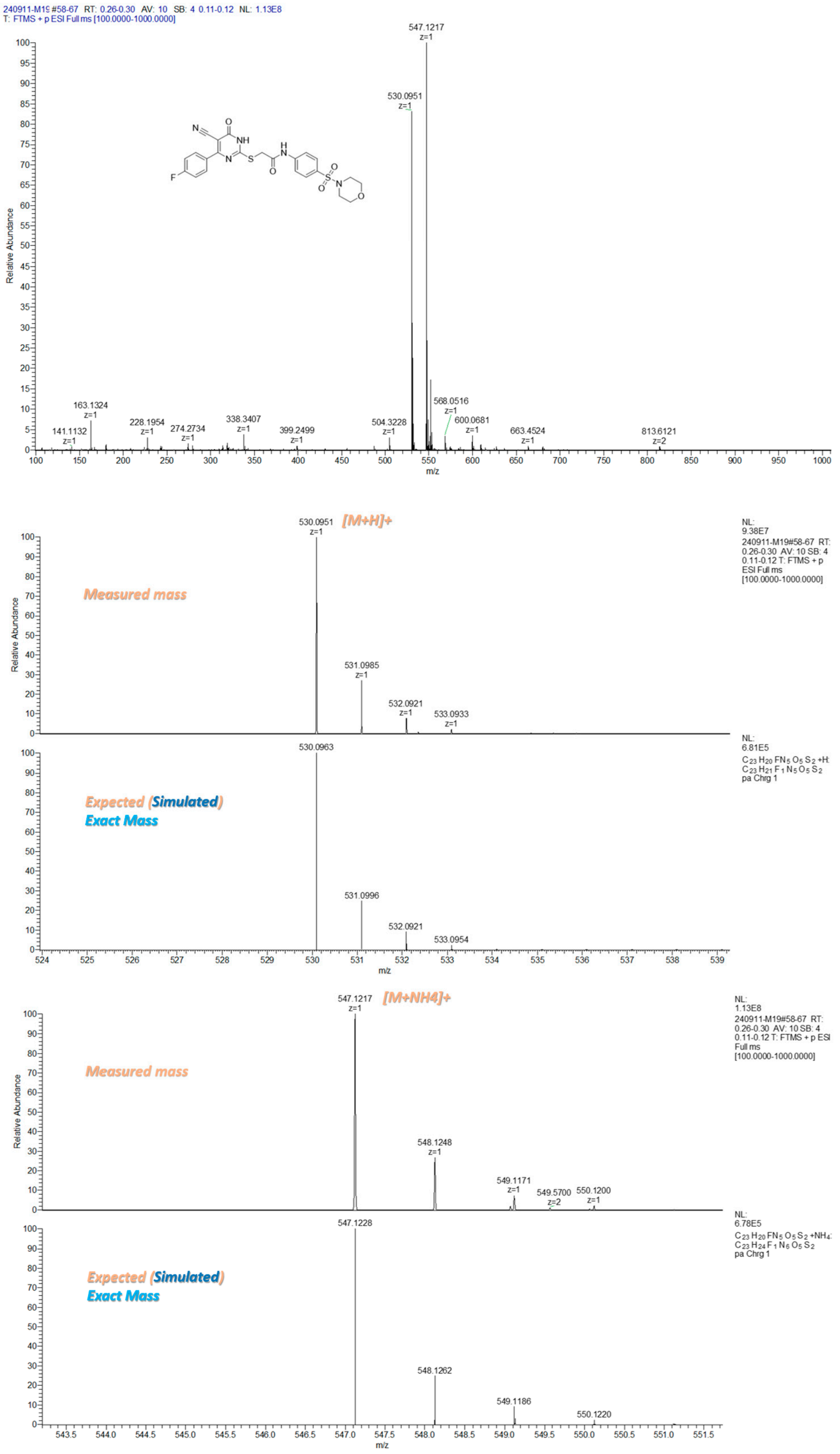

Figure S108. Mass analysis of compound M19

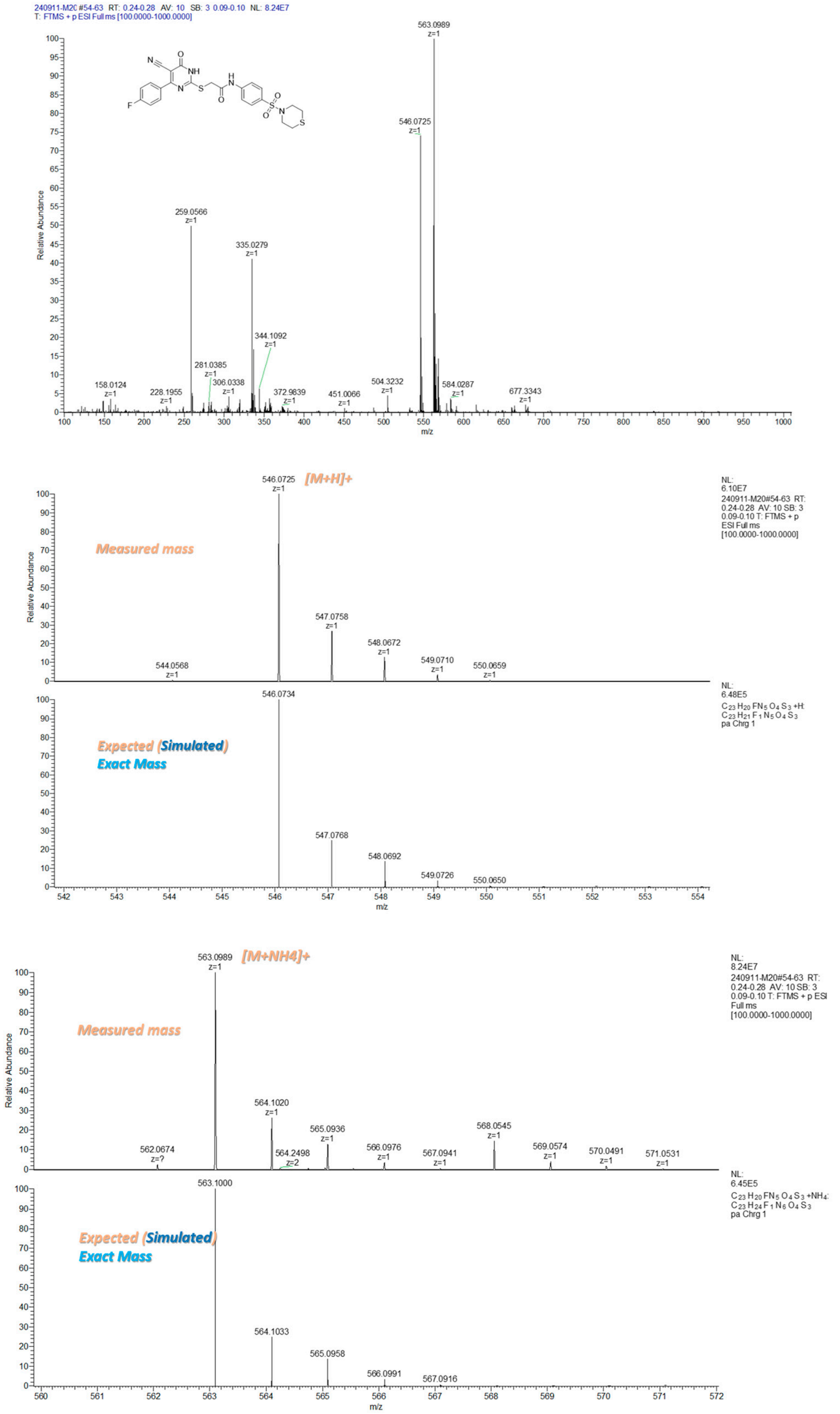

Figure S109. Mass analysis of compound M20

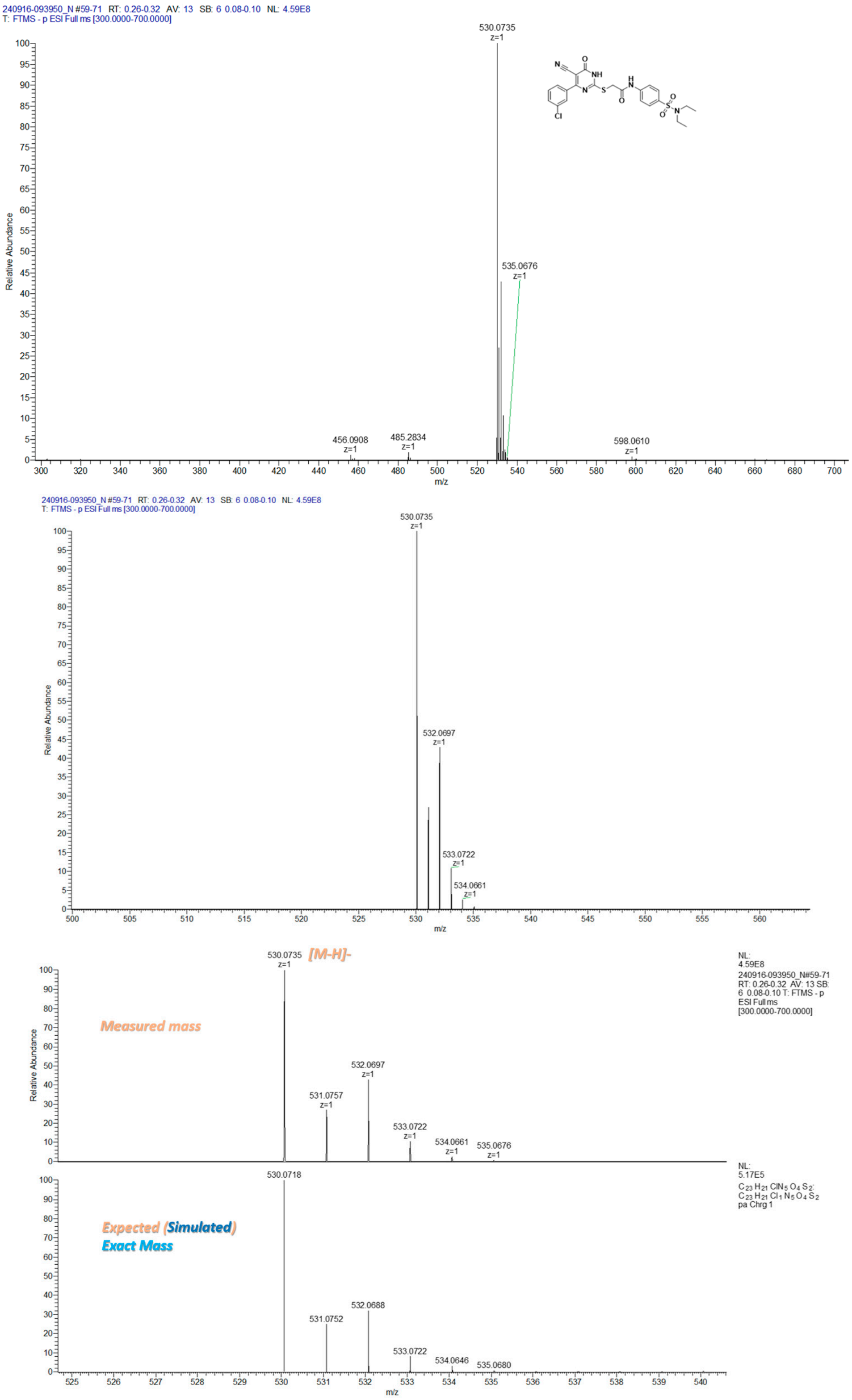

Figure S110. Mass analysis of compound M21

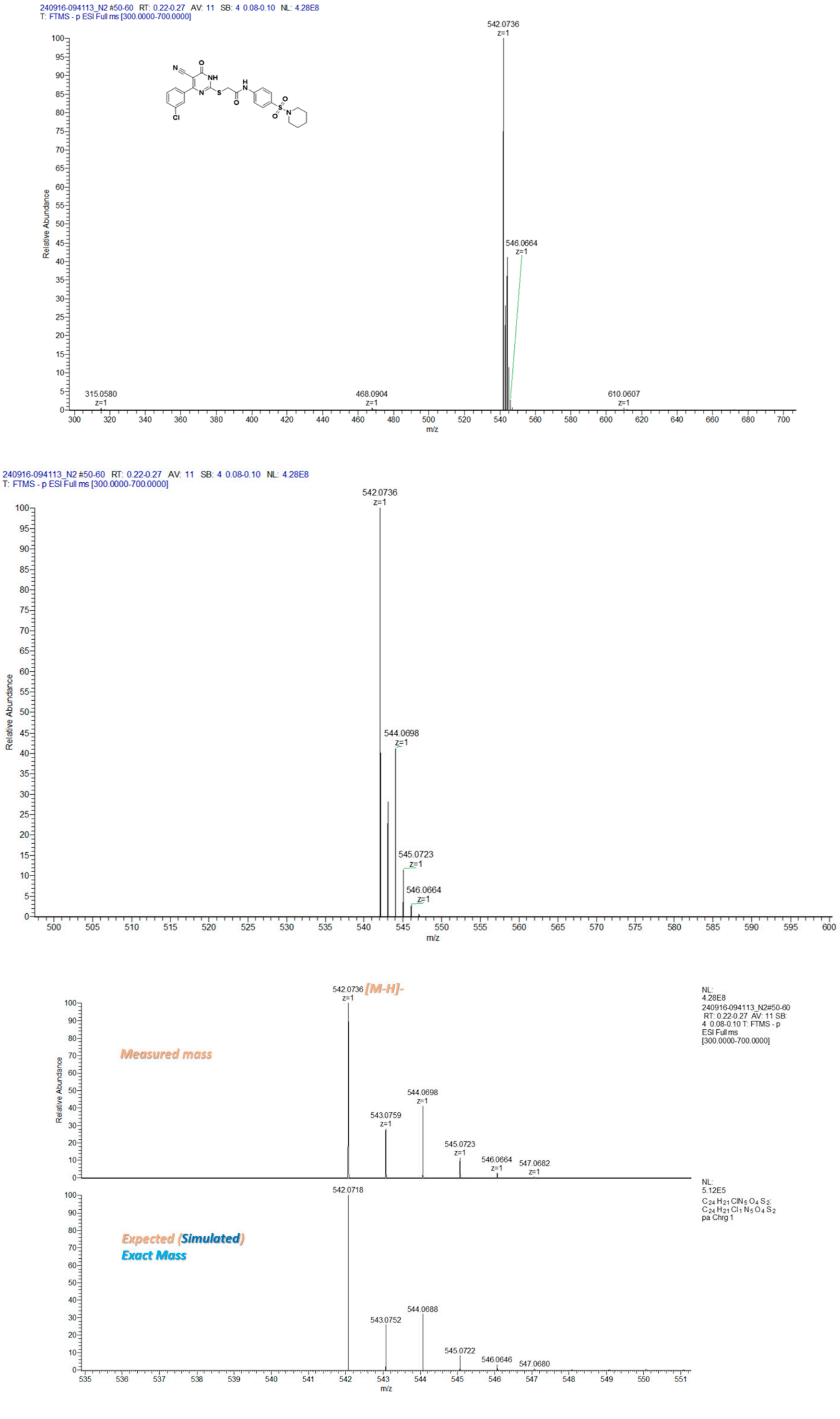

Figure S111. Mass analysis of compound M22

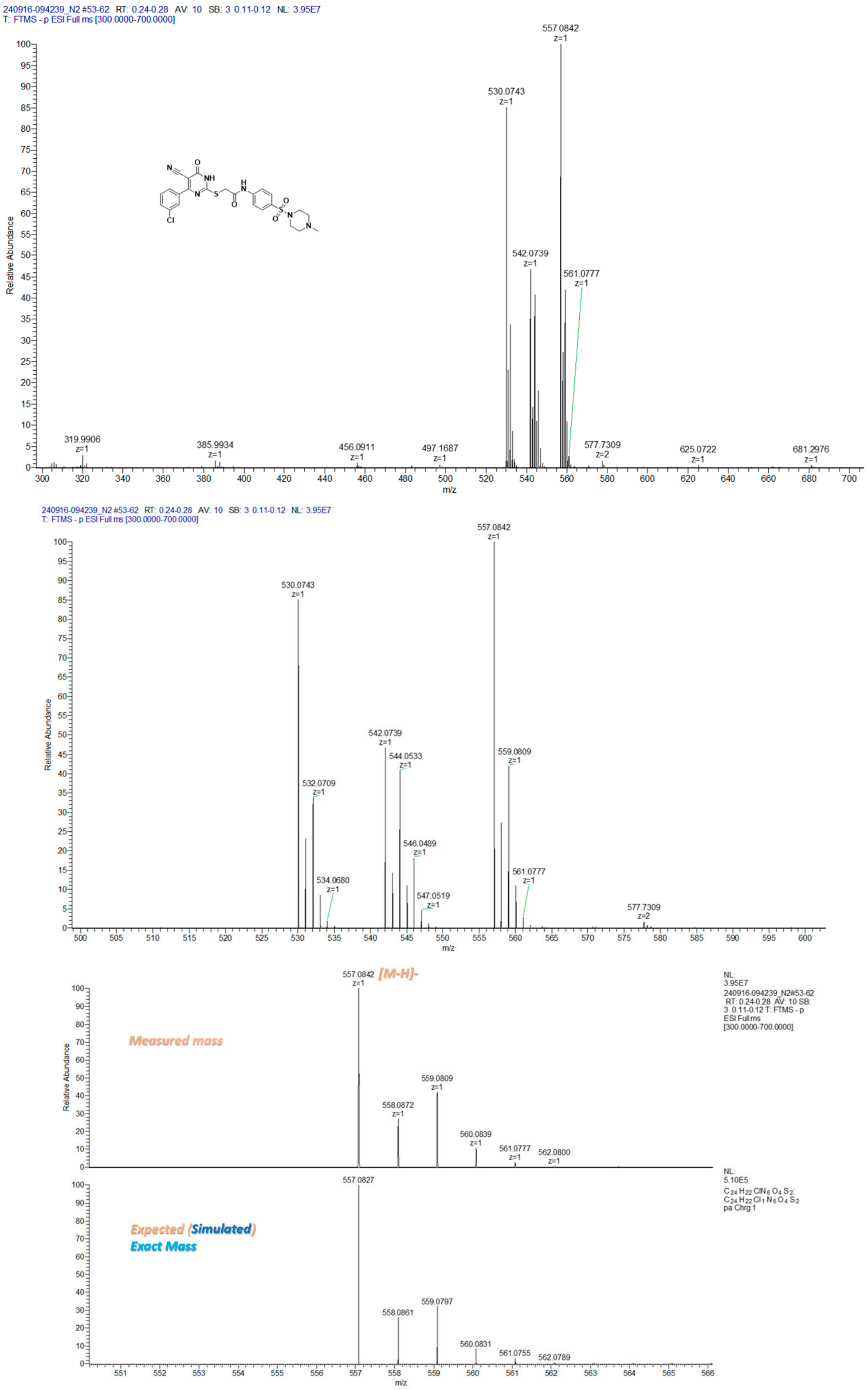

Figure S112. Mass analysis of compound M23

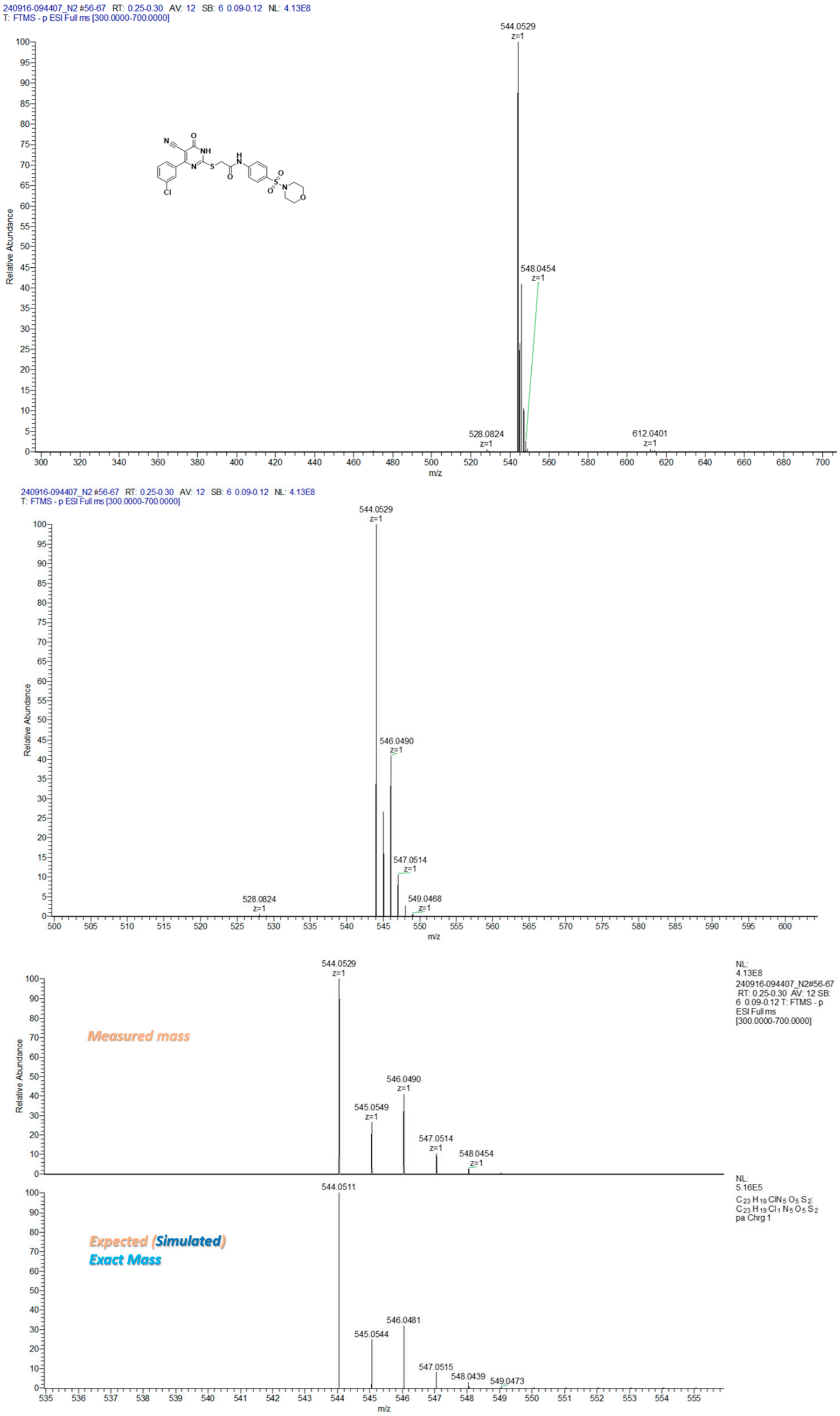

Figure S113. Mass analysis of compound M24

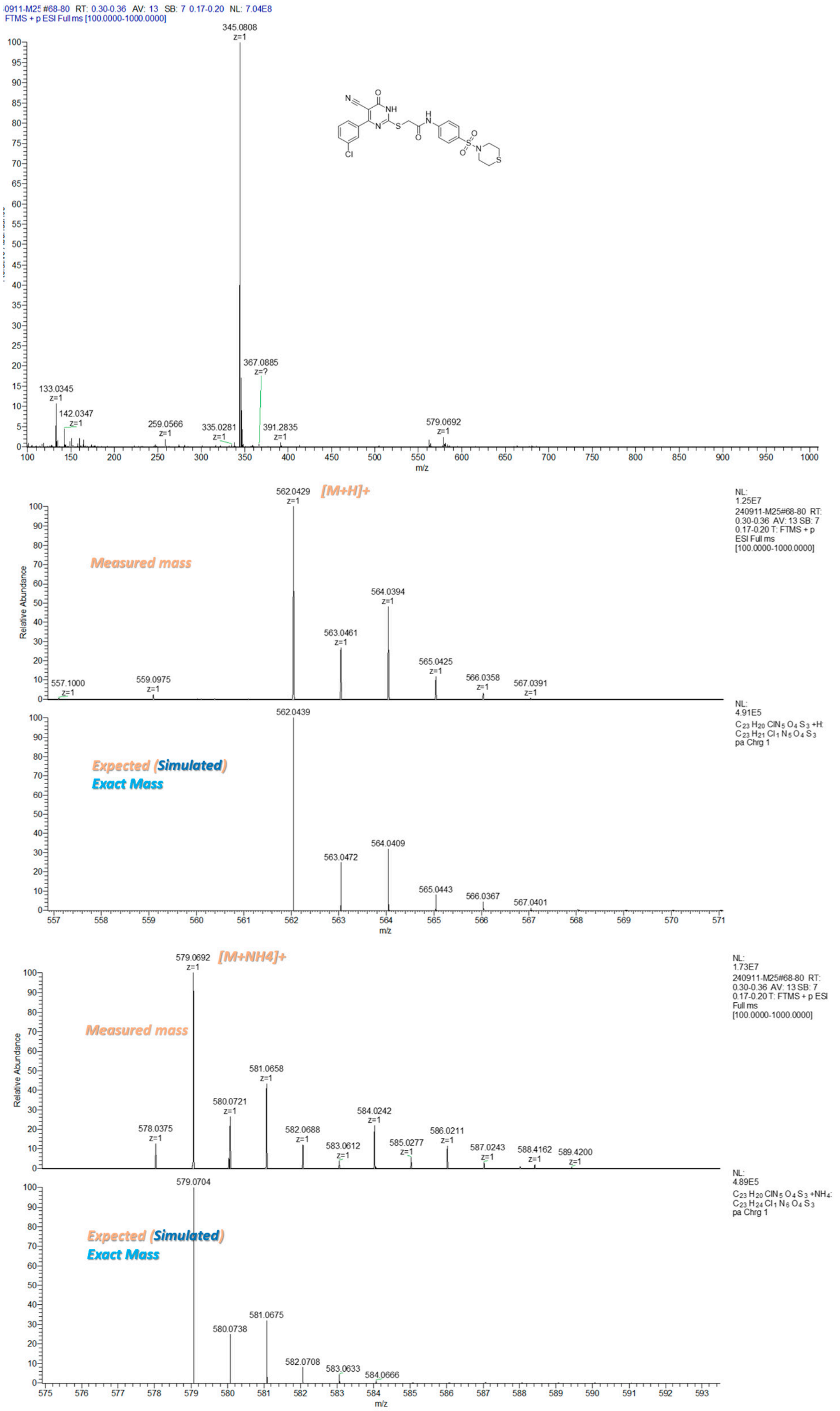

Figure S114. Mass analysis of compound M25

References

1. Riquelme, Sebastian A., D. Ahn, and A. Prince, *Pseudomonas aeruginosa and Klebsiella pneumoniae Adaptation to Innate Immune Clearance Mechanisms in the Lung.* Journal of Innate Immunity, 2018. **10**(5-6): p. 442-454.
2. Jondle, C.N., et al., *Klebsiella pneumoniae infection of murine neutrophils impairs their efferocytic clearance by modulating cell death machinery.* PLoS Pathog, 2018. **14**(10): p. e1007338.
3. Princiotto, S., et al., *The antimicrobial potential of adarotene derivatives against Staphylococcus aureus strains.* Bioorganic Chemistry, 2024. **145**: p. 107227.
4. Tangadanchu, V.K.R., Y.F. Sui, and C.H. Zhou, *Isatin-derived azoles as new potential antimicrobial agents: Design, synthesis and biological evaluation.* Bioorg Med Chem Lett, 2021. **41**: p. 128030.
5. Mirghani, R., et al., *Biofilms: Formation, drug resistance and alternatives to conventional approaches.* AIMS Microbiol, 2022. **8**(3): p. 239-277.
6. Mohsenipour, Z. and M. Hassanshahian, *The Effects of Allium sativum Extracts on Biofilm Formation and Activities of Six Pathogenic Bacteria.* Jundishapur J Microbiol, 2015. **8**(8): p. e18971.
7. Mounir, R., et al., *Unlocking the Power of Onion Peel Extracts: Antimicrobial and Anti-Inflammatory Effects Improve Wound Healing through Repressing Notch-1/NLRP3/Caspase-1 Signaling.* Pharmaceuticals, 2023. **16**(10): p. 1379.
8. Roskoski, R., Jr., *Properties of FDA-approved small molecule protein kinase inhibitors.* Pharmacol Res, 2019. **144**: p. 19-50.
